# Multimodal learning enables chat-based exploration of single-cell data

**DOI:** 10.1038/s41587-025-02857-9

**Published:** 2025-11-11

**Authors:** Moritz Schaefer, Peter Peneder, Daniel Malzl, Salvo Danilo Lombardo, Mihaela Peycheva, Jake Burton, Anna Hakobyan, Varun Sharma, Thomas Krausgruber, Celine Sin, Jörg Menche, Eleni M. Tomazou, Christoph Bock

**Affiliations:** 1 Medical University of Vienna, Institute of Artificial Intelligence, Center for Medical Data Science (CEDAS), Vienna, Austria; 2 CeMM Research Center for Molecular Medicine of the Austrian Academy of Sciences, Vienna, Austria; 3 St. Anna Children’s Cancer Research Institute (CCRI), Vienna, Austria; 4 Doctoral School in Microbiology and Environmental Science, University of Vienna, Vienna, Austria; 5 Max Perutz Labs, Vienna, Austria; 6 Department of Structural and Computational Biology, Center for Molecular Biology, University of Vienna, Vienna, Austria; 7 Ludwig Boltzmann Institute for Network Medicine at the University of Vienna, Vienna, Austria; 8 Faculty of Mathematics, University of Vienna, Vienna, Austria; 9 Medical University of Vienna, Center for Cancer Research, Vienna, Austria; 10 Medical University of Vienna, Comprehensive Center for AI in Medicine (CAIM), Vienna, Austria

**Keywords:** Machine learning, Transcriptomics, Software, Gene regulation in immune cells, Preclinical research

## Abstract

Single-cell sequencing characterizes biological samples at unprecedented scale and detail, but data interpretation remains challenging. Here, we present CellWhisperer, an artificial intelligence (AI) model and software tool for chat-based interrogation of gene expression. We establish a multimodal embedding of transcriptomes and their textual annotations, using contrastive learning on 1 million RNA sequencing profiles with AI-curated descriptions. This embedding informs a large language model that answers user-provided questions about cells and genes in natural-language chats. We benchmark CellWhisperer’s performance for zero-shot prediction of cell types and other biological annotations and demonstrate its use for biological discovery in a meta-analysis of human embryonic development. We integrate a CellWhisperer chat box with the CELLxGENE browser, allowing users to interactively explore gene expression through a combined graphical and chat interface. In summary, CellWhisperer leverages large community-scale data repositories to connect transcriptomes and text, thereby enabling interactive exploration of single-cell RNA-sequencing data with natural-language chats.

## Abstract (web summary)

CellWhisperer uses multimodal learning of transcriptomes and text to answer questions about single-cell RNA-sequencing data.

## Main

Gene expression profiling is widely used for the characterization of cells and tissues^1,2^. Bulk RNA sequencing (RNA-seq) provides a detailed assessment of cell states and biological functions through a straightforward and cost-effective assay^3^. Moreover, with single-cell RNA sequencing (scRNA-seq), researchers can disentangle the cell composition and the biological heterogeneity of tissues, organs and diseases^4^. Large-scale scRNA-seq is also at the heart of the Human Cell Atlas and its mission to create reference maps of all cell types in the human body^5^.

A typical scRNA-seq dataset can be represented by a count matrix with ~20,000 genes and thousands or millions of single cells. Analyzing and interpreting such datasets are complex tasks that require both bioinformatic skills and application-specific biological domain knowledge. To facilitate scRNA-seq data analysis, software tools have been developed for a wide range of tasks including data visualization, cell clustering, cell type annotation, differential expression and gene set analysis^6^. Moreover, deep-learning-based ‘single-cell foundation models’ (scFMs) have been trained on large scRNA-seq datasets, with the promise of going beyond specialized tools and supporting a wide range of analysis tasks that they were not explicitly optimized for^7,8^.

Here, we demonstrate scRNA-seq data exploration with natural language, allowing the user to interrogate cells in English, with no need to adhere to any particular format or syntax. Our CellWhisperer framework supports free-text search (such as ‘Show me tissue-resident T cells in the intestine’) and answers a broad range of questions about cells (for example, ‘What are these selected cells?’, ‘Which genes are highly expressed in these cells?’, ‘What is the role of KLRD1 in natural killer (NK) cells?’). The model’s responses are based on the combination of selected scRNA-seq data and the biological knowledge of a large language model (LLM), resulting in answers such as ‘The selected cells appear to be CD16^+^ NK cells, which are a subset of NK cells that have a crucial role in the innate immune response […]’, ‘The top expressed genes in these cells include *NKG7*, *KLRD1*, *GNLY*, *GZMA*, *PRF1* […]’, ‘KLRD1 (CD94) is a receptor that has a role in NK cell activation and cytotoxicity. It can recognize MHC class I molecules on target cells and trigger NK-cell-mediated cytotoxicity’.

CellWhisperer implements this functionality with two intertwined artificial intelligence (AI) models. First, the CellWhisperer embedding model integrates RNA profiles and their metadata-derived textual annotations through multimodal contrastive learning^9^, creating a joint multimodal embedding of transcriptomes and text. CellWhisperer’s training data comprise over a million transcriptomes and their natural-language descriptions, prepared by AI-assisted curation from two large repositories: Gene Expression Omnibus (GEO)^10,11^ and CELLxGENE Census^12^. Second, the CellWhisperer chat model adapts an open-weights LLM^13,14^ to answer free-text questions about cell states while considering user-provided transcriptome profiles as multimodal input. Combining these two models, CellWhisperer enables interactive chat-based exploration of scRNA-seq data, which we integrated into the widely used CELLxGENE Explorer^15^. The CellWhisperer software, models, training data and source code are available online (https://cellwhisperer.bocklab.org) and usage examples are provided in Fig. 5 and Supplementary Note 1.

In summary, we developed CellWhisperer as a proof of concept for natural language as an intuitive channel to interact with scRNA-seq datasets (Supplementary Video 1). It is enabled by a multimodal AI model of transcriptomes and text, combined with the biological knowledge of an integrated chat model. We envision the interrogation of data through natural language as a key element of future AI-based bioinformatics research assistants.

## Results

### The CellWhisperer multimodal AI connects transcriptomes and text

**Fig. 1:**
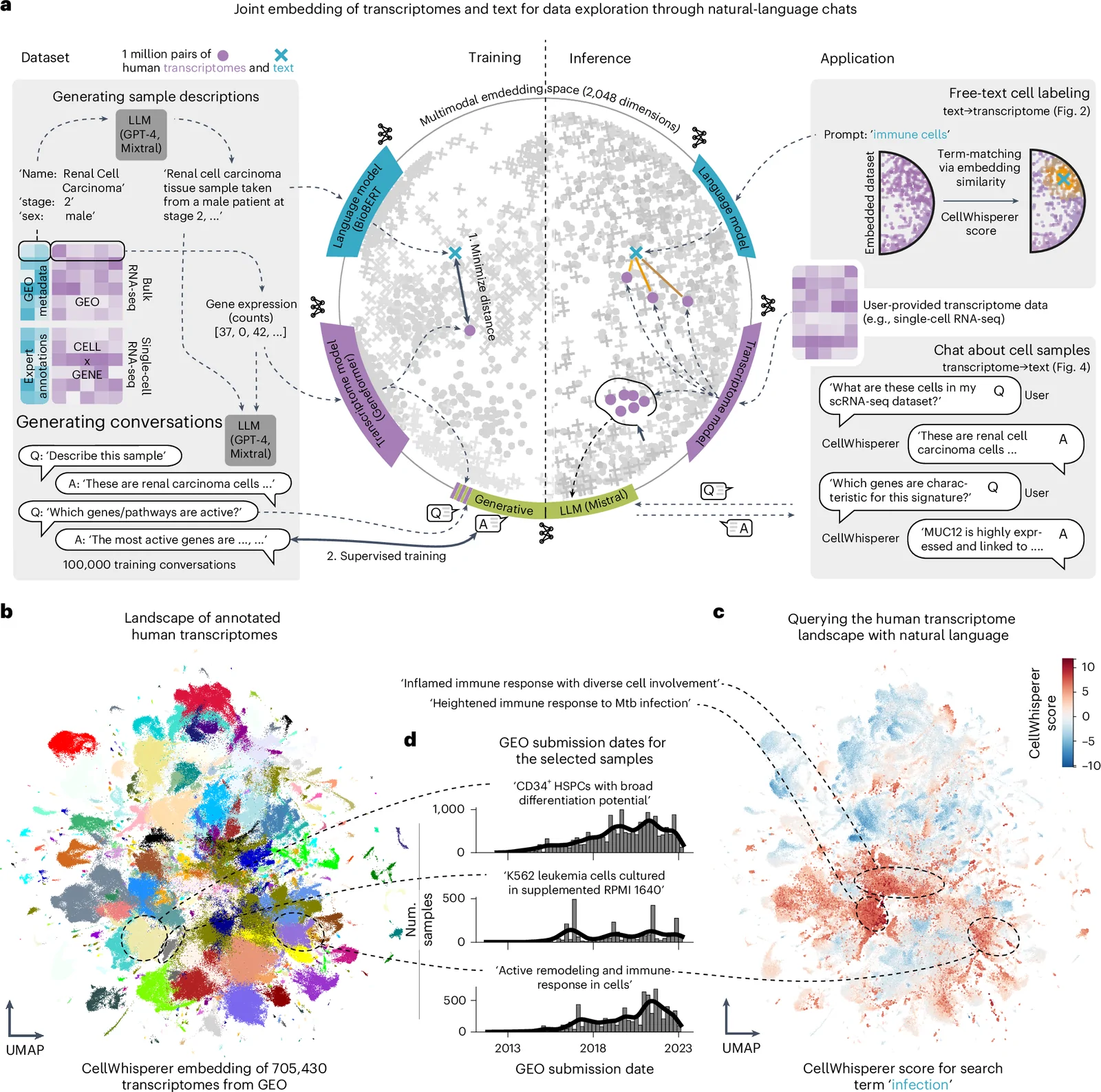
Overview of the CellWhisperer multimodal AI for natural-language analysis of transcriptome data. **a**, Conceptual outline of CellWhisperer training dataset generation (left), model training and inference (center) and applications in scRNA-seq data analysis (right). **b**, UMAP visualization of CellWhisperer embeddings for human transcriptomes from the GEO repository. Clusters were computed using the Leiden algorithm and cluster labels were generated by CellWhisperer. The CellWhisperer-annotated dataset is available for interactive analysis on the project website (https://cellwhisperer.bocklab.org/geo). **c**, CellWhisperer scores for the free-text query term ‘infection’ projected on the UMAP of transcriptome embeddings from **b**. **d**, Retrieval of sample metadata (here: GEO submission date) for transcriptomes selected by CellWhisperer-generated cluster labels.

**Extended Data Fig. 1:**
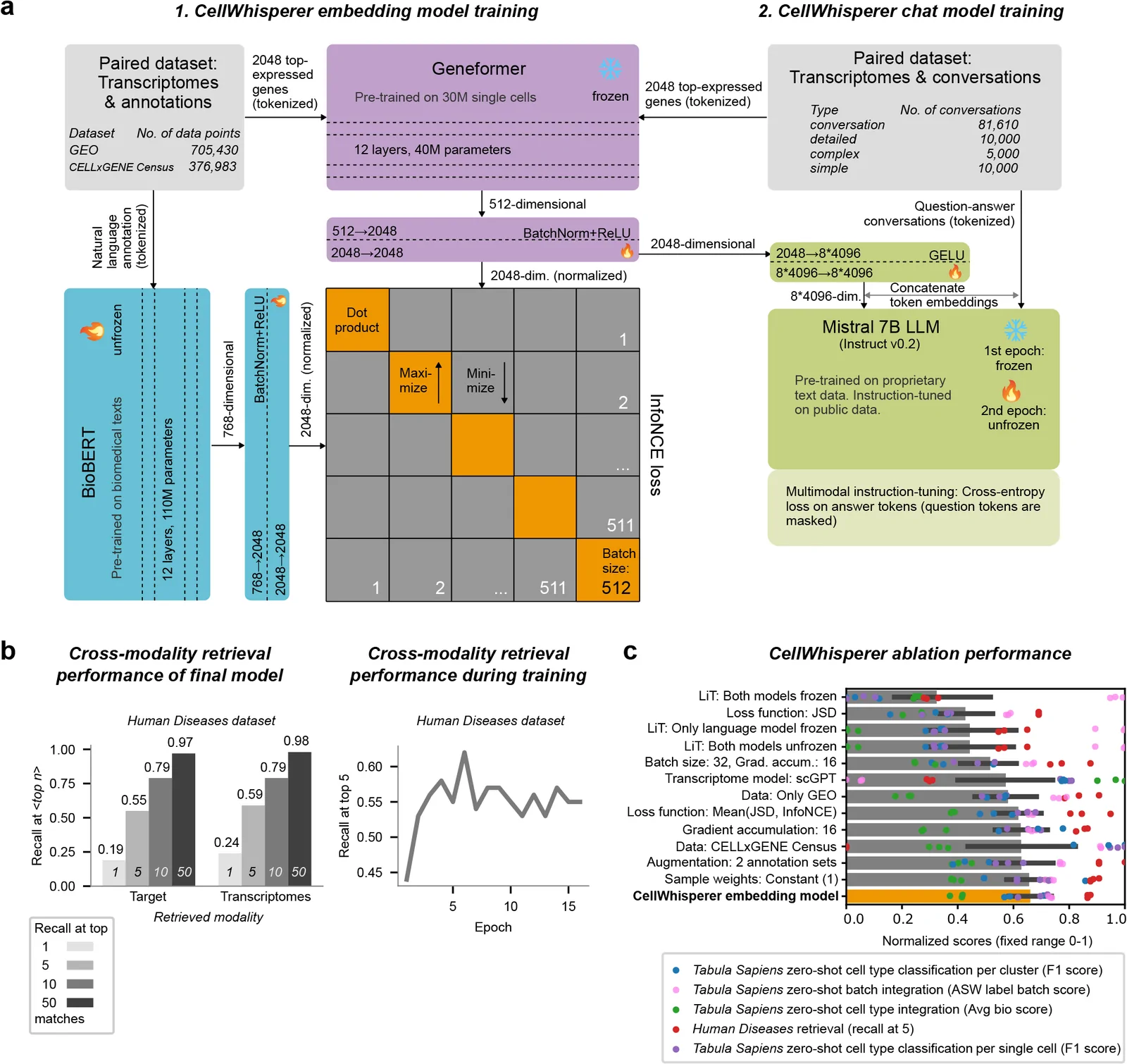
CellWhisperer architecture, model training and performance evaluation. **a**) Architecture and training of the multimodal CellWhisperer embedding and chat models. Ice and fire icons indicate components that are frozen and unfrozen during training. **b**) Cross-modality retrieval performance of the CellWhisperer embedding model, evaluated on the deduplicated Human Diseases dataset for the trained model (left) and during training (right). **c**) Performance comparison of alternative architectures and hyperparameter choices in an ablation study of the CellWhisperer embedding model. Barplots show the mean performance for five metrics and three replicate models trained with different seeds. The values for each metric (shown as dots) were min-max normalized across models. F1 scores were macro-averaged across classes. The bar plots indicate means across all data points, with error bars corresponding to 95% confidence intervals across all 15 data points. The final CellWhisperer embedding model (marked in bold) was trained with batch size 512, no gradient accumulation, frozen Geneformer transcriptome model, InfoNCE loss, GEO and CELLxGENE Census datasets, density-based sample weighting and no data augmentation. JSD: Jensen-Shannon divergence information maximization loss. InfoNCE: information noise contrastive estimation.

We present CellWhisperer, a multimodal AI that enables interactive scRNA-seq data exploration with natural-language conversations. Our method was created in three steps (Fig. 1a): (1) LLM-assisted curation of multimodal training data, resulting in 1,082,413 pairs of human RNA-seq profiles and matched textual annotations; (2) training of the CellWhisperer embedding model, which places the transcriptomes and their AI-curated textual descriptions into a joint embedding space for cell search and annotation; and (3) development of the CellWhisperer chat model for transcriptome-aware question answering and natural-language chats. This section summarizes each of these three steps, while further technical details are provided in the Methods, Supplementary Notes 2 and 3 and Extended Data Fig. 1.

First, we created a large training dataset of transcriptomes (including bulk RNA-seq profiles and scRNA-seq derived pseudo-bulk profiles) with concise textual annotations (such as ‘Renal cell carcinoma tissue sample taken from a male individual at stage 2, with no metastasis, preserved in formalin-fixed paraffin-embedded blocks’) across the wide range of cell types and conditions captured by GEO and CELLxGENE Census. GEO comprises human RNA-seq data from more than 20,000 individual studies based on researcher submissions, which provides tremendous thematic breadth but also a need for data harmonization. We used the ARCHS4 uniform reprocessing of GEO data^16^ and developed an LLM-assisted curation procedure to create concise, coherent and biologically informative textual annotations for each sample based on sample-specific metadata provided by GEO (which includes cell types, organs, tissues, diseases, experimental methods and scientific project abstracts). LLM prompts and illustrative results are shown in Supplementary Note 2. This AI-assisted data curation yielded a standardized dataset of 705,430 human transcriptomes with matched textual annotations.

We also derived pseudo-bulk transcriptomes from several hundred scRNA-seq datasets in the CELLxGENE Census, including reference maps from the Human Cell Atlas. We grouped the cells in each dataset on the basis of the provided metadata and calculated pseudo-bulk transcriptomes by averaging across all scRNA-seq profiles per group. We then applied our LLM-assisted curation procedure to condense the metadata for each group into concise biological descriptions, resulting in 376,983 human transcriptomes with matched textual annotations.

Second, we used the combined set of 1,082,413 annotated transcriptomes to train the multimodal CellWhisperer embedding model, which integrates the two data modalities into a joint embedding space (Extended Data Fig. 1a and Fig. 1a). To that end, we adapted the contrastive language image pretraining (CLIP) architecture^9^, processing the transcriptomes with the Geneformer model for gene expression^17^ and the textual annotations with the BioBERT model for biomedical text^18^. The two resulting vectors were mapped into a 2,048-dimensional multimodal embedding space using conventional feed-forward neural network layers. We then trained this model to place the two modality-specific embeddings in close proximity within the joint embedding space.

We validated that the resulting CellWhisperer embedding model was capable of retrieving the transcriptome corresponding to a given textual annotation and vice versa (a standard metric of CLIP model performance^9^) observing a mean area under the receiver operating characteristic curve (AUROC) value of 0.927 (Extended Data Fig. 1b). The trained CellWhisperer embedding model can be prompted with free-text queries to find matching transcriptomes. The query is processed with the BioBERT-based language model and the resulting embedding is compared to transcriptome embeddings from the Geneformer-based model. The result is a quantitative measure (the ‘CellWhisperer score’) that assesses the match between the query and each transcriptome in the examined dataset. A high CellWhisperer score indicates that a transcriptome constitutes a good fit for the free-text query.

Third, to enable natural-language chats that take the transcriptome information into account, we customized and fine-tuned the Mistral 7B open-weights LLM^13^ to incorporate CellWhisperer transcriptome embeddings in addition to text queries. Our approach is inspired by multimodal LLMs that can interpret and converse about images, such as GPT-4, Gemini and LLaVA^14^. We generated a training dataset of 106,610 conversations including simple rule-based question–answer pairs (for example, ‘What does the sample represent?’, with the sample’s textual annotation as the designated answer) and more complex LLM-generated conversations about transcriptomes and cells (technical details are provided in the Methods, examples in Supplementary Note 2). We used the embeddings together with the training-set questions as input to the Mistral 7B LLM (with an adapter layer that converts the embeddings into Mistral-compatible token-level embeddings) and fine-tuned this LLM to produce the matched answers. The resulting fine-tuned LLM responds to free-text questions and engages in natural-language chats about cells and their biological functions, gene-regulatory mechanisms and other biological processes that can be linked to transcriptional cell states.

To illustrate CellWhisperer’s ability to process, organize and annotate large transcriptome datasets, we clustered the CellWhisperer embeddings for 705,430 GEO-derived human transcriptomes and used the CellWhisperer chat model to textually annotate these clusters (Fig. 1b; interactive version at https://cellwhisperer.bocklab.org/geo). The CellWhisperer embeddings successfully captured cell types, developmental stages, tissues, diseases and other cell characteristics. For example, when querying the embedding model with the search term ‘infection’ and projecting the CellWhisperer score (which quantifies the match between the query and each transcriptome) on the UMAP (uniform manifold approximation and projection) visualization of transcriptomes from GEO, it highlights clusters of cells involved in the immune response to infections (Fig. 1c). As each data point in this UMAP connects back to a sample in the GEO database, we can retrieve the corresponding metadata and for example assess the popularity of RNA-seq analysis for certain cell clusters and biological functions over the last decade (Fig. 1d).

In summary, we built a multimodal AI that facilitates the seamless transition from transcriptomes to text and vice versa and enables the chat-based analysis of bulk and scRNA-seq data in English language.

### CellWhisperer predicts diverse cell characteristics

**Fig. 2:**
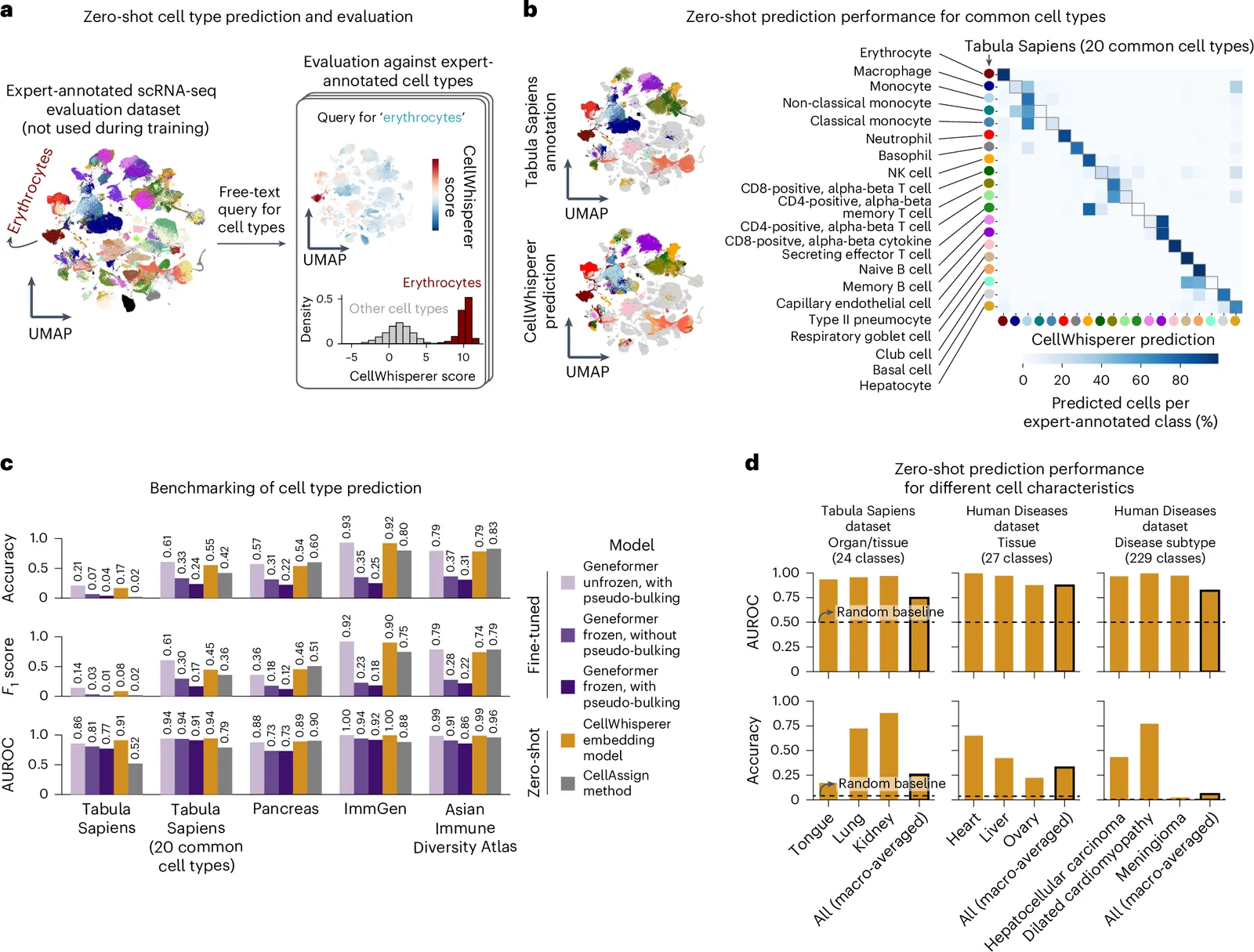
Benchmarking of the CellWhisperer embedding model through zero-shot prediction of cell characteristics. **a**, Conceptual outline of the performance evaluation for zero-shot prediction of cell types in a zero-shot manner. Left, UMAP visualization of CellWhisperer embeddings for all cells in the Tabula Sapiens dataset, colored by the dataset’s expert-annotated cell types (as a ground truth). Right, CellWhisperer scores for the free-text query ‘erythrocytes’ projected on the UMAP (top) and as histograms for expert-annotated erythrocytes versus other cell types (bottom). **b**, Comparison of expert-annotated cell types (UMAP, top left) and CellWhisperer predictions (UMAP, bottom left) for the Tabula Sapiens (20 common cell types) dataset, with the confusion matrix shown as a heat map (right). The blue color gradient indicates the percentage of cells of a given type (rows: expert-annotated ground truth) that are predicted as the cell type indicated by the columns. **c**, Bar plots of cell type prediction performance across multiple datasets and prediction methods. CellWhisperer’s zero-shot prediction performance (orange) is compared to the Geneformer scFM fine-tuned for cell type prediction (purple) and with the CellAssign method for marker-based cell type prediction (gray). The plots show values that were macro-averaged across classes. **d**, Bar plots of CellWhisperer’s zero-shot performance for predicting different cell characteristics (organ, tissue and disease), shown for selected individual classes and as macro-averages across all classes in the corresponding dataset. Dotted black lines denote random baseline performance (AUROC: 0.5; accuracy: 1/number of classes).

To assess how well the multimodal CellWhisperer embedding model has learned relevant aspects of human biology, we tested its ability to predict cell characteristics such as cell types, diseases, tissues and organs on the basis of cell transcriptomes in a zero-shot manner (that is, without task-specific fine-tuning or reference data). To that end, we selected expert-annotated transcriptome datasets that were not included in CellWhisperer’s training data and we used CellWhisperer to assign scores for each potential cell type label to each transcriptome (Fig. 2a). We then calculated the coherence between the correct cell type labels (as annotated in the dataset) and the computed CellWhisperer scores to quantify CellWhisperer’s ability to correctly annotate and identify cells and transcriptomes. We provide detailed evaluation results for this analysis in Supplementary Table 1.

In the Tabula Sapiens dataset, which comprises scRNA-seq profiles for 483,152 cells from 24 organs^19^, CellWhisperer distinguished 20 common cell types with an AUROC value of 0.94 (Fig. 2b,c). Mix-ups were mainly between closely related cell types, such as ‘monocytes’ versus ‘classical monocytes’ and between subgroups of T cells (Fig. 2b). Across all 177 annotated cell types, we obtained an AUROC value of 0.91, but with a lower accuracy value given many highly similar cell types (Fig. 2c). For bulk RNA-seq profiles of immune cells from the ImmGen consortium^20^ (GSE227743) and for a recently published scRNA-seq dataset of immune cells from Asian individuals^21^, we obtained AUROC values above 0.99; for a challenging scRNA-seq meta-analysis of human pancreas with closely related cell types and pronounced batch effects^22^, the AUROC value was 0.89 (Fig. 2c). These results support the robustness of our model.

**Extended Data Fig. 2:**
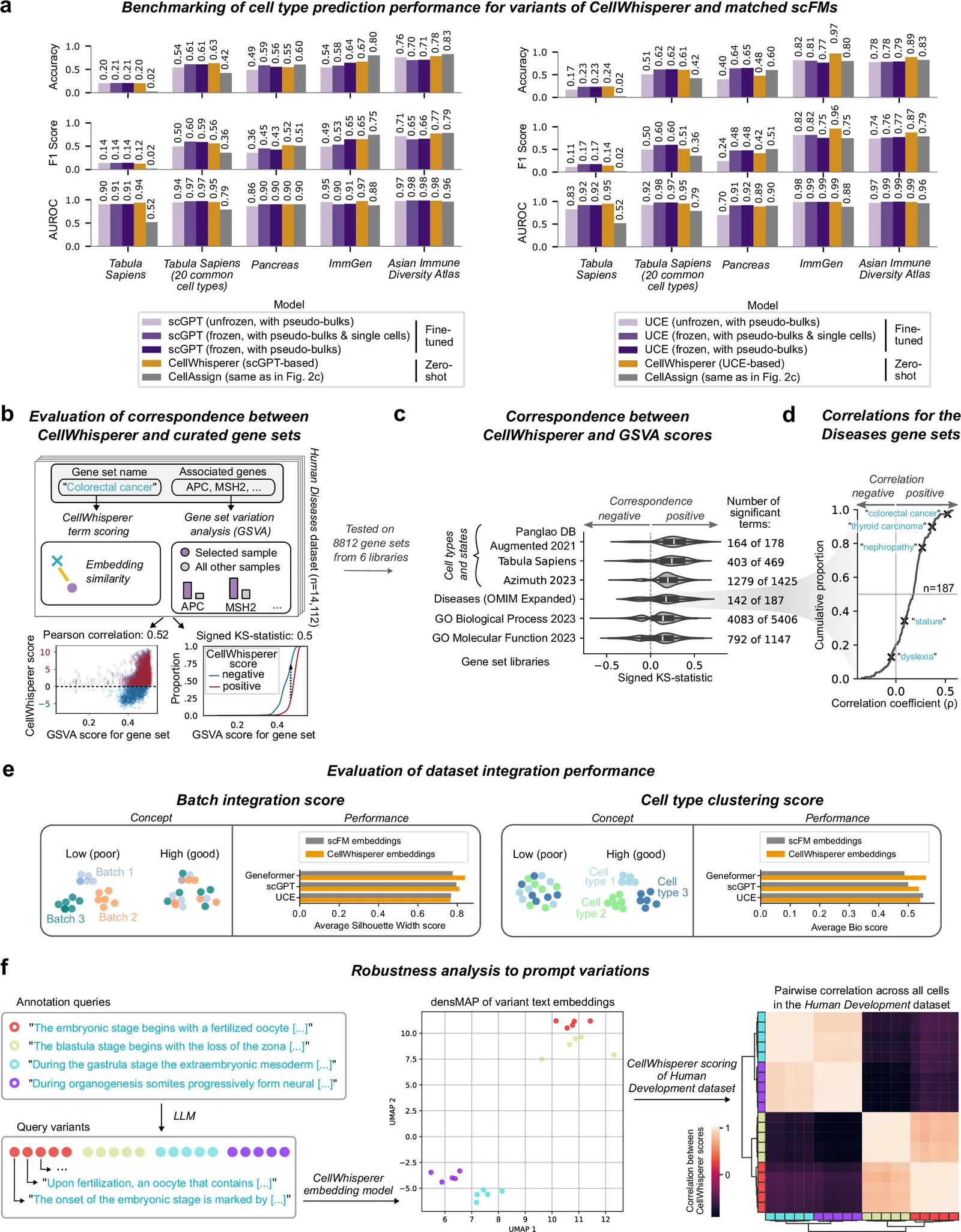
Additional evaluations of the CellWhisperer embedding model. **a**) Cell type prediction performance across multiple datasets for two variants of the CellWhisperer embedding model and their matched scFMs fine-tuned for cell type prediction (left: scGPT; right: UCE; same panel format as in Fig. 2c). **b**) CellWhisperer evaluation based on gene sets, comparing CellWhisperer scores computed for gene set labels to the enrichment or depletion of the corresponding genes, calculated across the Human Diseases dataset. Plots at the bottom show the relation between the two metrics for the gene set “colorectal cancer” from the Diseases (OMIM_Expanded) gene set library. KS: Kolmogorov-Smirnoff, GSVA: gene set variation analysis. **c**) Correspondence (x-axis, measured by the signed KS statistic as in panel **b**) between the CellWhisperer score and the GSVA score, averaged across all gene sets in each of six gene set libraries (y-axis). The number of gene sets for which the association is positive and statistically significant (p < 0.05) is indicated on the right. Violin plots are shown with inner boxplots corresponding to the interquartile range and whiskers extending to the farthest data point within 1.5 times the interquartile range. **d**) Distribution of Pearson correlation coefficients between the CellWhisperer score and the GSVA score for the Diseases (OMIM_Expanded) gene set library across all samples in the Human Diseases dataset. Selected gene set labels are shown for illustration. **e**) Conceptual outline and results of the CellWhisperer performance evaluation based on batch effect correction (left) and cell type clustering (right), comparing three variants of the CellWhisperer embedding model (orange) to their matched scFMs. Scores were computed for the Tabula Sapiens dataset, assessing how well the technical variation was removed (batch integration score) and how well the biological differences between cell types were retained (cell type clustering score). The batch integration score corresponds to the average silhouette width; the cell type clustering score corresponds to the average bio score. **f**) Analysis of CellWhisperer embeddings and corresponding CellWhisperer scores for textual variations of four queries. Left: CellWhisperer queries applied to the Human Development dataset and overview of LLM-generated query variants. Center: densMAP visualization of CellWhisperer text embeddings for all query variants. Right: Pearson correlation of CellWhisperer scores on the Human Development dataset for pairs of all query variants. Row and column sorting of the heat map was determined by clustering of the correlation coefficients.

Although the CellWhisperer embedding model was never specifically trained to predict cell types (this capability emerged from the more general task of learning connections between transcriptomes and their textual annotations), its zero-shot predictions performed better than a widely used marker-based method^23^ and on par with three scFMs^17,24,25^ that were fine-tuned for cell type prediction (Fig. 2c and Extended Data Fig. 2a). We also assessed our use of Geneformer^17^ as the scFM in the CellWhisperer embedding model relative to two alternative scFMs (scGPT^24^ and UCE^25^) and we observed comparable performance trends (Extended Data Fig. 2a).

To test whether CellWhisperer can also predict other cell characteristics, we assessed its zero-shot prediction performance for sample annotations of diseases, tissues and organs. To that end, we assembled a collection of 14,112 disease-associated transcriptomes from GEO that were excluded from our training data. Predicting 229 disease subtypes represented in this Human Diseases dataset, CellWhisperer achieved an AUROC value of 0.82 (Fig. 2d), indicating that disease prediction is harder than cell type prediction but possible with a performance that is substantially better than a random baseline. Similarly, CellWhisperer was able to predict the tissue-of-origin of bulk and single-cell transcriptomes with better-than-random prediction performance both in the Tabula Sapiens dataset (AUROC: 0.75) and in the Human Diseases dataset (AUROC: 0.87) (Fig. 2d).

To gauge the breadth of biological processes captured by our model, we investigated its recognition of expert-curated gene sets spanning diverse areas of biology. For each of 8,812 gene sets, we used the gene set label (such as ‘colorectal cancer’) as a query text to CellWhisperer and determined how well each sample in our Human Diseases dataset matched the query. We then calculated the correlation between this purely text-based assessment (which does not use any information about which genes are part of the gene set) and the gene expression enrichment for the genes in the gene set, across all samples in the Human Diseases dataset (Extended Data Fig. 2b). In other words, we tested whether CellWhisperer had implicitly learned an understanding of the genes that matter for established biological concepts, represented here by gene sets and their labels. We found a clear positive association between CellWhisperer scores for these labels and the expression of their corresponding gene sets (Extended Data Fig. 2c,d and Supplementary Table 2), indicating that our model has learned (albeit imperfectly) many of the tested biological concepts. Importantly, CellWhisperer achieved this by training on transcriptomes and their textual annotations, without having seen any expert-curated gene sets during model training.

For further evaluation, we tested how well our model can distinguish between biological signal and technical noise in the Tabula Sapiens dataset, based on an established benchmark for dataset integration and batch effect correction^22^. We observed improved performance of the CellWhisperer multimodal embeddings compared to transcriptome-only scFMs, for both Geneformer and scGPT, whereas UCE did not profit from the multimodal CellWhisperer training (Extended Data Fig. 2e). The best overall performance was obtained for the standard version of CellWhisperer, which uses Geneformer for the transcriptome embedding.

Lastly, we assessed how well the CellWhisperer embedding model handles complex prompts and variations within them, based on a scRNA-seq dataset of human embryonic development (described in detail below). We systematically compared different wordings of the same queries and observed strong concordance between their CellWhisperer scores (Extended Data Fig. 2f). Nevertheless, CLIP-based models are known to be sensitive to prompt variations^9^ and we caution that different query wordings may result in different results.

In summary, multiple lines of evidence (including zero-shot prediction of cell types, diseases, tissues and organs, a data integration task, gene set prediction from their labels and evaluation of prompt variations) support our conclusion that the CellWhisperer embedding model has learned a meaningful representation of cell states and biological processes, based on training data of transcriptomes and matched textual annotations.

### CellWhisperer identifies marker genes of organ development

**Fig. 3:**
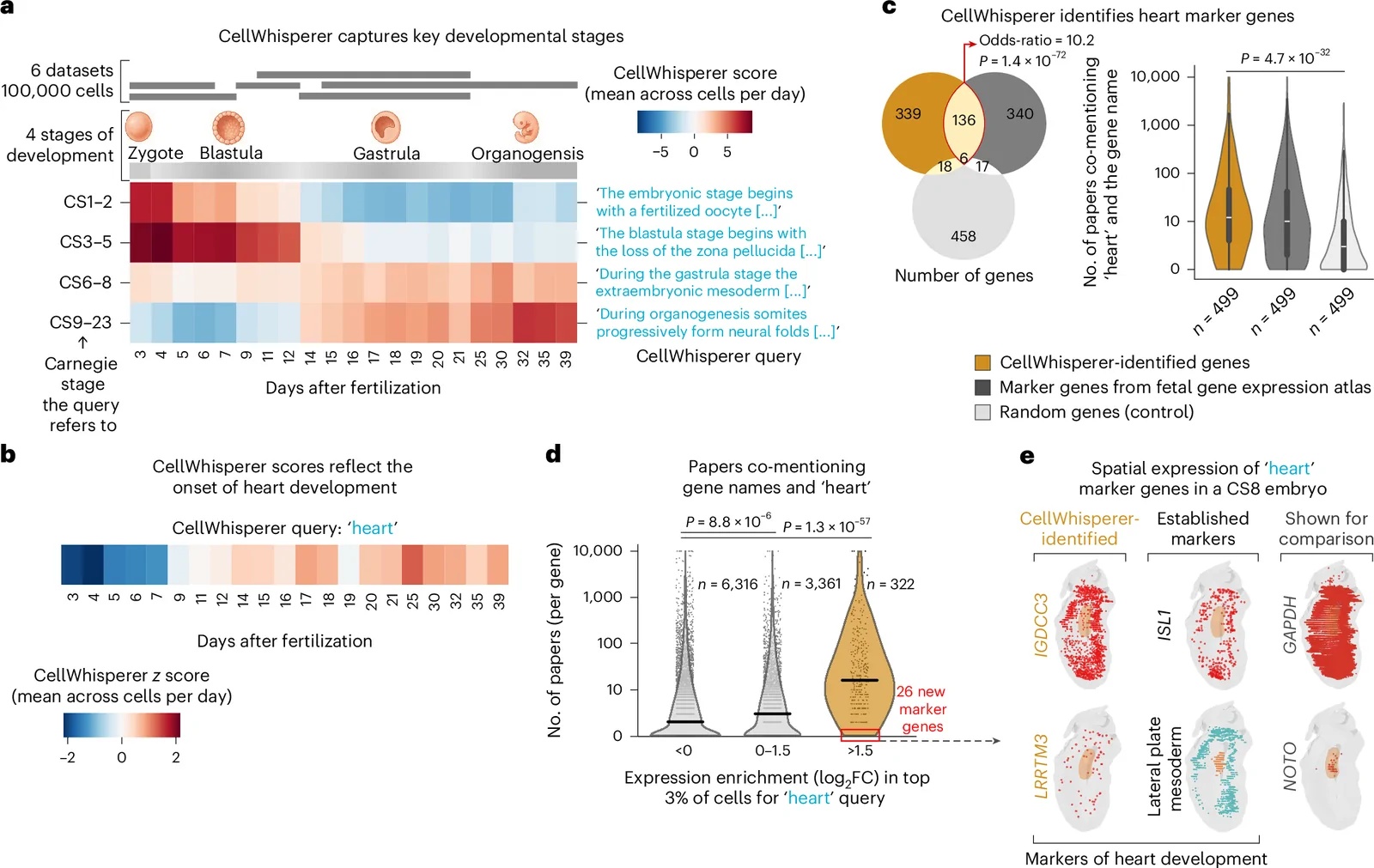
CellWhisperer analysis of organ development based on scRNA-seq datasets of human embryos. **a**, Overview of the Human Development scRNA-seq dataset with CellWhisperer scores for four key developmental stages. Queries were derived from Carnegie stage annotations using GPT-4o. The heat map shows mean CellWhisperer scores calculated across all cells for each time point. **b**, CellWhisperer scores for query ‘heart’. The average score across all cells was calculated for each time point and then standardized across time points (as *z* scores). **c**, Overlap between CellWhisperer-identified marker genes of heart development (brown), previously reported heart-specific markers derived from an atlas of fetal gene expression^32^ (dark gray) and randomly selected genes as controls (light gray). Left, Venn diagram with odds ratio and *P* value (two-sided Fisher’s exact test). Right, number of papers per gene that co-mentioned the gene name and the term ‘heart’ (two-sided Mann–Whitney *U*-test). For fair comparison, CellWhisperer-derived genes were selected to yield a matching number of genes. Inner box plots correspond to the interquartile range, with whiskers extending to the farthest data point within 1.5 times the interquartile range. **d**, Number of papers per gene that co-mentioned the gene name and the term ‘heart’, stratified by gene expression enrichment in CellWhisperer-identified heart cells (*x* axis). *P* values are based on two-sided Mann–Whitney *U*-tests. Genes with strongly enriched expression in CellWhisperer-identified heart cells (rightmost plot) but no associated papers (red box) were analyzed further. **e**, Spatial gene expression in a Carnegie stage 8 (CS8) human embryo for two CellWhisperer-identified marker genes of the developing heart (left); for an established heart marker gene (*ISL1*) and a gene set related to heart development (center); and for a widely expressed gene (*GAPDH*) and a notochord-specific gene (*NOTO*) shown for comparison (right). The notochord as a reference region is marked in orange and gene expression is denoted by colored points.

To illustrate CellWhisperer’s utility in a more complex biological application, we performed a meta-analysis of embryonic development on the basis of scRNA-seq data of human embryos that we curated from the literature^26–31^. We identified and integrated six separate datasets with 95,092 scRNA-seq profiles of human embryos collected 3–38 days after fertilization. These data, which were not part of our training dataset, were processed and annotated with CellWhisperer (Fig. 3a; https://cellwhisperer.bocklab.org/development).

To investigate whether CellWhisperer can identify temporal dynamics in embryonic development, we prepared queries corresponding to four key developmental stages using LLM-based aggregation of vertebrate embryology descriptions. The CellWhisperer scores for these queries matched the expected timing for these stages (Fig. 3a).

**Extended Data Fig. 3:**
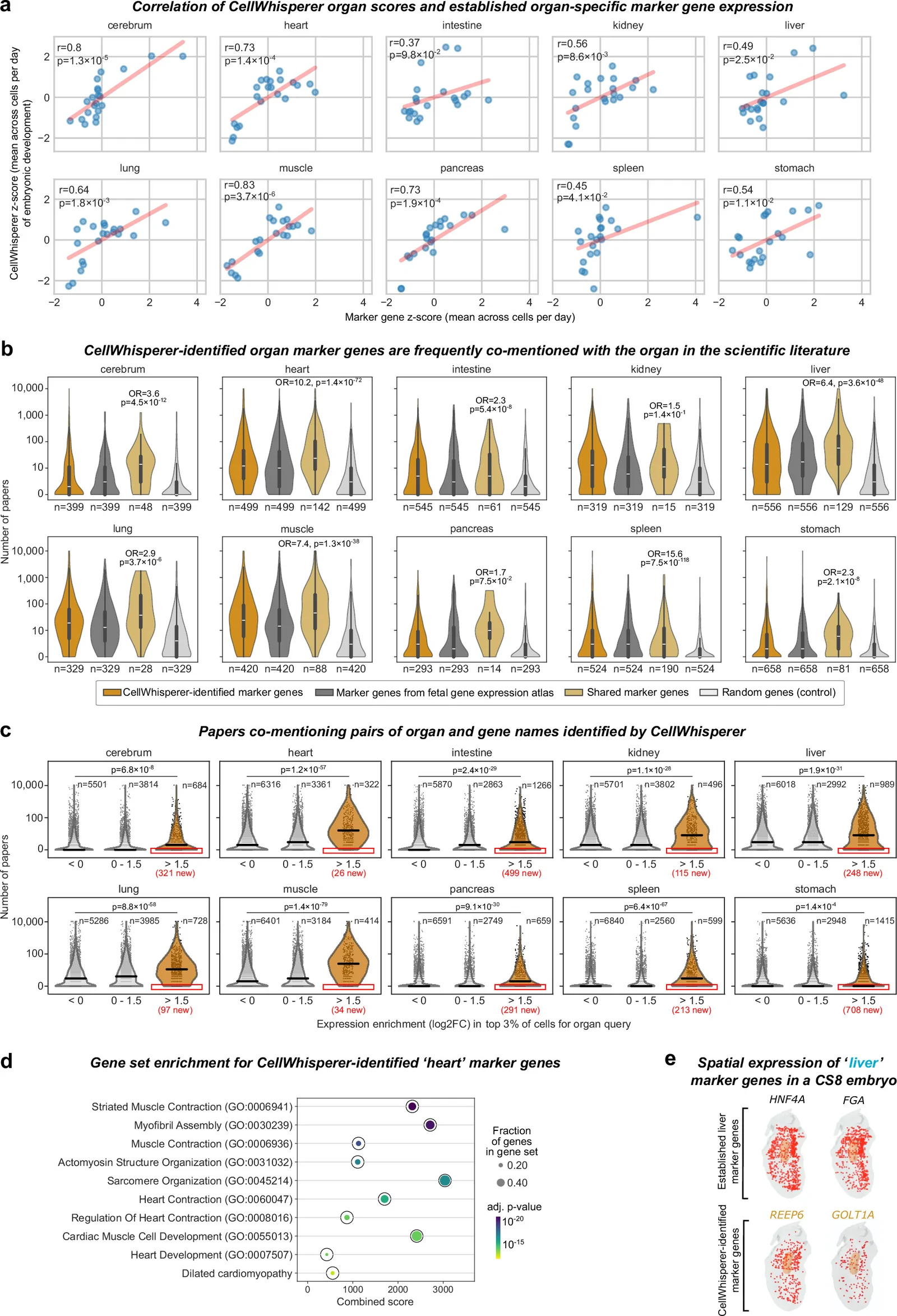
Additional CellWhisperer analyses of human organ development. **a**) Correlation between CellWhisperer scores (y-axis) and the expression of literature-reported organ marker genes (x-axis) for each organ across time points (dots). For both metrics, the mean across all cells at each time point was calculated and then standardized across all time points (as z-scores). Pearson correlation coefficients are reported together with their associated p-values (two-sided t-test for correlation significance). **b**) Number of papers per gene that co-mentioned the gene name and the organ name (as in Fig. 3c; results for heart are shown in both panels). Shared marker genes (light brown) indicate the overlap between CellWhisperer-identified (brown) and literature-reported (dark grey) organ marker genes, quantified by odds ratio and p-value (two-sided Fisher’s exact test). A size-matched set of random genes is plotted for comparison (light grey). Violin plots are shown with inner boxplots corresponding to the interquartile range and whiskers extending to the farthest data point within 1.5 times the interquartile range. **c**) Number of papers per gene that co-mention the gene name and the organ name (as in Fig. 3d; results for heart are shown in both panels), stratified by gene expression enrichment in CellWhisperer-identified organ-specific cells (x-axis). P-values correspond to two-sided Mann-Whitney U tests comparing CellWhisperer-identified marker genes (rightmost violin with a log_2_ fold-change above 1.5) and non-marker genes (leftmost violin with log_2_ fold-change below zero). Genes with strongly enriched expression in CellWhisperer-identified organ-specific cells but no associated papers are marked with red boxes. **d**) Gene set enrichment analysis for CellWhisperer-identified heart marker genes. The combined score (x-axis; c = log(p) × z) integrates the Fisher’s exact test p-value (two-sided) with the z-score of rank deviation to capture both statistical significance and effect size. **e**) Spatial gene expression in a Carnegie stage 8 (CS8) human embryo for two CellWhisperer-identified marker genes of the developing liver (bottom) and for two established liver marker genes (top). The notochord as a reference region is marked in orange, and gene expression is denoted by red points.

We next used a similar approach to identify phases of organ development, querying CellWhisperer with the names of ten organs (Extended Data Fig. 3a) as illustrated for ‘heart’ (Fig. 3b). These basic text queries implicitly captured a gradual activation of genes important for organ development, which we validated against the expression of organ-specific marker genes derived from an atlas of fetal gene expression^32^ (Extended Data Fig. 3a).

CellWhisperer embeddings are biologically interpretable not only through their link to descriptive text but also by examining genes associated with high CellWhisperer scores. We determined CellWhisperer-identified marker genes for each of the ten investigated organs (Supplementary Table 3) and indeed observed strong overlap with previously reported organ marker genes^32^ (median odds ratio: 3.3) (Fig. 3c and Extended Data Fig. 3b).

For further validation, we investigated how frequently the CellWhisperer-specific marker genes were co-mentioned with the corresponding organ in publications from the PubMed database of biomedical literature. We found that these genes were co-mentioned with the organ much more frequently than a random set of genes and comparably often as the previously reported organ marker genes^32^ Genes that were shared between both analyses had the highest frequency of co-mentioning (Extended Data Fig. 3b).

For each organ, the CellWhisperer analysis identified at least ten new marker genes beyond the previously reported organ marker genes^32^ (Supplementary Table 3). These genes had strong support from our analysis of co-mentioning in the biomedical literature (Fig. 3d and Extended Data Fig. 3c). In addition, we observed gene set enrichments for biological functions that are characteristic for the corresponding organs (shown for heart in Extended Data Fig. 3d) and a strong spatial expression correspondence with established and widely used organ marker genes, as validated using a 3D atlas of a gastrulating human embryo^33^ (Fig. 3e).

In summary, we applied CellWhisperer to the common and nontrivial task of marker gene discovery across multiple user-provided scRNA-seq datasets, which was achieved using simple text queries (comprising only the organ name) and yielded results that complement previously reported organ marker genes^32^ at comparable precision.

### Chat-based analysis of scRNA-seq data with a web interface

**Fig. 4:**
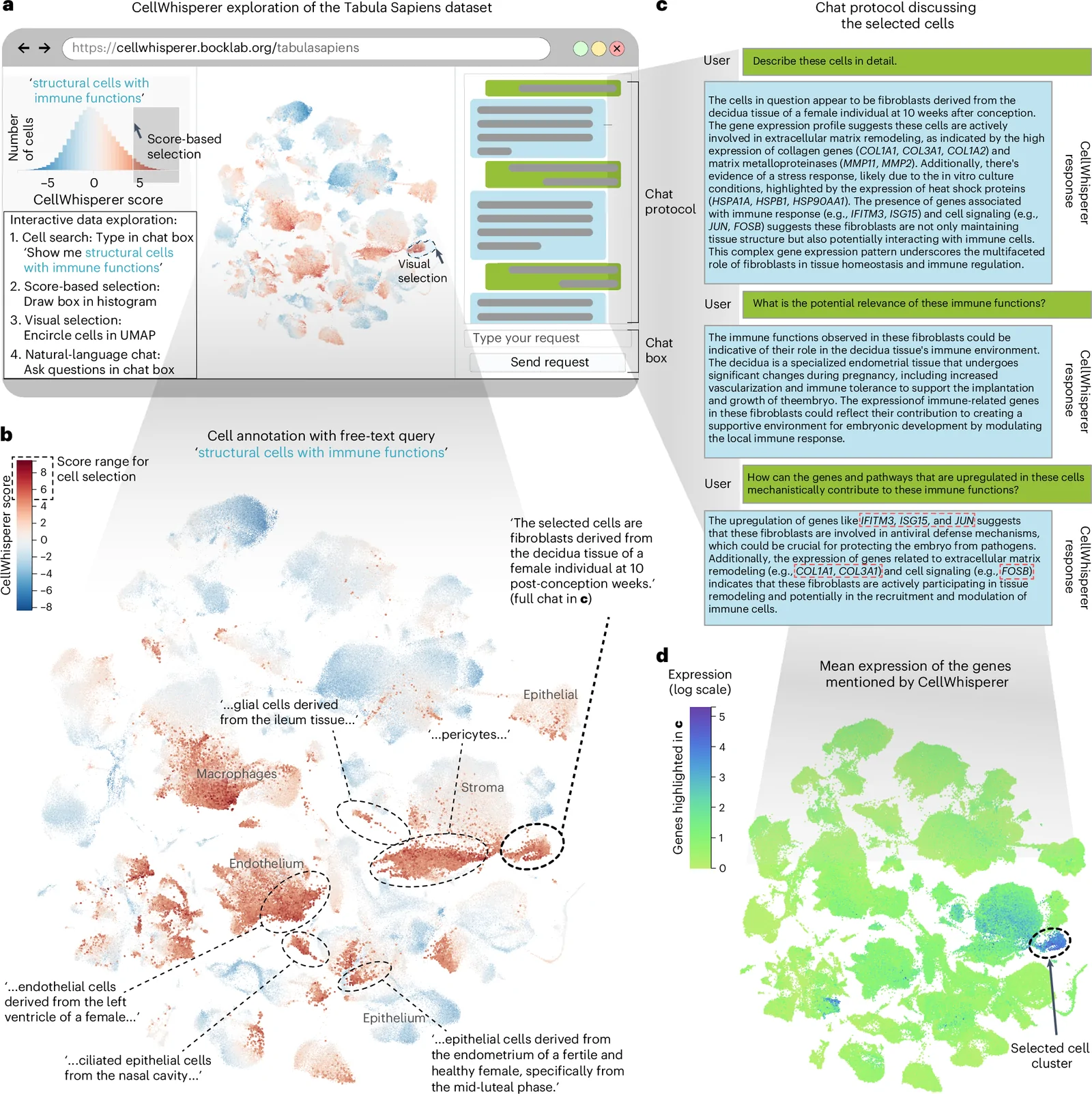
Interactive chat-based exploration of scRNA-seq data with CellWhisperer. **a**, Schematized screenshot of the CellWhisperer web tool, showing the Tabula Sapiens dataset with CellWhisperer scores for the free-text query ‘Show me structural cells with immune functions’. **b**, Zoomed-in view of the UMAP of CellWhisperer embeddings with overlaid CellWhisperer scores for the free-text query from **a**. Clusters of cells with high CellWhisperer scores were interactively selected in the web tool and examined by prompting the CellWhisperer chat model for a natural-language description (chat request: ‘Describe the selected cells’). Responses were trimmed to the most relevant parts (as indicated by ellipses). Annotations in gray font were manually added. **c**, Screenshot of a CellWhisperer conversation about the interactively selected cells (marked in **b**). **d**, Mean expression of the genes mentioned in the CellWhisperer responses in **c** (*IFITM3*, *ISG15*, *JUN*, *COL1A1*, *COL3A1* and *FOSB*) projected on the Tabula Sapiens dataset using the ‘gene sets’ feature of CELLxGene Explorer.

To make CellWhisperer broadly accessible for chat-based analysis of transcriptome data, we integrated it with the CELLxGENE Explorer by adding a CellWhisperer-powered chat box (Fig. 4a; https://cellwhisperer.bocklab.org). CELLxGENE Explorer is an interactive web tool for analyzing scRNA-seq profiles through visual inspection, filtering and differential analysis of cells and samples. CellWhisperer complements CELLxGENE Explorer’s functionality for visual analysis by providing natural-language data exploration capabilities including (1) free-text search for cells with user-specified properties; (2) automatic textual annotation of cell clusters; and (3) chat-based investigation of interactively selected cells. More generally, CellWhisperer enables the discussion of cells and genes in natural language through a chat box integrated with the visual features of a single-cell browser. We provide a list of usage examples in Supplementary Note 1.

Here, we illustrate CellWhisperer’s functionality on the Tabula Sapiens dataset of human organs^19^ (Fig. 4). In previous work, we described widespread immune gene activity in nonhematopoietic, structural cells of the mouse^34^, prompting us to explore this phenomenon in a large multi-organ human scRNA-seq dataset. We, thus, entered ‘structural cells with immune functions’ into the CellWhisperer chat box and obtained the corresponding CellWhisperer score as a color-coded overlay to the UMAP visualization of the Tabula Sapiens dataset (Fig. 4a,b). Among the cells that scored highly for this query were endothelial and epithelial cells, fibroblasts and pericytes (Fig. 4b), which are all known or suspected to have important immune-regulatory roles^35–37^.

To investigate these cells in more detail, we sequentially selected cell clusters with high CellWhisperer scores (by drawing a circle around the cells of interest) and prompted CellWhisperer by entering ‘Describe these cells in detail’ into the chat box (Fig. 4a–c). For each cell cluster, we obtained textual descriptions that were generated by the CellWhisperer chat model on the basis of the CellWhisperer transcriptome embeddings averaged across the selected cells (Fig. 4b). The resulting descriptions contained information about cell types, organs and developmental stages and, less frequently, details about potential sample donors (such as male or female), highly expressed genes (such as genes encoding collagens and matrix metalloproteinases in fibroblasts), biological functions (such as stress response) and other annotations. We found that the generated descriptions frequently referred to potential immune functions of the selected cells, consistent with our initial search query.

To obtain additional information about these cells, we interactively selected one of the cell clusters and asked two follow-up questions: ‘What is the potential relevance of these immune functions?’ and ‘How can the genes and pathways that are upregulated in these cells mechanistically contribute to these immune functions?’. This resulted in a coherent conversation with CellWhisperer, providing further characterization with highlighted genes and biological functions that are relevant in the selected cells (Fig. 4c). As a plausibility check, we confirmed the expression of those genes by projecting them on the UMAP (Fig. 4d).

**Extended Data Fig. 4:**
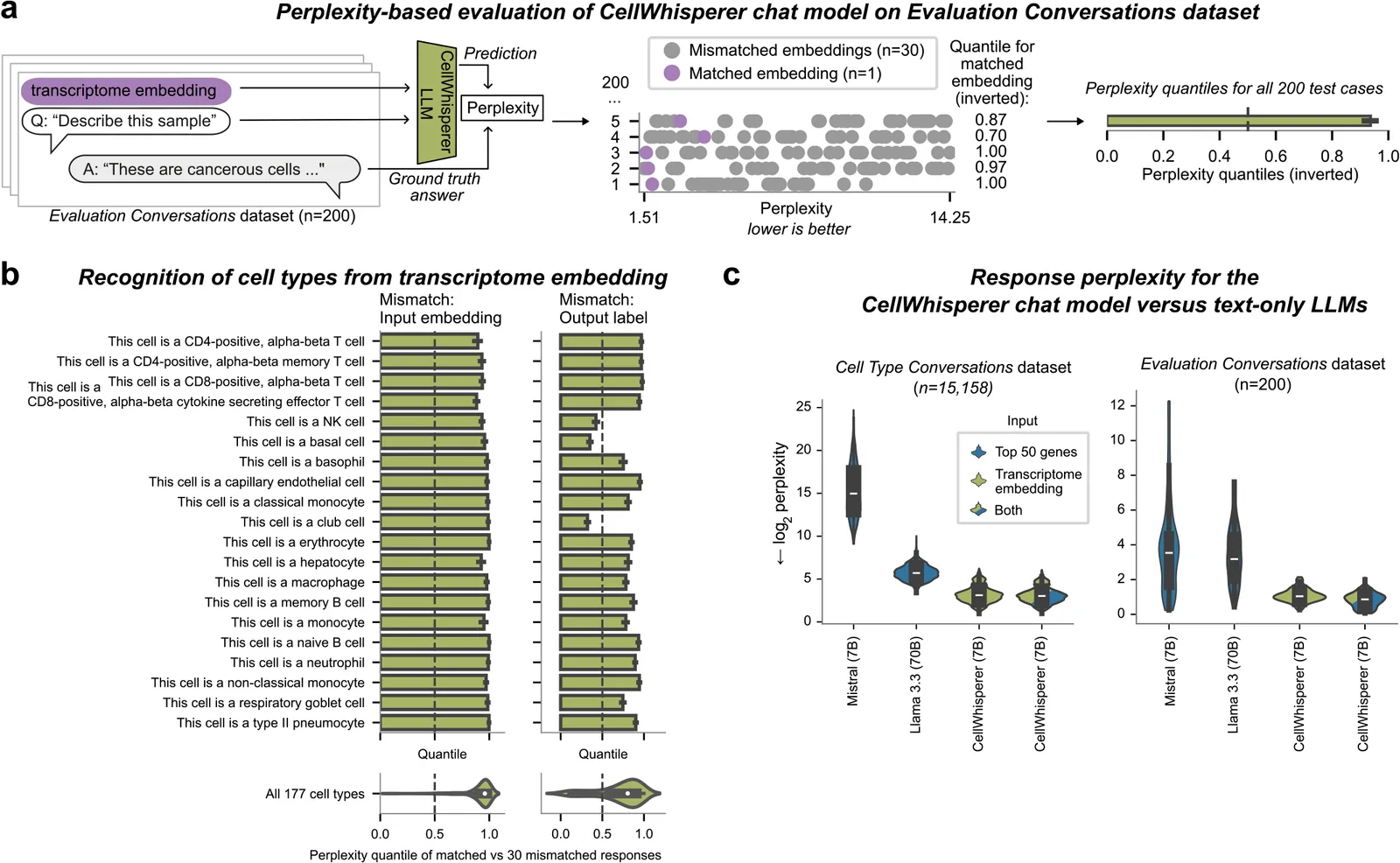
Evaluation of CellWhisperer text generation and chat performance. **a**) Left: Conceptual outline of the perplexity-based evaluation of the CellWhisperer chat model across 200 question-answer pairs from the Evaluation Conversations dataset with matched versus mismatched transcriptome embeddings. Center: Perplexity values for five question-answer pairs with one matched and 30 mismatched embeddings. Right: Inverted quantiles of perplexity values for the one matched transcriptome embedding relative to the perplexity values for the 30 mismatched embeddings. The barplot shows the mean of the (inverted) quantiles across all 200 question-answer pairs. Higher values indicate better performance; the dashed line at 0.5 indicates the random baseline. **b**) Perplexity-based performance evaluation of cell type prediction by the CellWhisperer chat model in the Cell Type Conversations dataset. Quantiles were computed by comparing the perplexity for the matched transcriptome against 30 mismatched transcriptomes (left) or for the matched cell type label against all 176 mismatched cell type responses (right) for each transcriptome. Bars indicate the mean across all data points; error bars correspond to the 95% confidence interval. Violin plots are shown with inner boxplots corresponding to the interquartile range and whiskers extending to the farthest data point within 1.5 times the interquartile range. **c**) Perplexity values for conversation responses using text-only LLMs (Mistral, Llama 3.3) and the CellWhisperer chat model. The transcriptome information was provided to the models as indicated by the color legend, with the rightmost violin representing the combination of both input types. Violin plots are shown with inner boxplots corresponding to the interquartile range and whiskers extending to the farthest data point within 1.5 times the interquartile range.

Lastly, we benchmarked the CellWhisperer chat model using the perplexity metric^38^, which is a common evaluation criterion for LLMs. We assessed how well each question-answer pair fits with the matched transcriptome in two test sets of biologically meaningful conversations (Methods). In our Evaluation Conversations dataset with 200 question-answer pairs, we observed a 90% preference for matched over unmatched transcriptomes (Extended Data Fig. 4a), which confirms that our LLM meaningfully interpreted the transcriptome embedding for its response generation. Furthermore, in the Cell Type Conversations dataset, we found that most cell type labels showed a preferential association with their matched transcriptomes (Extended Data Fig. 4b).

We further assessed the perplexity for responses obtained with the Mistral 7B LLM (which the CellWhisperer chat model builds upon) and for the much larger Llama 3.3 70B LLM (Extended Data Fig. 4c). CellWhisperer achieved best results (lowest perplexity values), even on the out-of-distribution Cell Type Conversations dataset, further supporting that our chat model effectively incorporates the CellWhisperer transcriptome embeddings. We also assessed whether the CellWhisperer chat model may benefit from explicitly providing a list of highly expressed genes as part of the prompt (as commonly done when analyzing transcriptomes with text-only LLMs^39,40^), in addition to the transcriptome embedding. We observed a mild beneficial effect (Extended Data Fig. 4c) and implemented this hybrid approach in the CellWhisperer web tool.

In summary, the integration of a CellWhisperer chat box in the CELLxGENE Explorer software provides user-friendly access to CellWhisperer’s AI features and demonstrates the complementarity of visual inspection and natural-language chats for the interactive exploration of scRNA-seq data.

### Exploratory analysis of user-provided scRNA-seq data

**Fig. 5:**
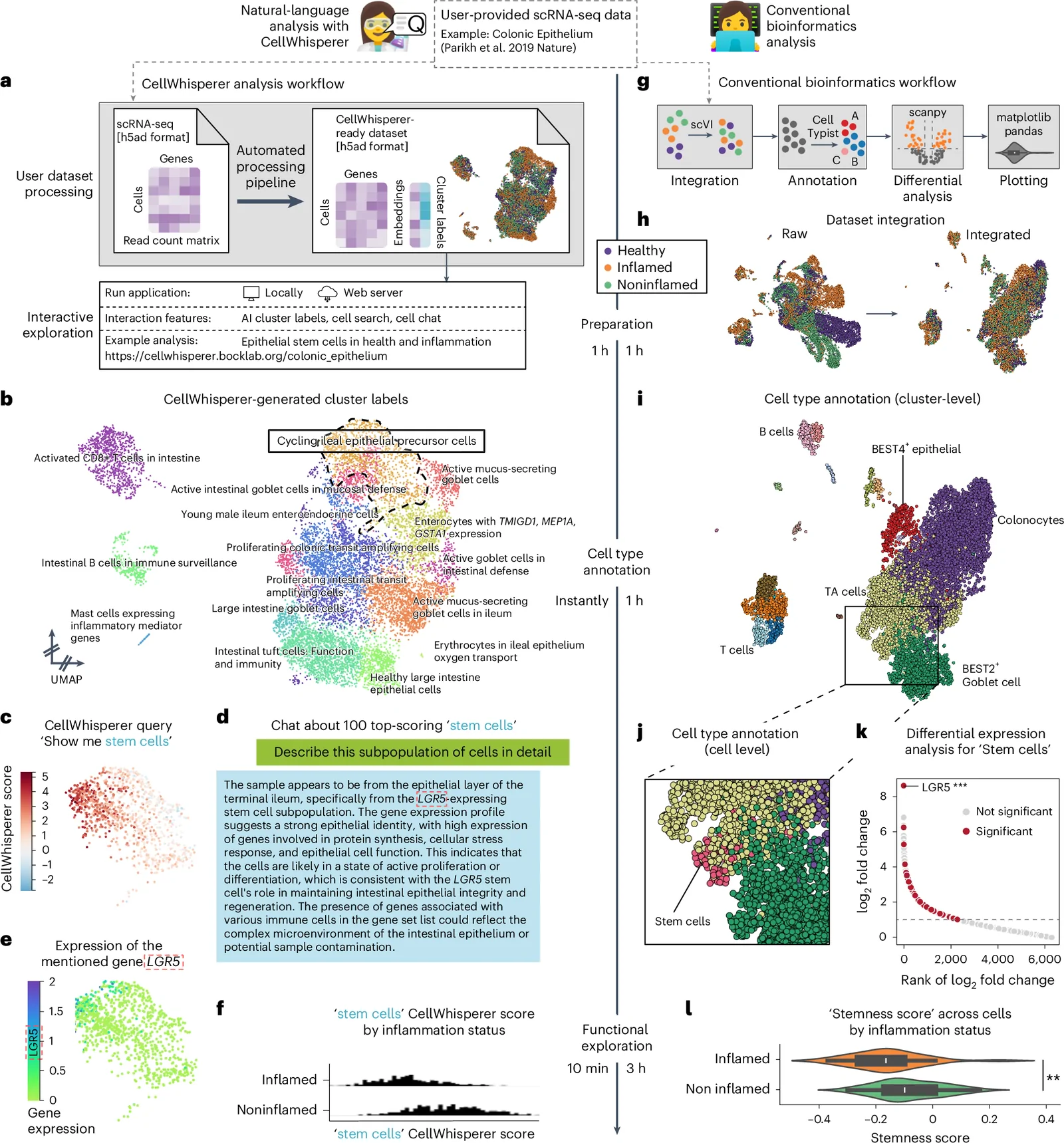
Interactive CellWhisperer-based and conventional bioinformatics analysis of a scRNA-seq dataset. **a**, Import and exploration of user-provided scRNA-seq data in CellWhisperer. **b**, UMAP of the CellWhisperer transcriptome embeddings for the imported Colonic Epithelium dataset^41^ comprising scRNA-seq profiles of inflamed and noninflamed tissue biopsies of individuals with inflammatory bowel disease and healthy individuals. Cluster labels were generated by CellWhisperer and clusters were repositioned for compact visualization (interactive version: https://cellwhisperer.bocklab.org/colonic_epithelium). **c**, Zoomed-in view of the cluster labeled ‘Cycling ileal epithelial precursor cells’, colored by CellWhisperer scores for the free-text query: ‘Show me stem cells’. **d**, CellWhisperer chat about the top 100 cells with highest CellWhisperer score (query from **c**). **e**, Expression levels of the *LGR5* gene mentioned in the CellWhisperer response (in **d**), plotted for the cell cluster from **c**. **f**, Histogram of CellWhisperer scores (query from **c**) for cells derived from inflamed versus noninflamed tissue. **g**, Outline of a conventional bioinformatics analysis that produces similar results as the interactive CellWhisperer analysis (**a**–**f**). **h**, UMAPs before and after batch effect correction using scVI. **i**, Cell type annotation using CellTypist with cluster-level majority voting. **j**, Identification of a cell subset labeled ‘Stem cells’ using CellTypist without cluster-level majority voting, plotted on top of the UMAP from **i**. **k**, Differentially expressed genes between putative stem cells (from **j**) and all other cells, ranked by log_2_-transformed fold change and colored by statistical significance (two-sided Wilcoxon test threshold: 0.0001) with a log_2_-transformed fold change of at least 1 (gray line). ***Adjusted *P* = 1.4 × 10^−25^. **l**, Differential expression of a generic stemness gene signature among the putative stem cells (from **j**) for cells from inflamed versus noninflamed tissue. Violin plots are shown, with inner box plots corresponding to the interquartile range and whiskers extending to the farthest data point within 1.5 times the interquartile range. **Adjusted *P* = 0.0024 (one-sided *t*-test).

To analyze user-provided transcriptome datasets with CellWhisperer, we developed a data-processing pipeline that computes CellWhisperer embeddings and annotations on the basis of the read count matrices from bulk RNA-seq or scRNA-seq (details are provided in the source code repository: https://github.com/epigen/cellwhisperer). The processed data are stored in a single file for dynamic loading into a user-hosted instance of CellWhisperer, while also facilitating reproducibility and sharing of CellWhisperer analyses. Here, we describe a typical CellWhisperer data analysis, investigating stem and progenitor cells in human colon and their response to inflammation (Fig. 5a–f); and we compare it to conventional bioinformatics analysis (Fig. 5g–l). Our analyses are based on scRNA-seq data of pathogenic and adjacent normal biopsies of persons with inflammatory bowel disease and healthy controls^41^.

The cluster labels generated by CellWhisperer (Fig. 5a) provide an initial overview of the dataset (Fig. 5b), identifying epithelial cells (‘Cycling ileal epithelial precursor cells’ and ‘Large intestine goblet Cells’) as well as immune cells (‘Activated CD8^+^ T cells in intestine’ and ‘Mast cells expressing inflammatory marker genes’). Among the ‘Cycling ileal epithelial precursor cells’, we searched for cells with stem cell characteristics using the CellWhisperer query ‘Show me stem cells’ and identified a subset of cells within this cluster that scored highly for this query (Fig. 5c). Further investigation of these putative stem cells in a follow-up conversation with CellWhisperer (Fig. 5d) suggested that this cell cluster includes *LGR5*-expressing epithelial stem cells, which constitute well-established stem cells of the gut^42^. As expected, *LGR5* gene expression (Fig. 5e) was highly correlated with the CellWhisperer score for the ‘Show me stem cells’ query (Fig. 5c).

We further compared the prevalence of the CellWhisperer-annotated epithelial stem cells between inflamed and noninflamed colon samples and we observed higher CellWhisperer scores for the ‘stem cells’ query among the noninflamed samples (Fig. 5f). These results suggest that chronic gut inflammation in persons with inflammatory bowel disease has a negative effect on *LGR5*-expressing epithelial stem cells, matching the conclusions of the study from which the dataset was obtained^41^ and previous in vitro experiments^43^.

Importantly, these analyses were performed swiftly and interactively with CellWhisperer. All figure panels (Fig. 5b–f) were taken from the web tool as screenshots (https://cellwhisperer.bocklab.org/colonic_epithelium).

For comparison, we sought to reproduce these results with a conventional bioinformatics analysis using custom Python code (Fig. 5g). We downloaded and preprocessed the gene expression profiles from GEO and visualized them as a UMAP (Fig. 5h, left). We observed substantial batch effects (which was less of an issue in the CellWhisperer analysis because the embedding model intrinsically adjusts for batch effects, as illustrated in Fig. 5a and Extended Data Fig. 2e); hence, we corrected for batch effects using the scVI method^44^ (Fig. 5h, right).

Next, we performed cell type annotation using the CellTypist software tool^45^. With CellTypist’s recommended parameters, no cell cluster was annotated as stem cells (Fig. 5i); however, when we reran CellTypist to predict the cell types of individual cells instead of cell clusters, we uncovered a subset of cells annotated as stem cells that were part of the broader cluster of transient-amplifying cells (Fig. 5j). These cells were characterized by high levels of *LGR5* expression (Fig. 5k), confirming that these are indeed epithelial stem cells. Lastly, we calculated a general ‘stemness score’ on the basis of a previously reported gene set^46^ and observed higher values in inflamed than in noninflamed colon samples (Fig. 5l), consistent with the CellWhisperer results.

This conventional bioinformatics analysis reproduced the conclusions of the interactive CellWhisperer analysis but it was much more complex and time-consuming. Overall, it took 400 lines of custom Python code, calls to five specialized software tools and the expertise of an experienced bioinformatician to plan and conduct the analysis.

In summary, CellWhisperer offers a rapid initial assessment of scRNA-seq datasets and an interactive approach to data exploration and hypothesis generation. In contrast, conventional bioinformatics analysis provides more fine-grained control and better traceability. Given the complementary strengths of these two approaches, we envision that chat-based analysis will guide rather than replace sophisticated code-based analyses.

## Discussion

Transcriptome profiling is widely used for characterizing biological states of cells and tissues, but data analysis and biological interpretation remain challenging. Here, we provide a proof of concept for scRNA-seq data exploration with natural language, using a multimodal AI model that combines transcriptome profiles with an understanding of biological text and an LLM-powered chat interface for interactive investigation of cell states.

Our performance evaluations and usage examples illustrate how multimodal models of transcriptomes and text facilitate exploratory analysis of biomedical data. CellWhisperer is most useful for exploratory analysis and for generating ideas and hypotheses in the early stages of data analysis, while key results should be reconfirmed with conventional bioinformatics approaches. We expect natural language to evolve into a widely used channel for interactive analysis of biomedical data, complementing visual data inspection and programming-based data analysis. We also envision natural language as a human-interpretable integration layer through which AI models of different scales (for example, FMs of molecules, cells, organs and individuals) will share and integrate their perspectives on a shared question, thereby facilitating multiscale and multimodal data analysis.

Methods such as CellWhisperer make data exploration more fluid, as users are unburdened by complex syntax and can interrogate biological knowledge within the interactive analysis. It also reduces barriers to entry, for example for biologists with no programming experience and strong preference for human language over computer code. Moreover, by connecting the chat functionality to voice recognition, it will be possible to interact verbally with the AI,for example in the context of virtual reality data analysis software or for researchers with vision impairment. Given the multi-language capabilities of many LLMs, it is technically feasible to support languages other than English. Data analysis in natural language may thus contribute to making bioinformatics more accessible, user-friendly and efficient.

CellWhisperer builds on recent advances in AI methodology. First, to establish our coherently annotated training dataset comprising a million bulk and pseudo-bulk transcriptomes, we used general-purpose LLMs for AI-assisted data curation of community-scale data repositories. Second, CellWhisperer uses powerful modality-specific embedding models to process transcriptomes (with Geneformer) and text (with BioBERT). Third, we adapted elements of the CLIP^9^ and LiT^47^ architectures for learning multimodal embeddings of transcriptomes and their textual annotations, which constitutes the foundation of the CellWhisperer model and software tool. Fourth, inspired by image-recognizing chat bots and other multimodal applications of LLMs^48^, we modified a general-purpose LLM to support chat-based analysis of scRNA-seq data by fine-tuning with 106,610 AI-generated transcriptome-centric conversations about cells and biological processes. Fifth, CellWhisperer follows the paradigm of FMs in the sense that it was trained once on large datasets covering a broad spectrum of biology and it handles diverse queries across biological domains without further training.

The current version of CellWhisperer constitutes a proof of concept that is useful for interactive exploration of scRNA-seq data, with some caveats. First, like other LLMs, CellWhisperer does not understand the user questions and its own responses in a human sense; rather, it has learned to continue the conversation on the basis of large amounts of training data on how transcriptome-centric question answering usually unfolds. We thus consider CellWhisperer a tool for exploratory analysis that should not be trusted blindly and without validation. Second, CellWhisperer relies on domain-specific models for its embedding of transcriptomes and text and on an LLM for text generation, thus inheriting their current limitations. To let CellWhisperer profit from progress with these models, we implemented a modular software architecture that makes it easy to swap the underlying models. Third, the CellWhisperer chat model occasionally ‘hallucinates’, most frequently by providing overly specific information about potential sample origins (such as ‘T cells from an 85-year-old male’). This behavior likely reflects the high abundance of such text in our training data, which could be addressed by fine-tuning with human feedback and/or data curation to remove spurious information from the training data. Fourth, CellWhisperer can only be as good as the available training data; hence, areas of biology that are not well represented in public databases are unlikely to be modeled well by CellWhisperer or similar models.

Considering concerns about the risks of modern AI^49^, we concluded that CellWhisperer can be considered of low risk, enabling us to make all aspects of the method and data openly accessible to the general public. We identified as the most relevant risk of CellWhisperer that incorrect answers may be left unchecked, thereby leading to wasted resources for validation experiments or, worse, the uncritical incorporation of falsehoods into scientific research. To mitigate this risk, we designate CellWhisperer as a tool for exploratory data analysis that should be used with a critical mind and we emphasize that key results should be validated with alternative methods (as illustrated in Fig. 5). In contrast, we did not identify any particular risks to humans or to the environment. Given the complexity and research-centric character of scRNA-seq profiling, it is highly unlikely that CellWhisperer results will be uncritically relied upon in clinical diagnostics and thereby harm persons. It has also been discussed whether AI tools facilitate the development of biological threats and bioweapons^50^. Given that CellWhisperer does not incorporate any biological data or knowledge that is not already in the public domain and does not provide any dedicated functionality for the design of chemicals, viruses or cells, we consider it highly unlikely that CellWhisperer could constitute a meaningful contribution to the toolbox of adversarial actors.

Since the initial description of CellWhisperer in a conference paper^51^ and bioRxiv preprint^52^, several methods have been released that share CellWhisperer’s ambition of making text-based approaches broadly useful for scRNA-seq data analysis. Most notably, LangCell^53^ uses a transcriptome–text contrastive learning approach similar to the CellWhisperer embedding model, C2S-Scale^54^ repurposes LLMs for cell-level interpretation by fine-tuning them on top expressed gene lists and BioDiscoveryAgent^55^ and BioChatter^56^ describe LLM-based agentic workflows for transcriptome data analysis.

While these studies underline the general interest in chat-based transcriptome analysis, several key elements continue to be unique to CellWhisperer. First, CellWhisperer enables the annotation of millions of single cells using a computationally efficient multimodal embedding model (in contrast, Cell2Sentence^54,57^ runs an expensive LLM for each single cell) and supports conversations about interactively selected cells (LangCell implements CLIP-style embedding but lacks the ability to chat about cells). Second, CellWhisperer was trained on a massive dataset from GEO and CELLxGENE Census curated with semantic LLM processing, covering diverse human biology from over 20,000 individual studies. In contrast, most related studies exclusively relied on CELLxGENE Census (which only covers hundreds of studies and about 1,000 different samples) and did not incorporate detailed textual metadata beyond aggregating predefined columns. Third, CellWhisperer implements a directly usable workflow and a web-based user interface that showcase a novel analysis paradigm for scRNA-seq analysis, with integration into the widely used CELLxGENE Explorer.

In conclusion, CellWhisperer establishes a user-friendly approach for exploring scRNA-seq data, driven by chat-based analysis with natural language. Our method uses AI models to emulate data-centric conversations between biologists and bioinformaticians. We anticipate that natural language will become a broadly useful channel for biological data analysis and it constitutes a key building block of future AI-based bioinformatics research assistants.

## Methods

### Multimodal training data of paired transcriptomes and text

To establish a large training dataset of transcriptomes and their matched textual annotations, we processed two community-scale repositories: GEO^10,11^ and CELLxGENE Census (version 2023-12-15)^12^.

From GEO we obtained RNA-seq count matrices of 722,425 human transcriptomes that were uniformly processed by the ARCHS4 project (version 2.2, 30 May 2023)^16^. We removed 7,049 samples with fewer than 250 expressed genes and 9,946 samples that overlapped with our Human Diseases dataset (described below), resulting in 705,430 transcriptomes. For each transcriptome, we obtained the associated metadata using the Entrez API with either the sample’s experiment accession, Bio-Sample accession or GEO accession (in this order of priority) and we removed binary data and special characters using the unidecode package. We included metadata fields describing study-level descriptions such as series design, growth protocol and series summary, as well as sample-level fields including sample title, treatment, and other fields that varied across studies.

From CELLxGENE Census we obtained scRNA-seq count matrices of 257 studies that were conducted on human samples using one of four assays with comparable data types (10x Genomics, Seq-Well, Drop-seq and CEL-seq2). We excluded cells with fewer than 100 expressed genes, resulting in a total of 19,663,838 scRNA-seq profiles. Within each sample, we grouped cells on the basis of their cell-level metadata, only considering metadata fields with fewer than 500 distinct values across all cells in the corresponding study, and we calculated pseudo-bulk transcriptomes by taking the mean of the scRNA-seq count values across all cells with identical metadata. Each of these 376,983 pseudo-bulk transcriptomes was linked to its cell-level metadata such as the cell type and to study-level metadata such as a study title and study abstract.

For each sample (from GEO) or pseudo-bulk transcriptome (from CELLxGENE Census), we generated a concise natural-language summary from the metadata using Mixtral 8x7b (Q5_K_M quantized version)^58^, using the llama.cpp Python bindings with a sampling temperature of 0.2, nucleus sampling (top_*p*) of 0.9 and top probability sampling (top_*k*) of 50. This LLM-based generation of concise textual annotations was guided by a prompt that we engineered on the basis of established practices such as pre-action reasoning^59^, role playing^60^ and few-shot learning^61^ with a manually curated set of examples. The prompt and illustrative examples of generated textual annotations are shown in Supplementary Note 2.

Data processing was performed on compute nodes with eight A100 80-GB GPUs. We estimate a total of 5,000 GPU hours for the LLM-assisted generation of textual annotations. The training dataset is available for download through the project website (https://cellwhisperer.bocklab.org).

### Multimodal design of the CellWhisperer embedding model

To enable transcriptome data analysis using natural language, we pursued a multimodal contrastive learning approach, with a neural network architecture that integrates matched pairs of transcriptomes and text into the same embedding space. Specifically, we adapted the CLIP method^9^, originally developed for joint multimodal embedding of images and text, and implemented it using pytorch^62^ with the lightning^63^ and the transformers^64^ libraries.

To account for the different properties of transcriptomes and natural-language text, CellWhisperer embeds the transcriptomes with Geneformer^17^ and the textual annotations with BioBERT^18^. The outputs of these two models are transformed into two 2,048-dimensional vectors using separate adapter modules, each consisting of two learnable linear layers connected by a rectified linear unit nonlinearity (ReLU) and followed by batch layer normalization. To enhance computational efficiency, we adopted the LiT approach^47^, initializing both models with pretrained weights and fine-tuning the text model and the adapter modules, while keeping the transcriptome model frozen.

The Geneformer model for transcriptome embedding uses 12 transformer encoder layers to process transcriptomes as ‘sentences of genes’ ranked by their expression; it was trained on ~30 million scRNA-seq profiles^17^. The BioBERT model for text embedding was trained on large biomedical text corpora^18^. We also tested alternative models (scGPT^24^ and UCE^25^ for transcriptome embedding; BioGPT^65^ for text embedding), which led to similar results (Supplementary Note 3).

### Training of the multimodal CellWhisperer embedding model

We trained the CellWhisperer embedding model on the 1,082,413 matched pairs of transcriptomes and textual annotations that we curated from GEO and CELLxGENE Census. For each pair, the transcriptome and the textual annotation were tokenized for processing with the two modality-specific transformer models, Geneformer and BioBERT. Specifically, the transcriptomes were sorted by gene expression levels and the top 2,048 most highly expressed genes were tokenized with a dictionary of human gene symbols^17^. The textual annotations were tokenized using WordPiece^18,66^ and trimmed to a maximum of 128 tokens for training efficiency (the vast majority of textual annotations were shorter and, thus, remained untrimmed).

We trained the multimodal embedding model with a mini-batch size of 512 and InfoNCE-based loss, which maximizes the cosine similarity between matched pairs of transcriptomes and textual annotations while minimizing the cosine similarity between all other (unmatched) pairs in a given training batch. Training was scheduled for 16 epochs at a maximum learning rate of 0.00001. For the first 3% of all training steps, we froze the Geneformer and BioBERT models to only train the embedding adapters and we linearly increased the learning rate from 0 to its maximum value (warmup). We then unfroze the BioBERT model and continued training with a second learning rate warmup for an additional 3% of the total number of training steps, followed by a learning rate cosine schedule over the remaining 94% of steps of the 16 epochs. The outputs of the consistently frozen Geneformer model were cached to decrease computational complexity during training.

Optimal hyperparameters, such as the maximum learning rate, were determined by stochastic grid search. As the performance metric for this optimization procedure, we tested the model’s ability to retrieve the correct textual annotation for a given transcriptome in our Human Diseases dataset. We used a deduplicated version of this dataset to increase the robustness of retrieval scoring, thereby reducing the impact of data points with very similar or identical textual annotations. We also used this metric to control for overfitting during model training. The corresponding validation scores of our final model are shown in Extended Data Fig. 1b.

A full training run (16 epochs) was completed in less than 24 h on an A100 GPU. The model checkpoints are available for download on the project website (https://cellwhisperer.bocklab.org). An ablation study providing a technical evaluation of the final model is described in Supplementary Note 3.

### Collection and curation of evaluation and demonstration data

To assess CellWhisperer’s performance and to demonstrate its functionality, we prepared the following bulk RNA-seq and scRNA-seq datasets and made sure to exclude them from all training data.

#### Human Diseases

We obtained 14,112 disease-annotated tissue samples from GEO, by querying the MetaSRA database for the terms ‘primary tissue’ and ‘disease state’, followed by manual curation based on metadata obtained from SRA, GEO and PubMed. To emulate a realistic application scenario with differences in the initial data processing, we processed these data with a different bioinformatics pipeline and a different LLM than what was used for the training dataset. Specifically, we used the fetchngs and rnaseq pipelines^67–69^ for preparing the transcriptome data, while the textual annotations used for retrieval analysis were prepared from metadata downloaded through the Entrez API (biopython) using GPT-4 through the OpenAI API with zero-shot prompting. Because this dataset contains many samples with identical or highly similar textual annotations, we also derived a Human Diseases (deduplicated) dataset for use in retrieval scoring. To that end, we processed all 14,112 textual annotations with BioBERT, performed hierarchical clustering on the embedding vectors (metric: cosine, linkage: average), retained the top 100 clusters and selected the transcriptome that was closest to the cluster center for each cluster, resulting in a total of 100 deduplicated transcriptomes with their nonredundant textual annotations.

#### Tabula Sapiens

From the Tabula Sapiens atlas of scRNA-seq profiles^19^, we obtained transcriptomes for 483,152 single cells across 15 individuals, 24 organs and 177 annotated cell types, with their cell_ontology_class annotation as cell type annotations (we standardized spelling and capitalization). Because of many infrequent cell types in the Tabula Sapiens dataset, we also derived a Tabula Sapiens (20 common cell types) dataset by retaining only the 20 most common cell types in liver, lung and blood (184,450 single cells).

#### ImmGen

From the RNA-seq profiles and manually curated cell types provided by the ImmGen consortium^20^ (GSE227743), we established a dataset comprising 42 bulk transcriptome profiles across five human immune cell types.

#### Asian Immune Diversity Atlas

From the Asian Immune Diversity Atlas^21^, we obtained scRNA-seq profiles for peripheral blood mononuclear cells from 619 healthy individuals spanning seven population groups in five countries across Asia. We randomly selected up to 1,000 cells per annotated cell type (including all cells if fewer than 1,000 were available), resulting in a total of 7,842 cells across nine cell types.

#### Human Development

We obtained scRNA-seq data of human embryonic development, spanning 3–38 days after fertilization (European Nucleotide Archive, ArrayExpress and GEO accessions: PRJEB30442 (ref. ^28^), E-MTAB-3929 (ref. ^26^), E-MTAB-8060 (ref. ^27^), PRJEB40781 (ref. ^29^), GSE232861 (ref. ^30^) and GSE155121 (ref. ^31^)). Raw sequencing data were uniformly processed and aligned to the human genome (assembly GRCh38) using Cell Ranger^70^ (E-MTAB-8060, GSE232861 and GSE155121) or using the rnaseq pipeline^67,69^ (all other datasets). The count matrices were corrected for batch effects with scANVI^71^, resulting in an integrated dataset of 95,092 scRNA-seq profiles.

#### Pancreas

We obtained a scRNA-seq meta-analysis dataset of human pancreas (https://figshare.com/ndownloader/files/43480497) comprising 16,382 scRNA-seq profiles. This dataset was previously assembled from individual studies that used different transcriptome profiling technologies and was used for benchmarking of methods for single-cell data processing and dataset integration^22^.

#### Colonic Epithelium

To showcase CellWhisperer’s analysis of user-provided datasets, we obtained scRNA-seq data for epithelial and other cells from the colon, starting from a normalized read count matrix retrieved from GEO (GSE116222). The dataset consists of 11,175 cells from three healthy individuals and three patients with inflammatory bowel disease. For these patients, samples were taken from inflamed and noninflamed regions.

### Evaluation of the CellWhisperer embedding model

We evaluated the trained multimodal embedding model in four complementary ways. First, we assessed CellWhisperer’s capability to predict cell characteristics such as cell types, tissues, organs and diseases in a zero-shot manner, by comparing CellWhisperer’s transcriptome embeddings to the text embeddings of the corresponding metadata-provided cell characteristic from the evaluation datasets. To that end, we embedded the transcriptomes and the corresponding cell characteristics (as natural-language statements, for example in the form ‘A sample of <cell type> from a healthy individual’) for each of the evaluation datasets. For every combination of a transcriptome and a text label, we quantified the agreement using the dot product, which constitutes the ‘CellWhisperer score’. We softmax-transformed the resulting scores for each given transcriptome to obtain probabilities across all labels in the dataset, and we calculated the mean of AUROC scores for these labels as a metric for the model’s zero-shot prediction performance.

Second, we evaluated how well the CellWhisperer embeddings capture biological (rather than technical) differences between cells and compared this to scFMs (Geneformer, scGPT and UCE). To that end, we embedded the Tabula Sapiens transcriptomes using either the CellWhisperer embedding model or any of the three scFMs and then compared batch effect correction and cell type clustering for the embeddings following a previously described workflow^72^. For cell type clustering, we used the average bio score^24^, which is the arithmetic mean of three metrics: average silhouette width^22^, normalized mutual information and adjusted rank index. For batch effect correction, we used a variant of average silhouette width as implemented in the silhouette_batch function^22^.

Third, we leveraged the broad catalog of biological phenomena provided by gene set libraries to assess which aspects of molecular biology were implicitly learnt by the CellWhisperer embedding model. To that end, we obtained 8,812 gene sets from gene set libraries of cell types^19,73,74^, diseases^75^ and Gene Ontology (GO) terms^76^ and we performed Gene Set Variation Analysis (GSVA) using the GSVA package^77^ with the ssgsea enrichment function^78^. GSVA supports gene set enrichment analysis based on read count matrices, providing quantitative results for individual samples against a background of unrelated samples. We ran GSVA across all 8,812 gene sets on the Human Diseases dataset, resulting in an 8,812-dimensional vector of gene set enrichments for each transcriptome. For the same transcriptomes, we also obtained CellWhisperer scores by embedding the names of the gene sets (such as ‘colorectal cancer’ from the OMIM_Extended disease library or ‘response to type I interferon’ corresponding to GO:0071357 from GO_Biological_Process_2023). For each gene set, we compared the GSVA and CellWhisperer scores across the transcriptomes in the dataset using the Pearson correlation and Kolmogorov–Smirnoff statistic (the latter was included for its robustness to nonlinear data).

Fourth, we evaluated complex queries and their variations using the Human Development dataset. To that end, we prepared queries for four key embryonic developmental stages (zygote, blastula, gastrula and organogenesis) and compared CellWhisperer scores for variations of these queries. We established the queries on the basis of the 23 Carnegie stages obtained from the Human Developmental Stages ontology (https://bioportal.bioontology.org/ontologies/HSAPDV), which we grouped into zygote (stages 1–2), blastula (stages 3–5), gastrula (stages 6–8) and organogenesis (stages 9–23). We condensed their annotations using GPT-4o with the following prompt: ‘Please, summarize the following sentences in just two sentences (max 500 characters)’. We visualized CellWhisperer scores for the four queries in a time-resolved manner (Fig. 3a). Moreover, to assess CellWhisperer’s robustness to query variations (Extended Data Fig. 2f), we generated five variants per query using GPT-4o with the following prompt: ‘Rewrite the provided text in five different variants. The length of the variants may vary slightly, but make sure that the semantics (i.e. the meaning of the generated variant text) remains close to the initially provided text. Return the variants as a JSON-formatted list of strings (key = “variants”)’. We then compared the CellWhisperer scores in the Human Development dataset between the query variants.

### Benchmarking of CellWhisperer’s zero-shot cell type prediction performance against alternative methods

We benchmarked CellWhisperer’s performance in zero-shot cell type prediction against a widely used marker-based method and against scFMs that we fine-tuned for cell type prediction.

Marker-based prediction was performed with CellAssign^23^ and the CellMarker 2.0 cell type marker database^79^, enabling reference-free cell type prediction akin to CellWhisperer’s zero-shot predictions. To resolve cell type naming mismatches between the CellMarker 2.0 database and our evaluation datasets, we mapped them using GPT-4o with the following prompt applied to each cell type in each evaluation dataset: ‘Assign the cell type <cell_type> to one of the following candidates: <candidates>. <newline> Only print the name of a single cell type, nothing else’. We only included cell types from the marker databases that matched the tissue(s) of the respective evaluation dataset and we excluded 66 marker genes from the CellMarker 2.0 database that were originally derived based on scRNA-seq data included in our Pancreas evaluation dataset.

For scFM-based cell type predictions, we fine-tuned three scFMs (Geneformer, scGPT and UCE) using 376,983 pseudo-bulk transcriptomes from CELLxGENE Census. To optimize performance, we tested three configurations for each scFM: (1) freeze the scFM and train only its classification head; (2) unfreeze the scFM and fine-tune the entire model; and (3) augment the pseudo-bulk data with single-cell data (at a 1:4 ratio) during training. We assessed the performance on our evaluation datasets by mapping the classifier outputs (class probabilities) to the cell types in each evaluation dataset using an LLM with the following prompt: ‘Assign the query cell type <cell type> to one of the following candidates: <candidates>. <newline> Only print the name of a single cell type, nothing else, and don’t just repeat the query cell type. Make sure to return one of the candidates’. We allowed multiple classifier outputs to be assigned to the same cell type class and summed up their predicted probabilities to avoid unfair penalization of the scFM classifiers, which predict a larger number of cell types.

### Identification of marker genes of human organ development using CellWhisperer

The CellWhisperer-identified marker genes for each organ were obtained by selecting the cells with the top 3% highest CellWhisperer scores for the corresponding organ-specific query (such as ‘heart’) and then identifying the differentially expressed genes (log_2_-transformed fold-change threshold: 1.5) for the selected cells against all other cells.

For comparison, we obtained a list of marker genes that was based on an atlas of fetal gene expression^32^. To facilitate a fair comparison to these marker genes, we selected matching numbers of CellWhisperer-identified organ marker genes (using a flexible log_2_ fold-change threshold). We then compared the number of papers in PubMed that co-mentioned each gene name and the corresponding organ in titles, keywords or abstracts using the following PubMed query (run with Biopython’s entrez.esearch function): ‘<organ> AND <gene>’.

Gene set enrichment was assessed using gseapy’s enrichr method^80^ for the gene set libraries GO_Biological_Process_2023, GO_Molecular_Function_2023, GO_Cellular_Component_2023 and KEGG_2021_Human. Spatial expression patterns for specific genes and lineage gene sets were derived from a corresponding Carnegie stage 8 embryo dataset^33^ that is accessible online (https://cs8.3dembryo.com).

### Multimodal training dataset of chat conversations

To enable natural-language chats with CellWhisperer, we fine-tuned the Mistral 7B LLM with conversations about transcriptomes and cell states. We generated 106,610 such conversations for transcriptomes from our training dataset of 1,082,413 GEO and CELLxGENE Census data points. To mitigate abundance biases in these repositories, we took a weighted subsample with sampling probabilities that were inversely proportional to the local point density calculated with densMAP^81^, such that transcriptome–text pairs in lowly covered regions were preferentially picked. For each of these subsampled transcriptome–text pairs, we generated a chat-like conversation using one of two LLMs (GPT-4 or Mixtral) on the basis of (1) the transcriptome’s top 50 most highly expressed genes (based on normalized expression across all transcriptomes in the dataset to improve the representation of lowly expressed but important genes such as certain transcription factors); (2) the top 50 GSVA-derived gene sets; and (3) the transcriptome’s textual annotation. We prepared conversations in four different ways, resulting in ‘simple’, ‘detailed’, ‘complex’ and ‘conversational’ chats. The LLM prompts and examples of the generated conversations are shown in Supplementary Note 2.

- Simple chats were generated for 10,000 transcriptomes by using a generic question (randomly selected from a list of ten manually prepared candidates: ‘What does the sample represent?’, ‘What do these cells represent?’, etc.) and the transcriptome’s generated textual annotation as the answer.
- Detailed chats were generated for 10,000 transcriptomes using an LLM (GPT-4 through the OpenAI API) with zero-shot prompting and the same questions as in the simple chats. GPT-4 produced much more extensive answers compared to the transcriptome’s textual annotations used in the simple chats.
- Complex chats were generated for 5,000 transcriptomes using an LLM (GPT-4 through the OpenAI API) with a few-shot prompt using pre-action reasoning^59^ to produce more profound question-answer pairs.
- Conversational chats for 81,610 transcriptomes were generated using an LLM (Mixtral 8x7b) with a few-shot prompt that instructed a natural conversation with multiple questions and answers between a researcher and an AI assistant about the biological state of the corresponding sample or cell cluster.

### Multimodal architecture and training of the CellWhisperer chat model

The CellWhisperer chat model follows the LLaVA approach^14^. We initialized a two-layer adapter module that transforms the 2,048-dimensional CellWhisperer multimodal embedding for a bulk or single-cell transcriptome into eight 4,096-dimensional embeddings, corresponding to eight token embeddings in the Mistral 7B LLM^13^, which is the basis for the CellWhisperer chat model. To train our model on the basis of the training conversations, we passed transcriptome-derived token embeddings alongside their corresponding questions to the LLM and optimized the LLM and the transcriptome–token adapter to generate the corresponding answers using the original autoregressive learning objective of the Mistral LLM, with a loss mask for the question and the transcriptome-related parts of the conversations. In a first training step, we kept the LLM frozen and trained the adapter layers with supervision on an extended version of the simple chats that included our entire training dataset (1,082,213 question-answer pairs) for one epoch. In a second training step, we unfroze the Mistral LLM and fine-tuned both the LLM and the adapter layers on the 106,610 generated training conversations for one epoch.

LLM fine-tuning was performed on four A100 80-GB GPUs with a total runtime of 3 hours. The generated conversation datasets and model checkpoints are available for download on the project website.

### Evaluation of the CellWhisperer chat model

To evaluate the CellWhisperer chat model, we created two question-answer conversation datasets and assessed CellWhisperer’s propensity for the correct answers, quantified on the basis of its predicted output token probabilities using the perplexity metric. This metric is defined as the exponentiated average negative log-likelihood of the sequence of tokens that correspond to the correct (ground truth) answer for a given prompt (that is, the response to the question). In other words, it measures the degree of surprise for the model when confronted with an answer to a given pair of a question and the corresponding transcriptome embedding.

The first dataset (termed Evaluation Conversations) provides a general evaluation of CellWhisperer’s chat functionality by randomly selecting 200 of the 106,610 training conversations, half based on GEO (bulk transcriptomes) and half based on CELLxGENE Census (pseudo-bulk transcriptomes from scRNA-seq data), with proportional representation of simple, complex and conversational chats. These conversations were trimmed to the first question-answer pairs, text that did not refer to biological insights was manually removed, and the data points associated with these conversations were excluded from CellWhisperer embedding and chat model training. To assess how well CellWhisperer took the transcriptome data into account when generating its chat response, we computed the perplexity for each test conversation not only with the matched transcriptome embedding but also with 30 mismatched transcriptome embeddings, randomly sampled from the dataset. Against this background, we reported the quantile of the matched-transcriptome perplexity.

The second dataset (termed Cell Type Conversations) assesses how well the CellWhisperer chat model learned information about the cell types associated with the transcriptome embeddings. For each of the 177 cell types in Tabula Sapiens, we sampled up to 100 pseudo-bulk transcriptomes, resulting in a total of 15,158 individual transcriptomes. Conversations in this dataset all contain the same question (‘Which cell type is this cell?’) and use the annotated cell type label as a natural-language answer by prefixing them with the following text: ‘This cell is a …’. To evaluate the preference of a given transcriptome for its annotated cell type, we calculated the perplexity against two backgrounds: (1) by randomly swapping the transcriptome embeddings (30 times, as above) and (2) by randomly swapping the cell type answer texts (176 times, to all other possible cell types).

For both datasets we compared the perplexity quantile scores of the CellWhisperer chat model with two text-only LLMs: Mistral 7B (without fine-tuning) and the much larger Llama 3.3 with 70 billion parameters, representing state-of-the-art LLMs (we could not compute the perplexity for closed models such as GPT-4 or Gemini as they do not provide output token probabilities). We provided the transcriptome context to these text-only LLMs through the following prepended prompt, containing the list of dataset-normalized top 50 genes: ‘USER: Respond to my request regarding a sample of cells characterized by its top expressed genes being <top 50 genes>. AI: Sure. What is your request?’. In the same manner, we assessed the impact of providing the list of top 50 genes, alongside the transcriptome embedding, to the CellWhisperer chat model.

### Integration of CellWhisperer into CELLxGENE Explorer, web hosting and user-provided dataset processing

We integrated CellWhisperer into CELLxGENE Explorer for web-based analysis of scRNA-seq data^15^ (version 1.2.0; https://docs.cellxgene.cziscience.com). To that end, we added a chat box to the CELLxGENE Explorer user interface and implemented two API endpoints: (1) natural-language chat functionality to retrieve information about a selected group of cells and (2) search interface to obtain CellWhisperer scores for user-provided free-text queries, which are displayed as cell-level color maps. User requests that start with ‘show’, ‘show me’, ‘search’ or ‘search for’ are always routed to the second endpoint. Other requests are routed to the second endpoint only if the user has not selected any cells. The CellWhisperer chats inside CELLxGENE Explorer are primed with a user-hidden prompt (Supplementary Note 2) designed to reduce the prevalence of donor-specific metadata in the responses. This prompt also includes the top 50 expressed genes for the selected cells, as we observed better biological interpretability of responses and a slight performance improvement when providing the CellWhisperer chat model with these gene names in addition to the transcriptome embedding (Extended Data Fig. 4c).

We host the CellWhisperer-augmented version of CELLxGENE Explorer as a web tool using docker/podman/docker-compose. Each dataset receives its dedicated server job and these jobs are jointly exposed through an nginx web server. CellWhisperer’s embedding and chat capabilities are hosted through independent API server jobs, which are accessible online to enable running the software tool locally on computers without a GPU.

User-provided datasets have to be prepared for CellWhisperer analysis using an automated pipeline (instructions are provided in the source code repository; https://github.com/epigen/cellwhisperer). Their interactive analysis requires a local installation of the CellWhisperer web tool, as it is currently not supported to upload user-provided datasets to our publicly accessible CellWhisperer instance (https://cellwhisperer.bocklab.org). The pipeline first processes all transcriptome measurements using the CellWhisperer embedding model, facilitating efficient CellWhisperer scoring. Next, a UMAP is calculated for the embeddings, followed by clustering with the Leiden algorithm and generation of a brief textual annotation for each cluster. Then, the CellWhisperer chat model processes the cluster-averaged transcriptome embeddings to generate detailed textual descriptions, which are subsequently condensed into brief textual annotations with an LLM such as GPT-4 (through the OpenAI API, used in this study) or Mixtral 8x7B (which can be run locally and is also supported by our source code). All prompts are provided in Supplementary Note 2. The user-provided dataset with all preprocessed elements is stored as a single h5ad object for use with the CellWhisperer web tool. This data-processing pipeline is run with a single shell command and was used to process the datasets for Figs. 1, 3–5 and for the demonstration video (Supplementary Video 1). Data processing has a runtime on the order of minutes for typical datasets on a compute node with one A100 GPU or on the order of hours when relying on a standard laptop without a GPU.

### Conventional bioinformatics analysis of the Colonic Epithelium dataset

To compare the CellWhisperer analysis of the Colonic Epithelium dataset to a conventional bioinformatics analysis of the same data, we wrote custom Python code following established practices for scRNA-seq analysis. We retrieved the total-read-normalized and log1p-transformed scRNA-seq read count matrix from GEO (GSE116222) and used the inverse transform (exp1m) to obtain gene expression profiles that formed the basis for our analysis. We used scVI^44^ with sampleID as a batch variable to remove batch effects (parameters: 4,000 highly variable genes, n_layers = 2, n_latent = 30, gene_likelihood = ‘nb’). Next, we computed and plotted a UMAP based on scVI’s latent space using scanpy^82^. We used CellTypist^45^ with the ‘Cells_Intestinal_Tract.pkl’ model^83^ to automatically annotate cell types, first with majority voting (as recommended by the CellTypist instructions) and then without (which allowed us to identify transcriptomes that were annotated as stem cells). On the basis of these annotations, we determined differentially expressed genes for selected cell types in a one-versus-all manner using scanpy and the Wilcoxon rank sum test. Lastly, to obtain a measure of stemness of inflamed and noninflamed cells, we computed a gene module score based on a corresponding gene set^46^.

### Reporting summary

Further information on research design is available in the Nature Portfolio Reporting Summary linked to this article.

## Online content

Any methods, additional references, Nature Portfolio reporting summaries, source data, extended data, supplementary information, acknowledgements, peer review information; details of author contributions and competing interests; and statements of data and code availability are available at https://doi.org/10.1038/s41587-025-02857-9.

## Supplementary information

Supplementary Information Supplementary Notes 1–3.

Reporting Summary

Supplementary Table 1 Performance evaluation of the CellWhisperer embedding model based on the prediction of cell characteristics.

Supplementary Table 2 Performance evaluation of the CellWhisperer embedding model based on gene sets.

Supplementary Table 3 CellWhisperer-identified marker genes of human organ development.

Supplementary Video 1 Interactive single-cell RNA-seq data analysis with the CellWhisperer web tool.

## Data Availability

CellWhisperer was trained on publicly available datasets obtained from GEO and CELLxGENE Census. All training data are available from their original sources. Full details are provided in the Methods and source code (https://github.com/epigen/cellwhisperer), which implements automatic data download. The model weights and LLM-curated datasets for training and model evaluation are available from the project website (https://cellwhisperer.bocklab.org).

## Code availability

The source code underlying this project is available on GitHub (https://github.com/epigen/cellwhisperer), which includes the CellWhisperer model and dataset processing code and our adapted version of CELLxGENE Explorer with integrated CellWhisperer functionality. These components enable the analysis of user-supplied datasets for interactive analysis on Linux-based compute infrastructure by running a local version of the CellWhisperer web tool. We further provide automatic start-to-finish pipelines to reproduce all analyses described in this paper. Where we build on external codebases (Geneformer, LLaVA and CELLxGENE Explorer), we forked, adapted and integrated them as submodules in the CellWhisperer GitHub repository. In addition to the CellWhisperer source code, we provide the trained CellWhisperer model as a PyTorch Lightning checkpoint for download on the project website (https://cellwhisperer.bocklab.org). Detailed instructions on installing CellWhisperer and reproducing this study are provided in the GitHub repository.
