# Supplementary material for: Multimodal learning enables chat-based exploration of single-cell data
Source: Nat Biotechnol. 2025 Nov 11;44(10):1722–32. doi: 10.1038/s41587-025-02857-9 (PMC13654808; doi:10.1038/s41587-025-02857-9)
Supplement: Supplementary file 1 [file 41587_2025_2857_MOESM1_ESM.pdf]

nature biotechnology

Supplementary information https://doi.org/10.1038/s41587-025-02857-9

Multimodal learning enables chat-based exploration of single-cell data

In the format provided by the authors and unedited

Supplementary Note 1: CellWhisperer usage examples for single-cell RNA-seq data exploration with natural language

CellWhisperer establishes natural language as a channel for interactive, exploratory analysis of scRNA-seq data. This Supplementary Note illustrates CellWhisperer's natural-language features with concrete usage examples. All examples are based on the CellWhisperer web tool integrated into CELLxGENE Explorer.

Table of contents

Functionality of the CellWhisperer web tool 2

1) Search/annotate cells with natural-language queries 2
2) Select cells and chat about them 2
3) Inspect the CellWhisperer-generated labels of cell clusters 3
4) Visualize gene expression (CELLxGENE core functionality) 3

Usage examples 4

Dataset: Tabula Sapiens (Fig. 4; https://cellwhisperer.bocklab.org/tabulasapiens) 4

Dataset: Colonic Epithelium (Fig. 5; https://cellwhisperer.bocklab.org/colonic_epithelium) 7

Dataset: GEO (Fig. 1; https://cellwhisperer.bocklab.org/geo) 12

Dataset: Human Development (Fig. 3; https://cellwhisperer.bocklab.org/development) 14

Functionality of the CellWhisperer web tool

This section presents key natural-language features of the CellWhisperer web tool with brief user instructions.

1) Search/annotate cells with natural-language queries

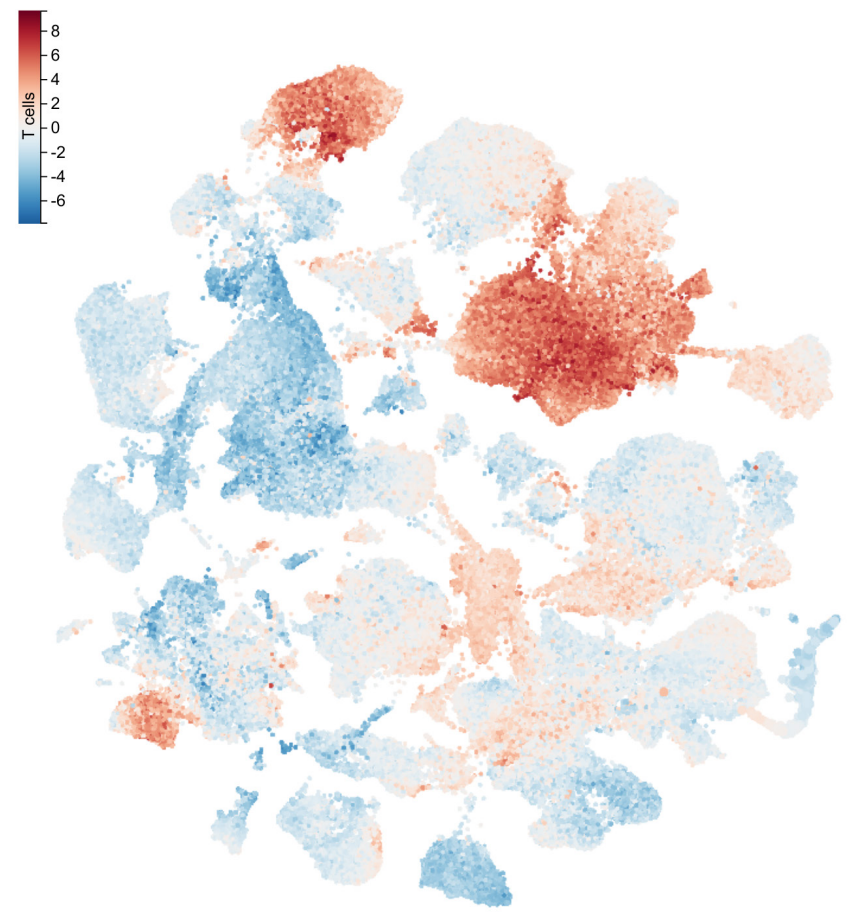

Type your query to highlight matched cells. This feature is automatically used if no cells are selected. Otherwise, it can be enforced by typing “Show me <query>”.

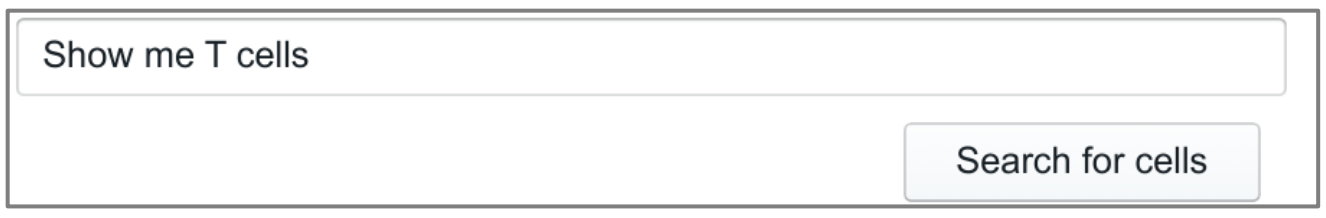

2) Select cells and chat about them

1. Select cells in one of three alternative ways
   a. *Visual selection*. Manually encircle the cell population of interest

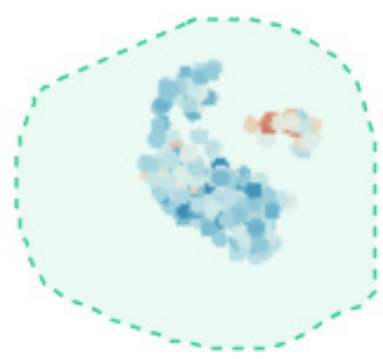

   b. *Score-based selection*. Draw a thresholding box in the histogram of CellWhisperer scores

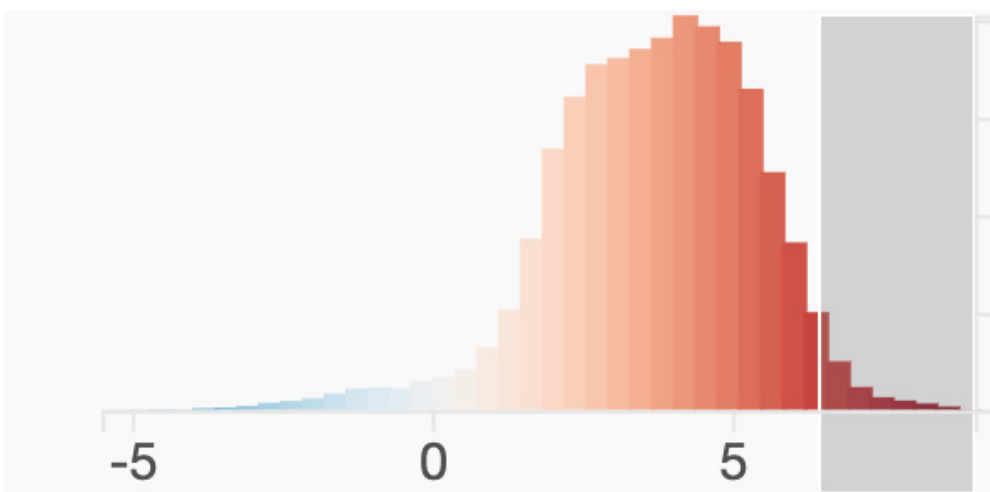

   c. *Annotation-based selection*. Select or deselect sample annotations

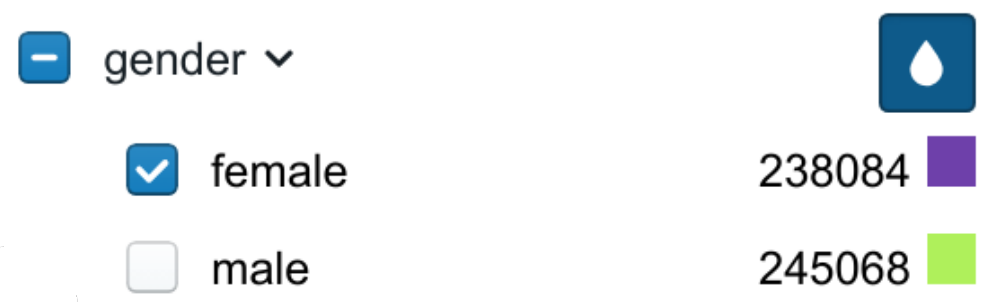

2. Initiate a conversation about the selected cells

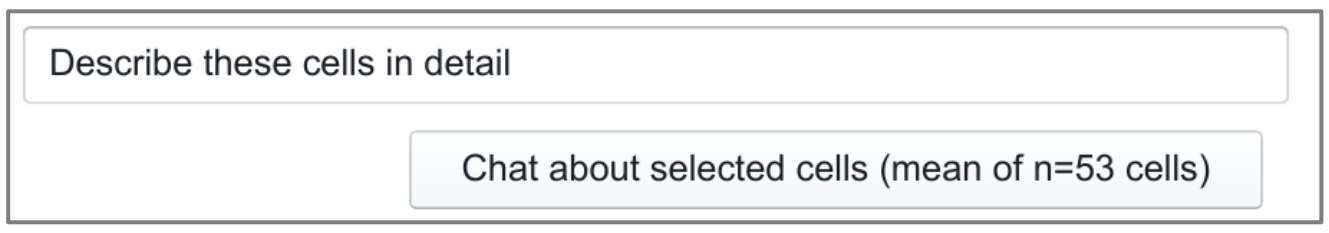

3. Follow up on the CellWhisperer responses by asking about specific genes or biological processes

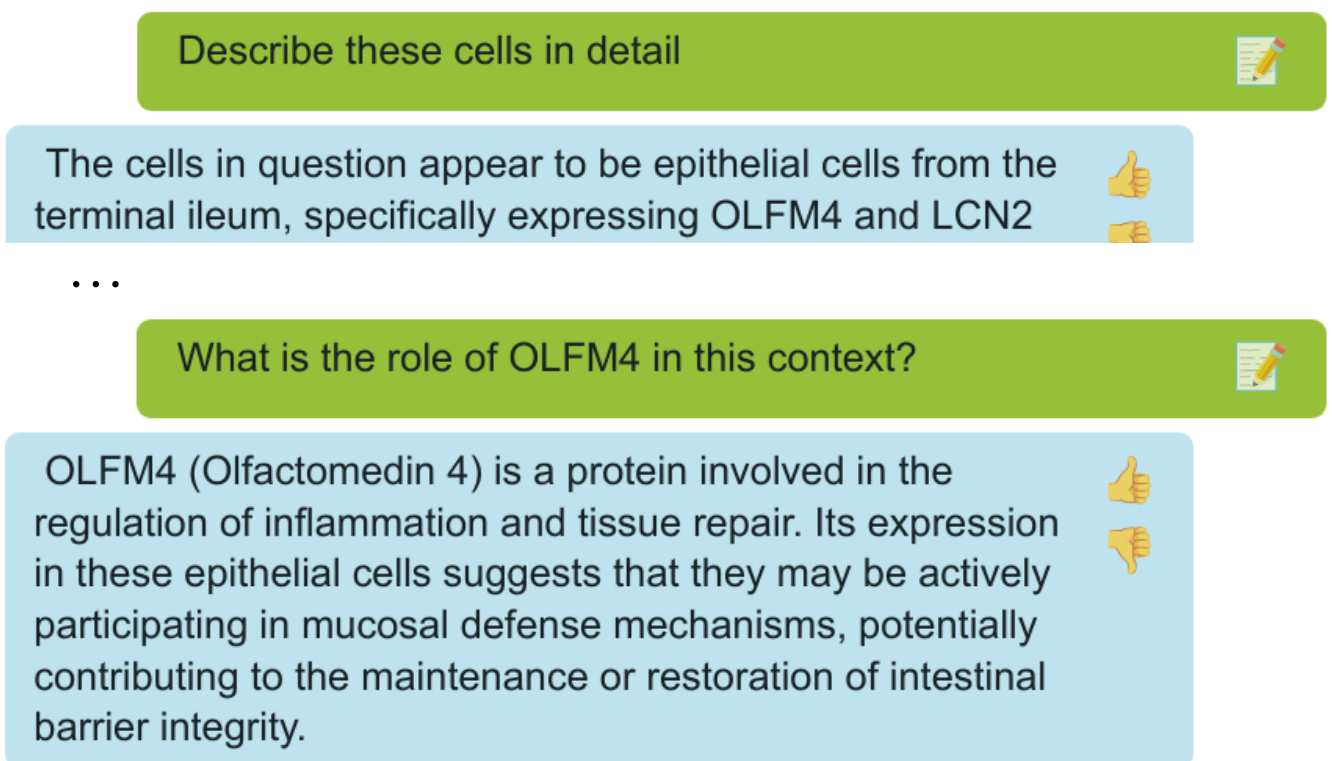

3) Inspect the CellWhisperer-generated labels of cell clusters

CellWhisperer automatically creates cluster labels in its preprocessing pipeline. They are accessible through the *cluster_label* field and can be projected on the UMAP:

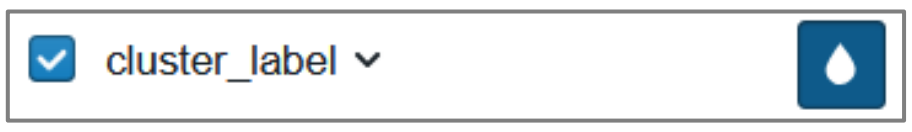

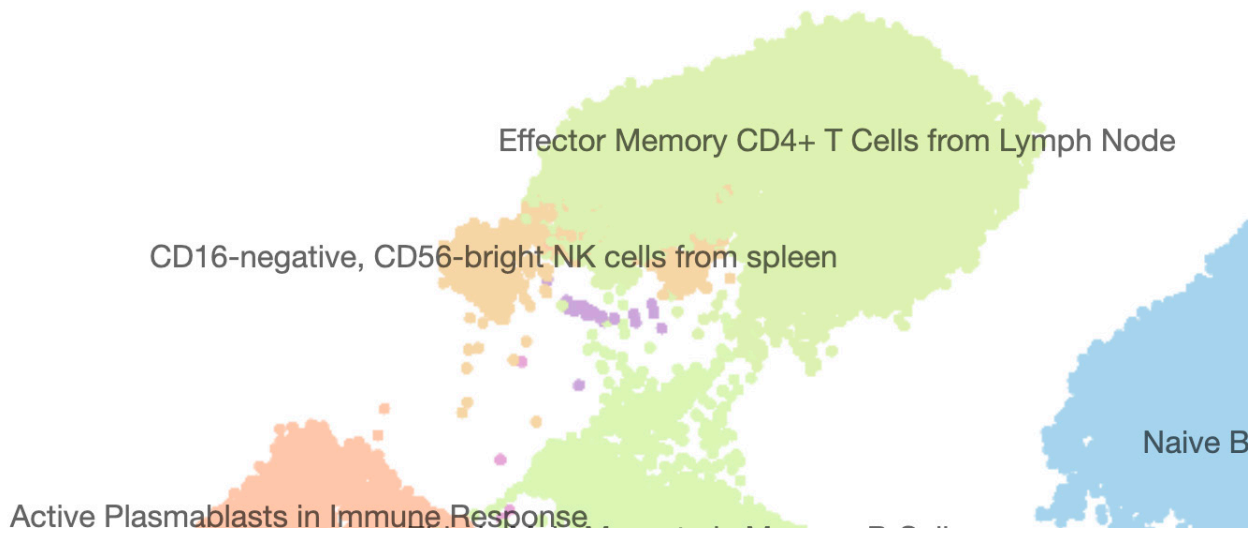

4) **Visualize gene expression** (CELLxGENE core functionality)

The CELLxGENE Explorer comes with various visual tools for interacting with transcriptomics data. For example, the expression of individual genes can be readily visualized through a input field.

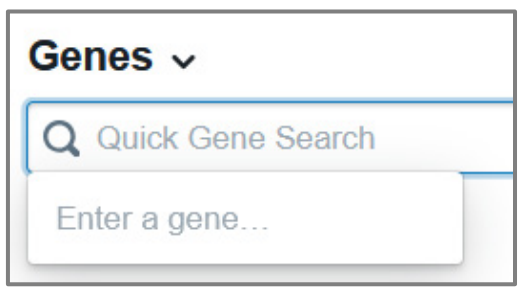

The following website describes CELLxGENE Explorer features that can be used with CellWhisperer: https://cellxgene.cziscience.com/docs/04__Analyze%20Public%20Data/4_1__Hosted%20Tutorials

Usage examples

Dataset: Tabula Sapiens (Fig. 4; https://cellwhisperer.bocklab.org/tabulasapiens)

Example 1: Structural cells with immune functions

- Annotate *"structural cells with immune functions"*

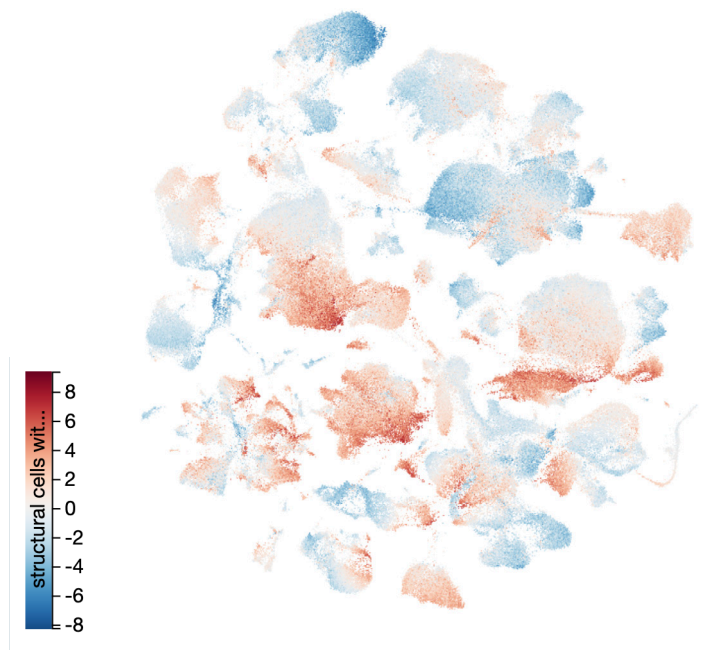

- Select one of the high-scoring clusters

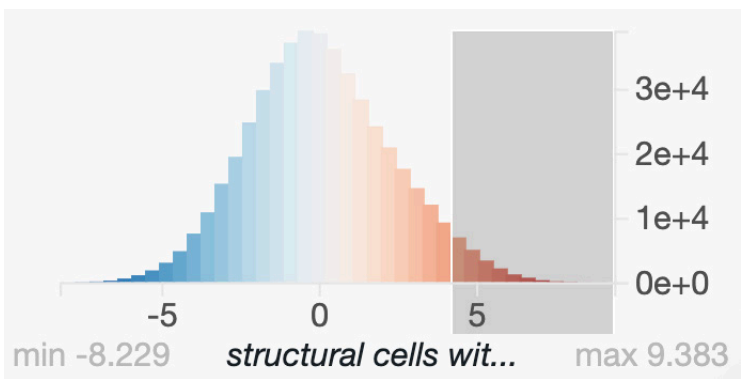

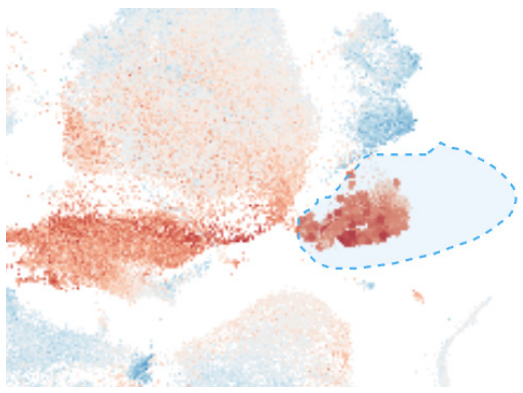

- Prompt CellWhisperer to describe the selected cells

Describe these cells in detail.

The cells in question appear to be fibroblasts derived from the decidua tissue of a female individual at 10 post-conception weeks. Their gene expression profile is characterized by high levels of extracellular matrix components (e.g., COL1A1, COL3A1), stress response genes (e.g., HSPA1A, HSPB1), and signaling molecules (e.g., MDK, FOS). This suggests that these fibroblasts are actively involved in tissue remodeling processes typical for early pregnancy, such as angiogenesis and immune modulation. Additionally, the presence of pathways related to various immune cells hints at a complex interaction between the fibroblasts and the immune system within the decidual environment. Overall, it seems these fibroblasts play crucial roles in supporting fetal development through structural support, immune regulation, and possibly contributing to the formation of the maternal-fetal interface.

- Ask a follow-up question about the biological functions mentioned in the CellWhisperer response

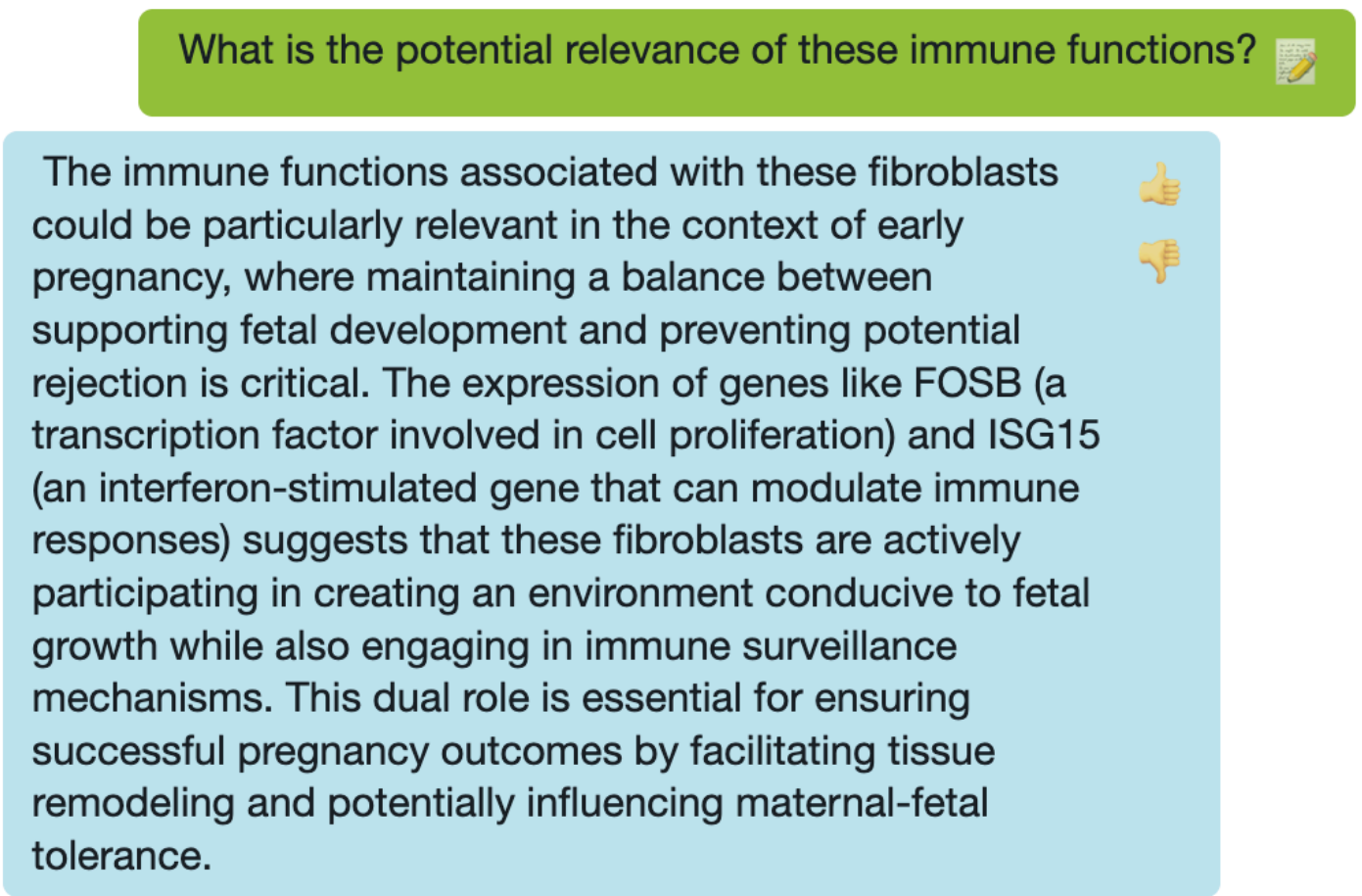

- Visualize the aggregate gene expression of two genes mentioned in the CellWhisperer response

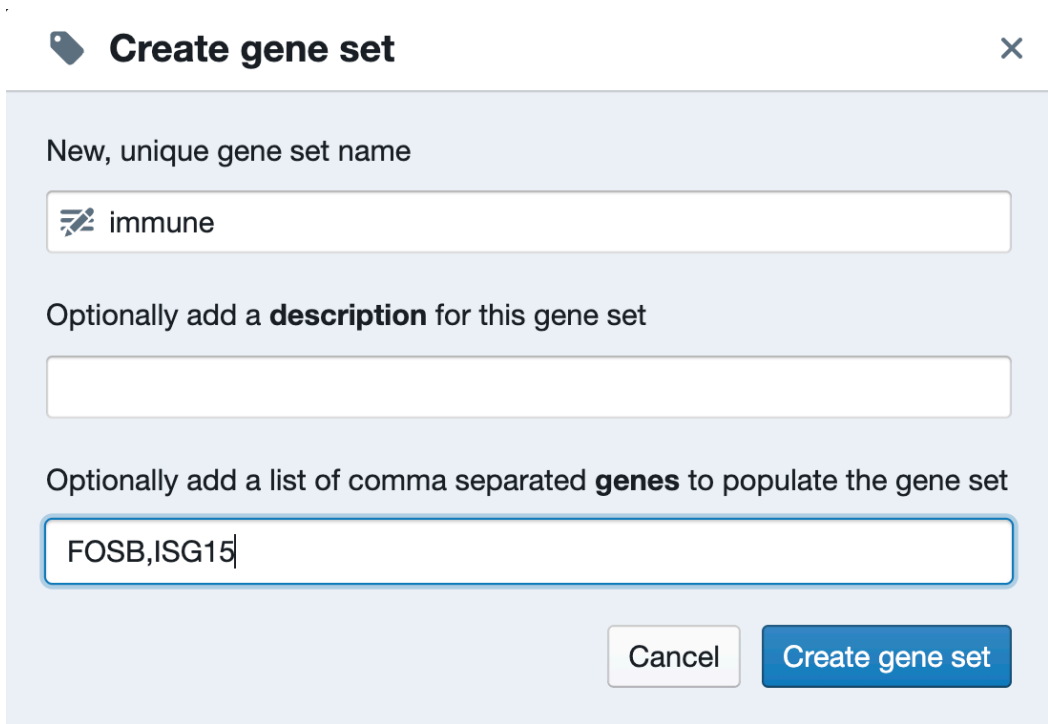

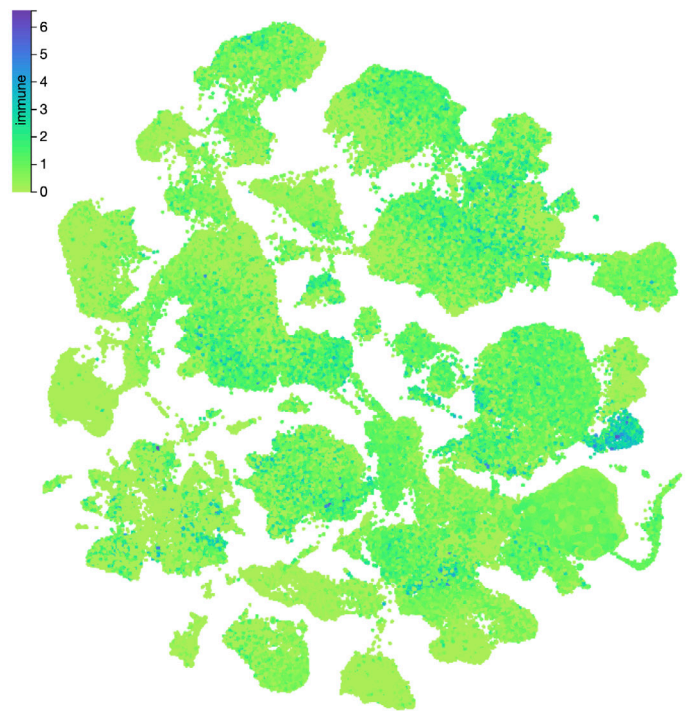

Example 2: Clarification about the cells annotated as *"kidney cortex"*

- Select cells based on CellWhisperer cluster label of interest

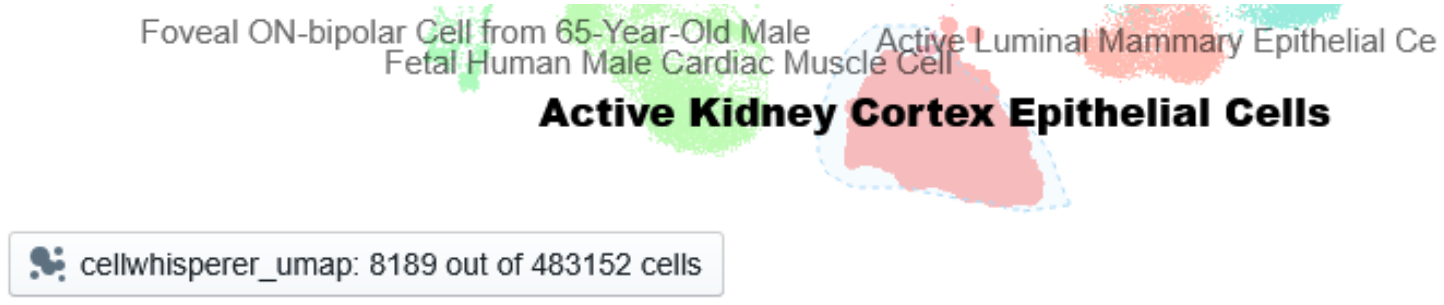

- Request a detailed description to confirm their annotation as *"kidney cortex"*

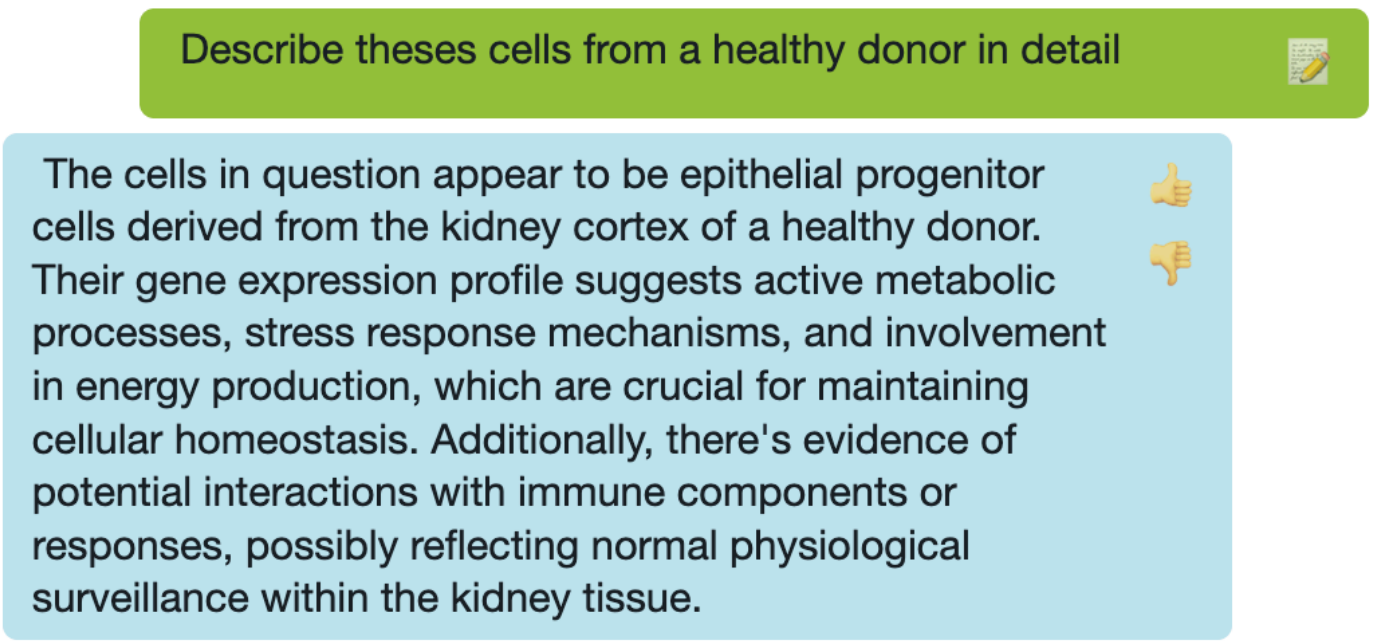

- Ask a follow-up question to clarify the biological term *"kidney cortex"*

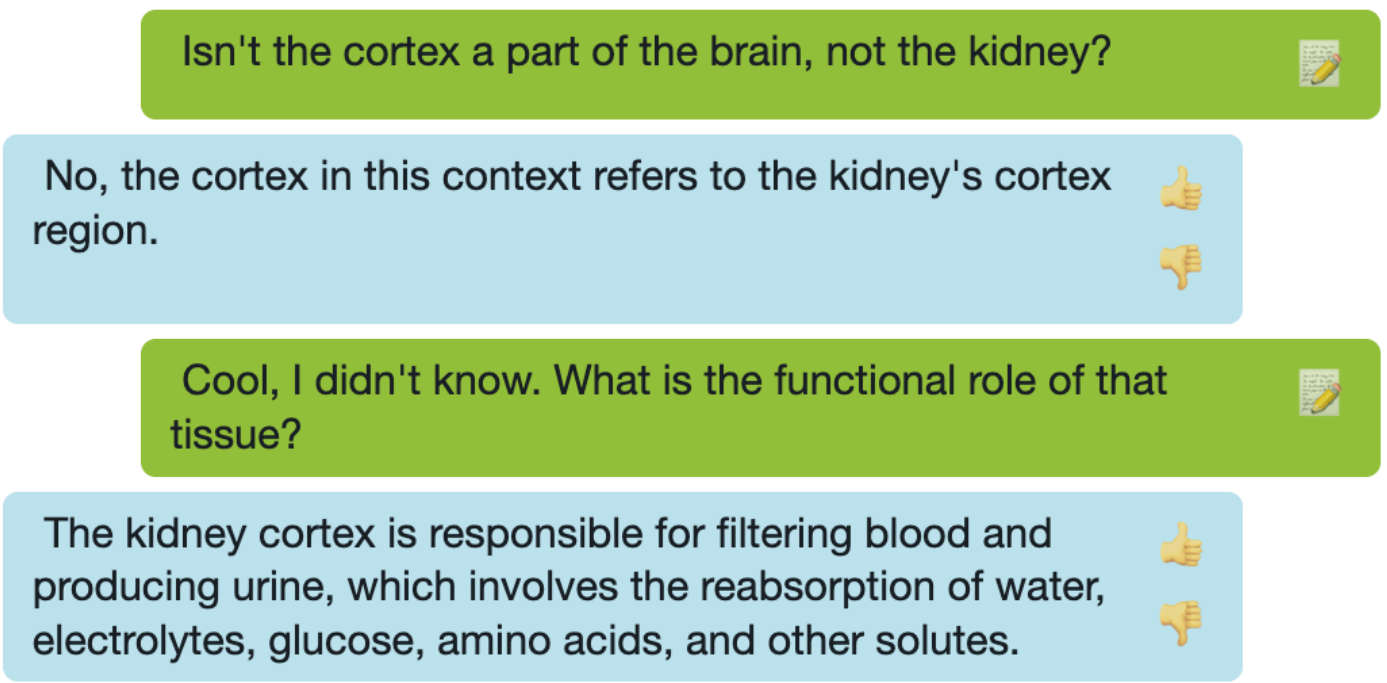

Dataset: Colonic Epithelium (Fig. 5; https://cellwhisperer.bocklab.org/colonic_epithelium)

Example 3: Investigate stem cell niches in colonic epithelial cells

- Investigate CellWhisperer cluster labels

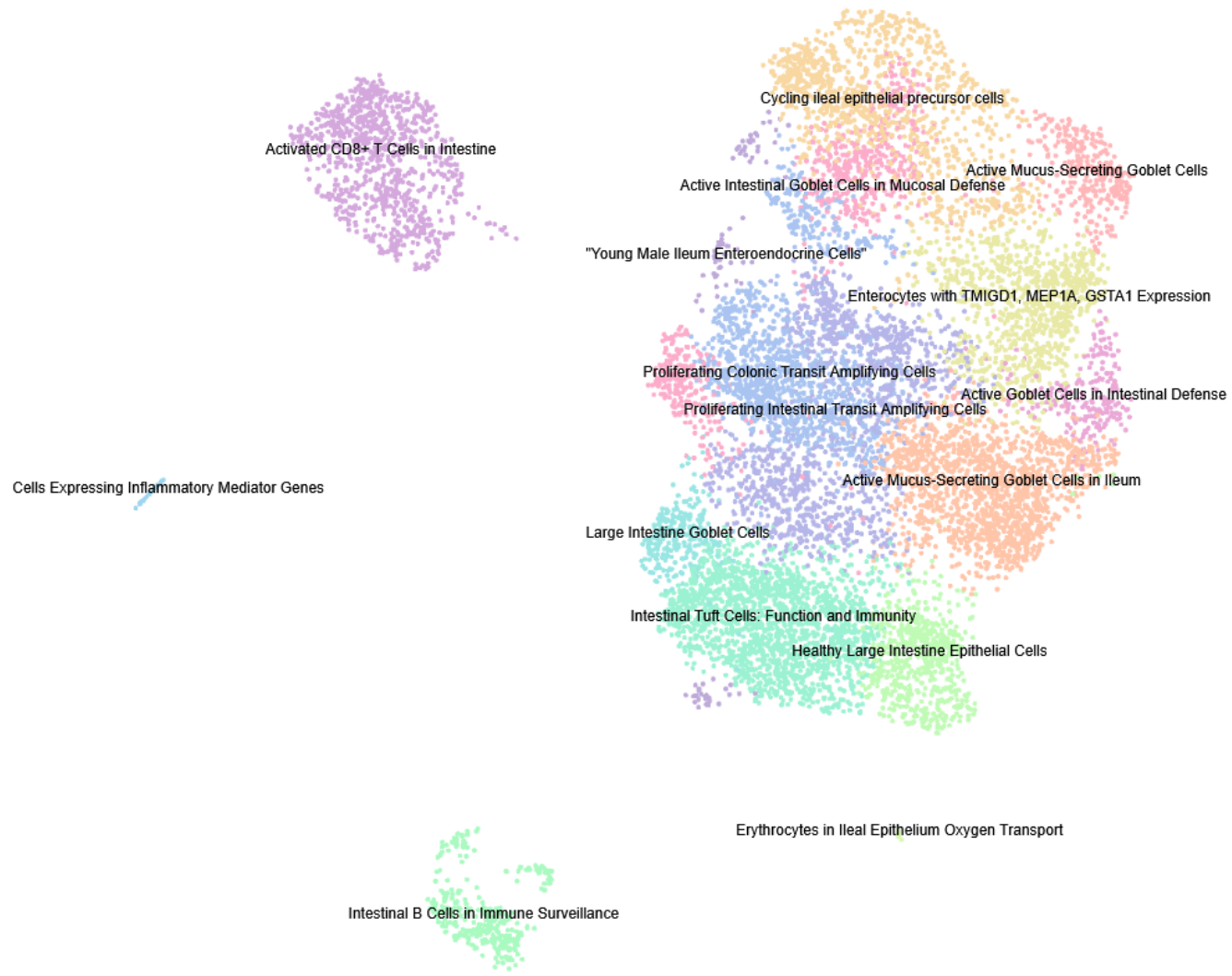

- Search cells using the query term *"stem cells"*

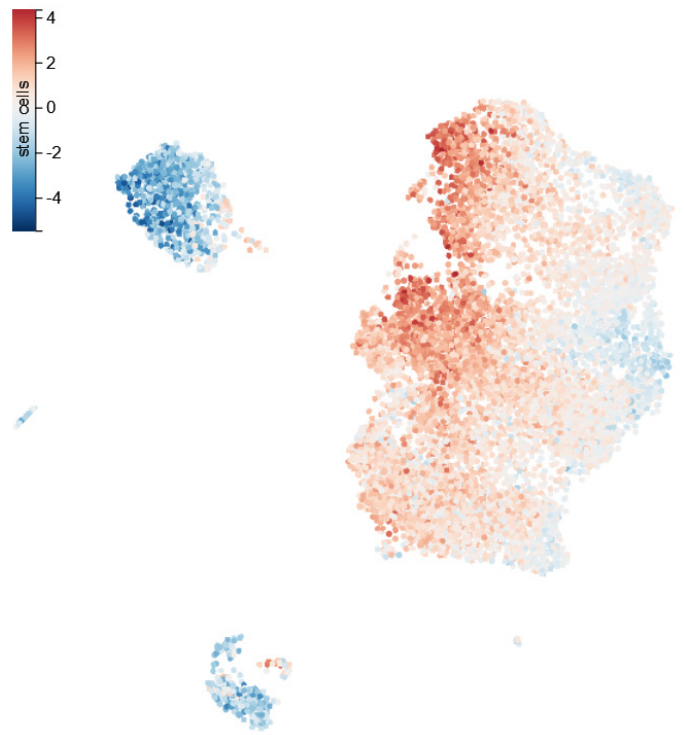

- Select top-scoring cells for query term *"stem cells"* in cluster *"Cycling ileal epithelial precursor cells"*

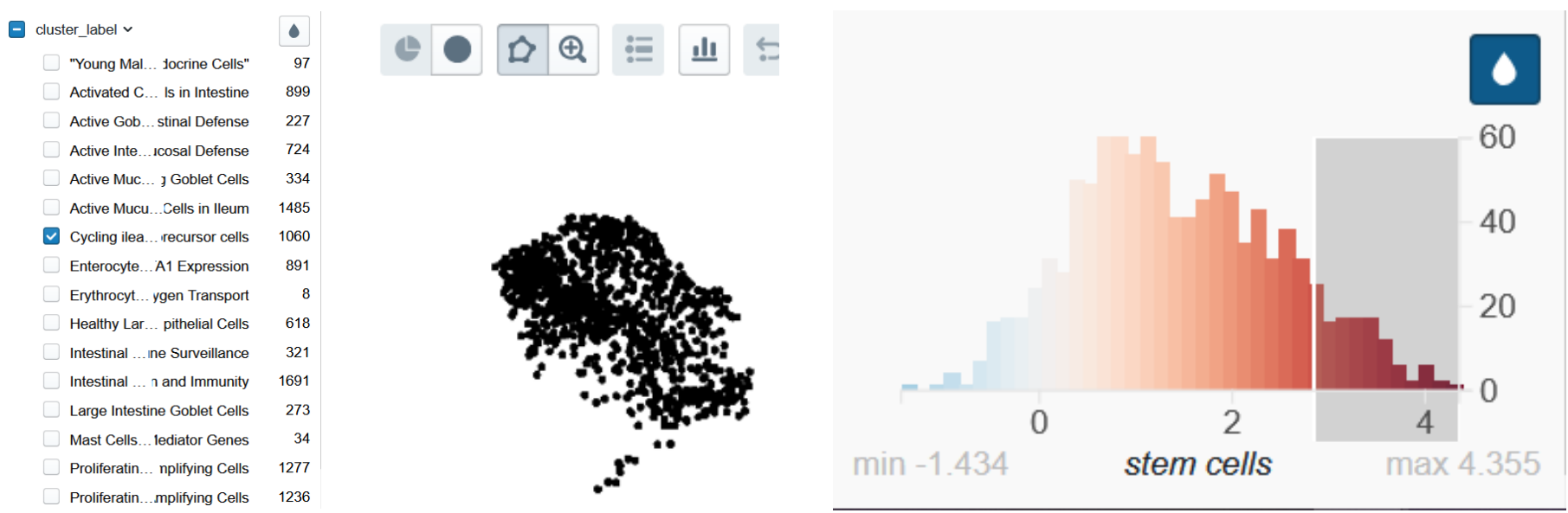

- Prompt CellWhisperer to describe the selected cells

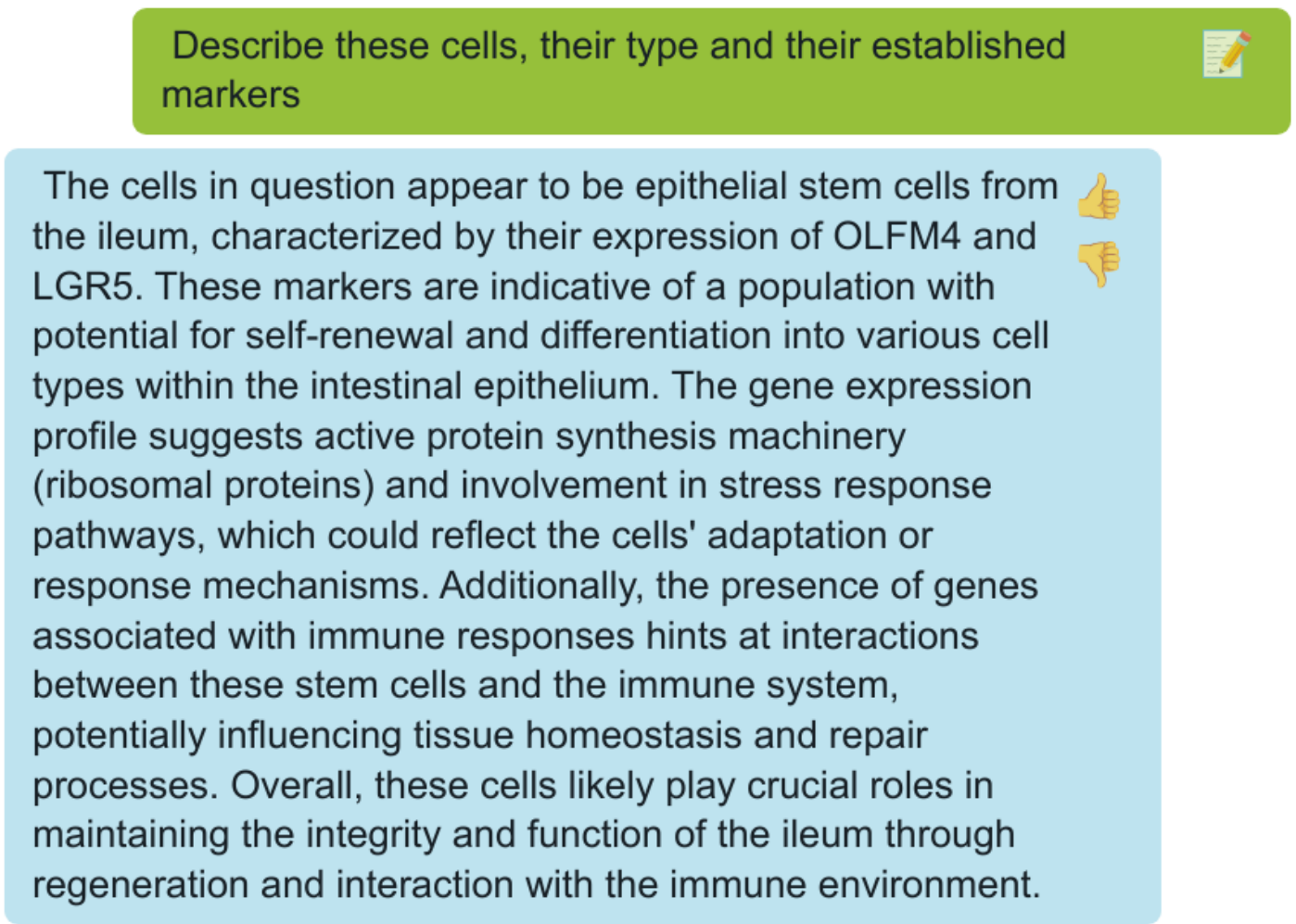

- Visualize gene expression of the mentioned gene (LGR5)

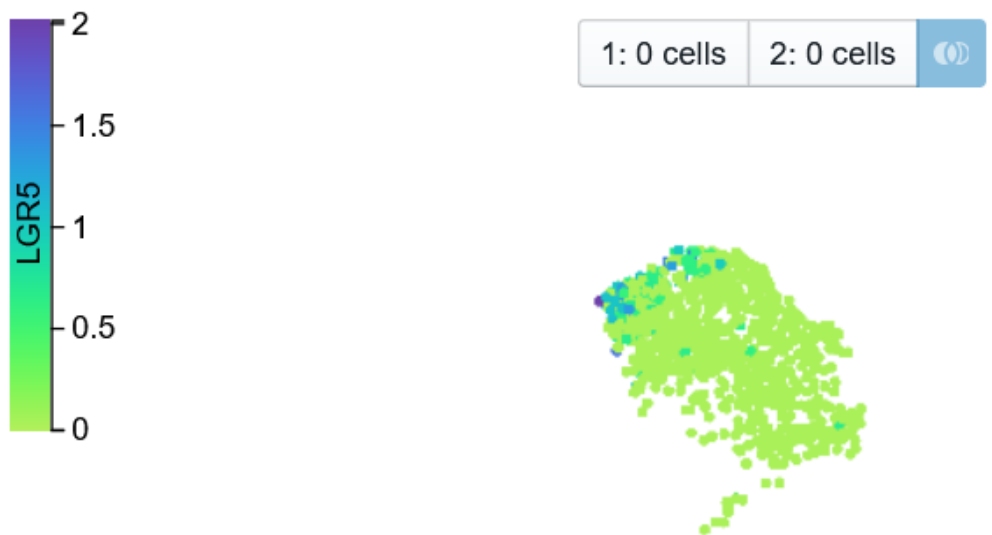

- Compare CellWhisperer scores for the *"stem cells"* query for inflamed and non-inflamed samples

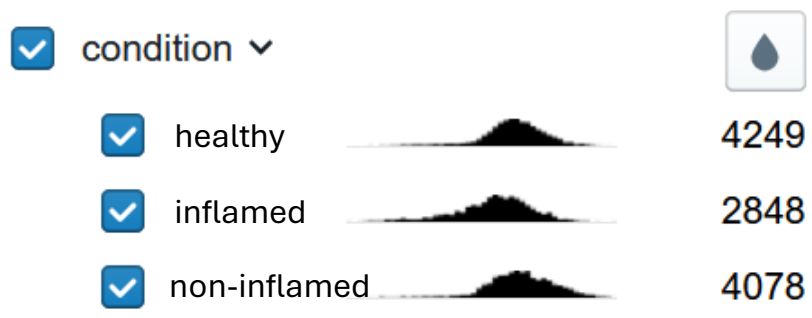

Example 4: Isolate cells in cell cycle S phase

- Search cells using the query term *"Cell cycle S phase cells"*

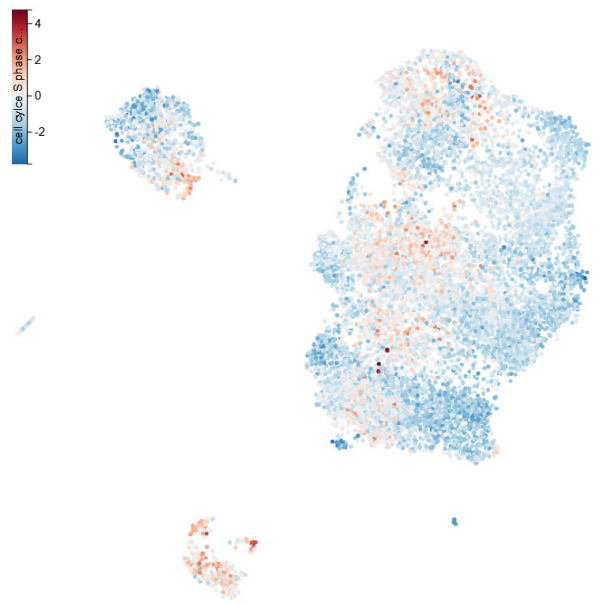

- Visualize gene expression of a known cell cycle S phase marker gene (CCNA2)

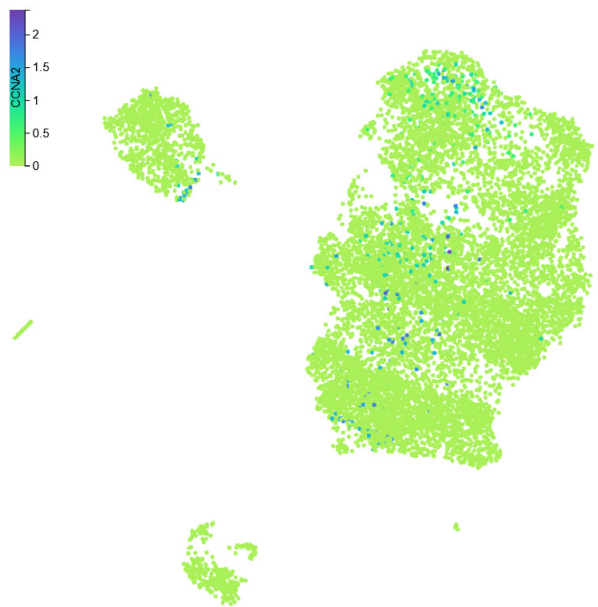

Example 5: Epithelial cells expressing immune receptors

- Search cells using the query term *"epithelial cells expressing immune receptors"*

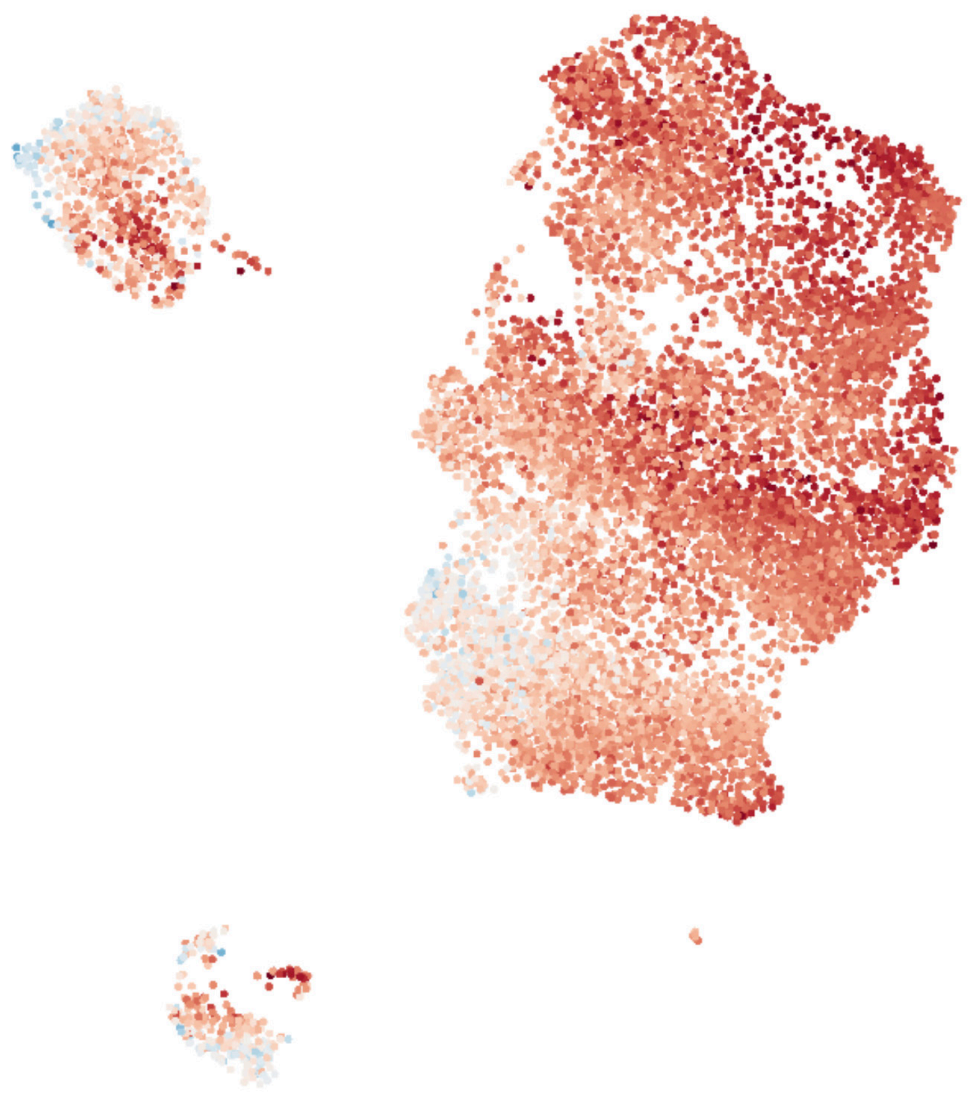

- Select and describe the top 1000 cells for this query term

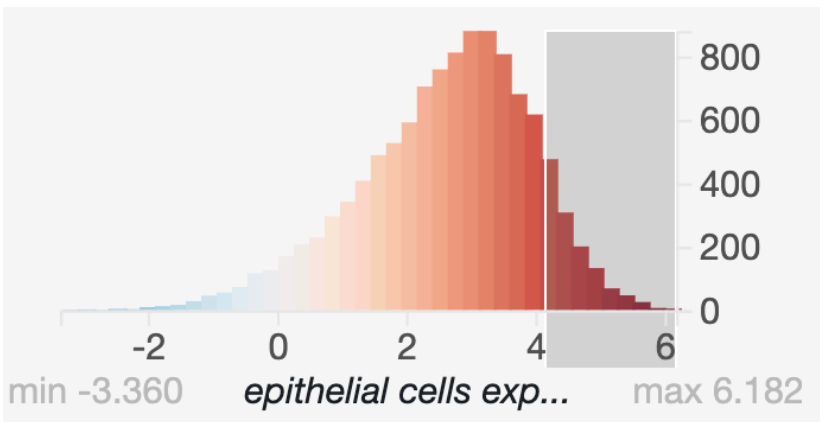

> Describe these cells in detail.

> The cells in question appear to be goblet cells from the ileum, characterized by a strong expression of genes involved in mucus production and secretion (e.g., TFF3), as well as markers indicative of their epithelial origin (e.g., KRT19). This suggests that these cells play a crucial role in maintaining intestinal barrier integrity and function within the small intestine. Additionally, the presence of immune-related gene sets hints at potential interactions between these goblet cells and the immune system, which could be relevant for understanding inflammatory responses or tissue repair processes in the context of Crohn's disease. Overall, it seems these cells are actively engaged in producing mucins and other molecules essential for protecting the intestinal lining while possibly interacting with various components of the immune system.

- Follow up on the mentioning of Crohn's disease

> wait, Crohn disease?

> Yes, the sample was taken from a patient with Crohn's disease. However, I cannot provide specific details on the donor or patient beyond what is already mentioned in the provided information.

- Cross-check plausibility against the sample annotations: the *"inflamed"* label corresponds to samples of patients of inflammatory bowel disease, of which Crohn's disease is a subtype

  Ratio of cells in the top 1000 cells for this query that were from inflamed samples as opposed to control samples from healthy individuals: 4.63

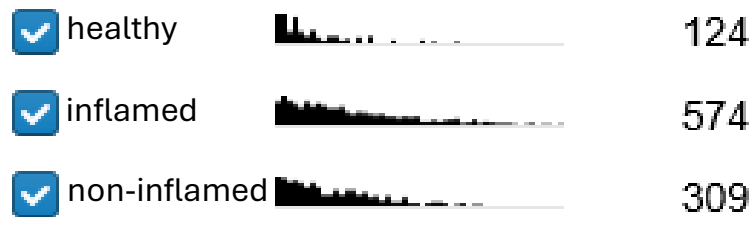

  Ratio of all cells in the dataset that were derived from inflamed samples as opposed to control samples from healthy individuals: 0.67

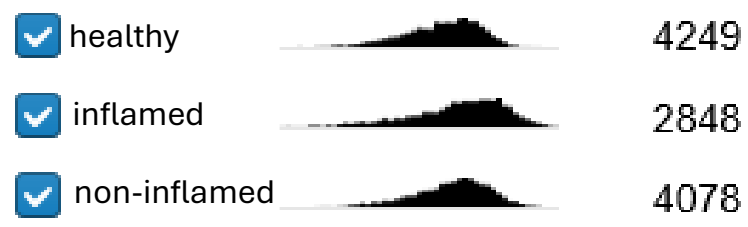

Dataset: GEO (Fig. 1; https://cellwhisperer.bocklab.org/geo)

Example 6: Explore extracellular matrix organization

- Select and describe the cell cluster labeled *"Cells actively remodeling extracellular matrix"*

> Describe these cells in detail.

> The cells in question appear to be cardiac myocytes that have been subjected to a reprogramming process using polycistronic human MEF2C, GATA4, TBX5, and miR-133. This process seems to have induced a complex state where the cells exhibit characteristics of both their original identity as cardiac myocytes and features reminiscent of various other cell types, including hematopoietic stem cells, myeloid progenitors, dendritic cells, and even non-cardiac tissues like skin melanocytes and mammary luminal epithelial cells. The gene expression profile suggests active extracellular matrix organization, collagen synthesis, and potential involvement in fibrosis or tissue repair processes. It's likely that these cells are at an intermediate stage of differentiation or reprogramming, reflecting the inherent complexity and challenges associated with cellular engineering.

- Follow up on collagen-related gene expression

> which collagen-related genes are expressed?

> The top expressed collagen-related genes include COL1A2, COL3A1, LUM, DCN, MGP, FAP, and ENSG00000289754. These genes are involved in various processes such as extracellular matrix organization, tissue remodeling, and fibrosis.

- Follow up on specific relevance to heart tissue

> are any of those relevant specifically in the heart tissue?

> Yes, COL1A2 and COL3A1 are known to be involved in heart tissue remodeling processes.

- Visualize expression of a mentioned gene (COL1A2)

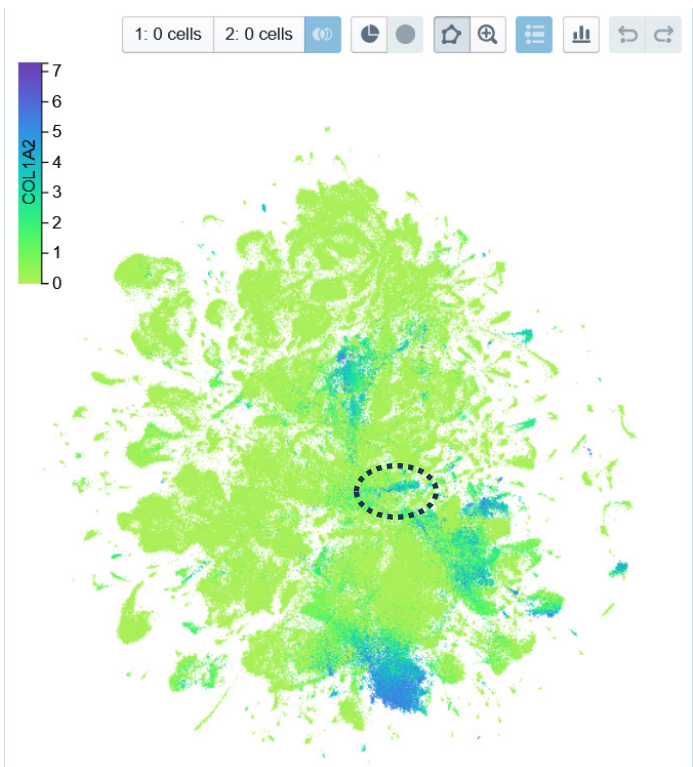

Example 7: Retinal precursor cells

- Select and describe cell cluster labeled *"Fetal retinal cells undergoing photoreceptor differentiation"*

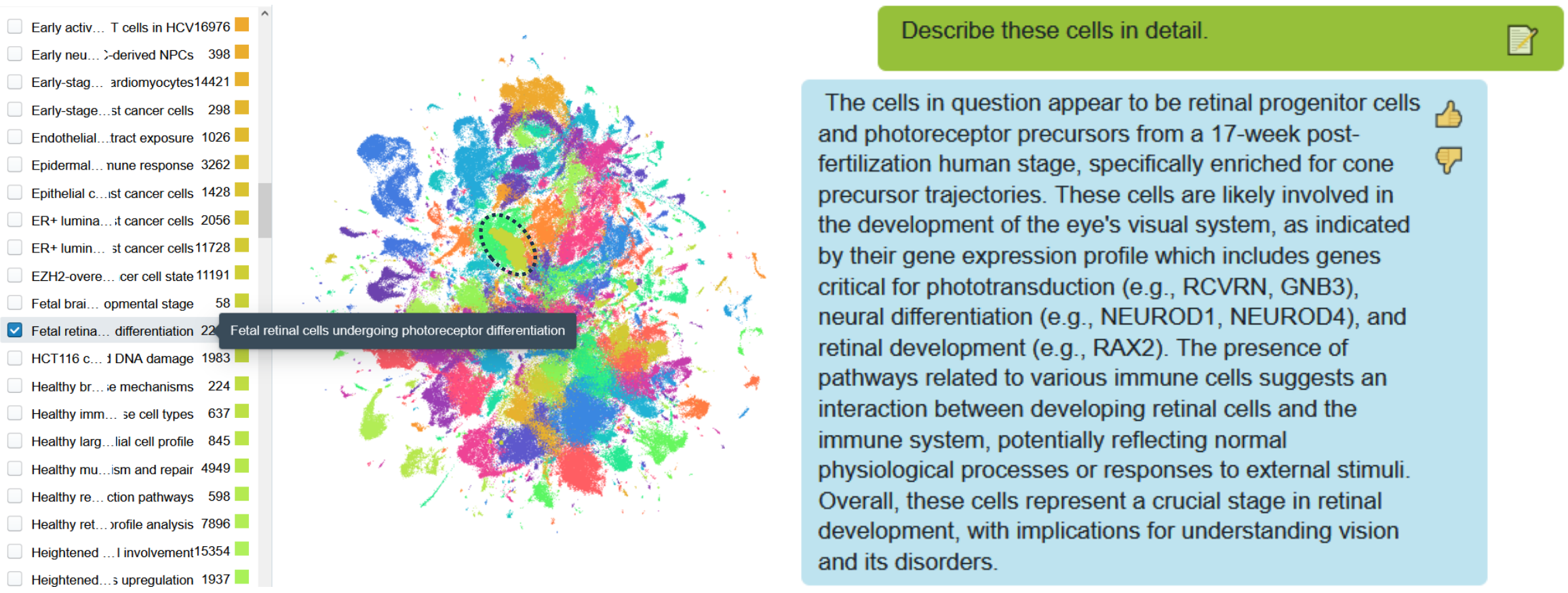

- Visualize the expression of a mentioned gene (RCVRN)

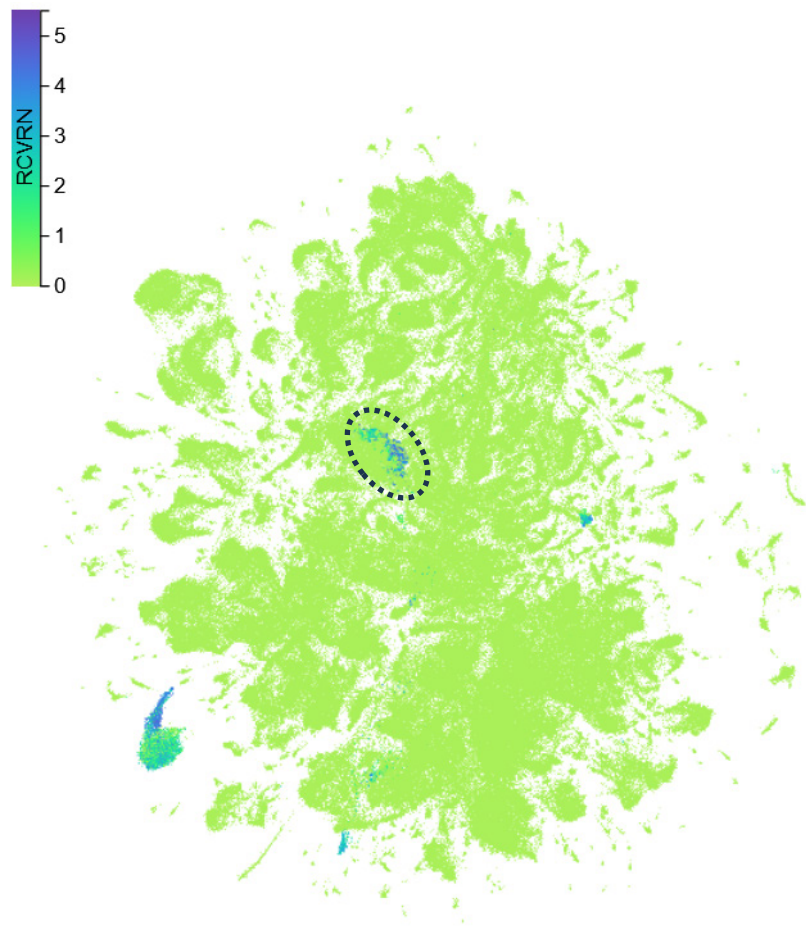

- Search for related cell clusters with the query term *"retinal cells"*

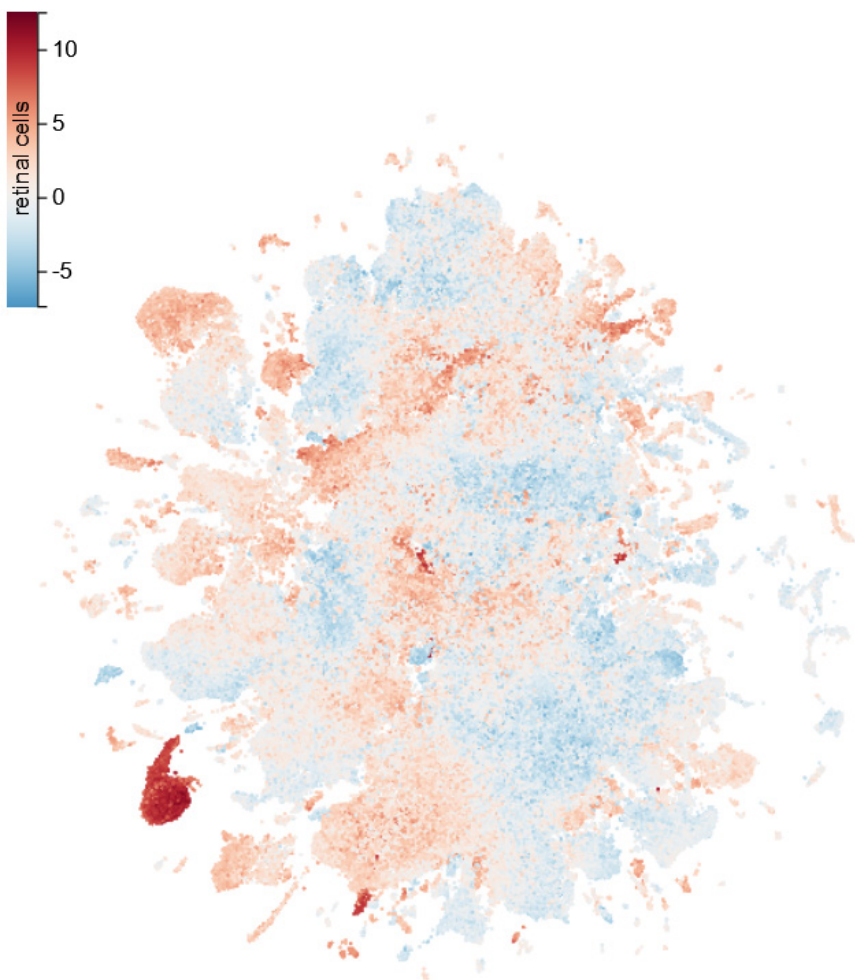

Dataset: Human Development (Fig. 3; https://cellwhisperer.bocklab.org/development)

Example 8: Mapping organogenesis in human embryonic development

- Search for cells with query term *“cerebrum”*

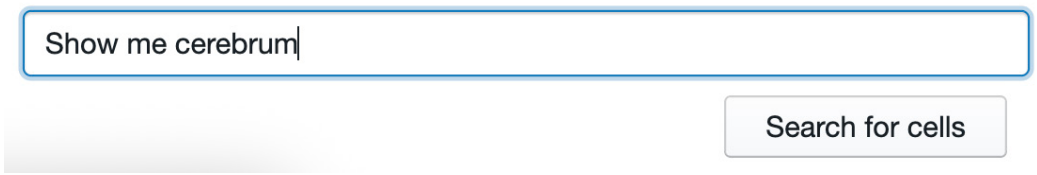

- Examine the distribution of CellWhisperer scores for different time points of embryonic development

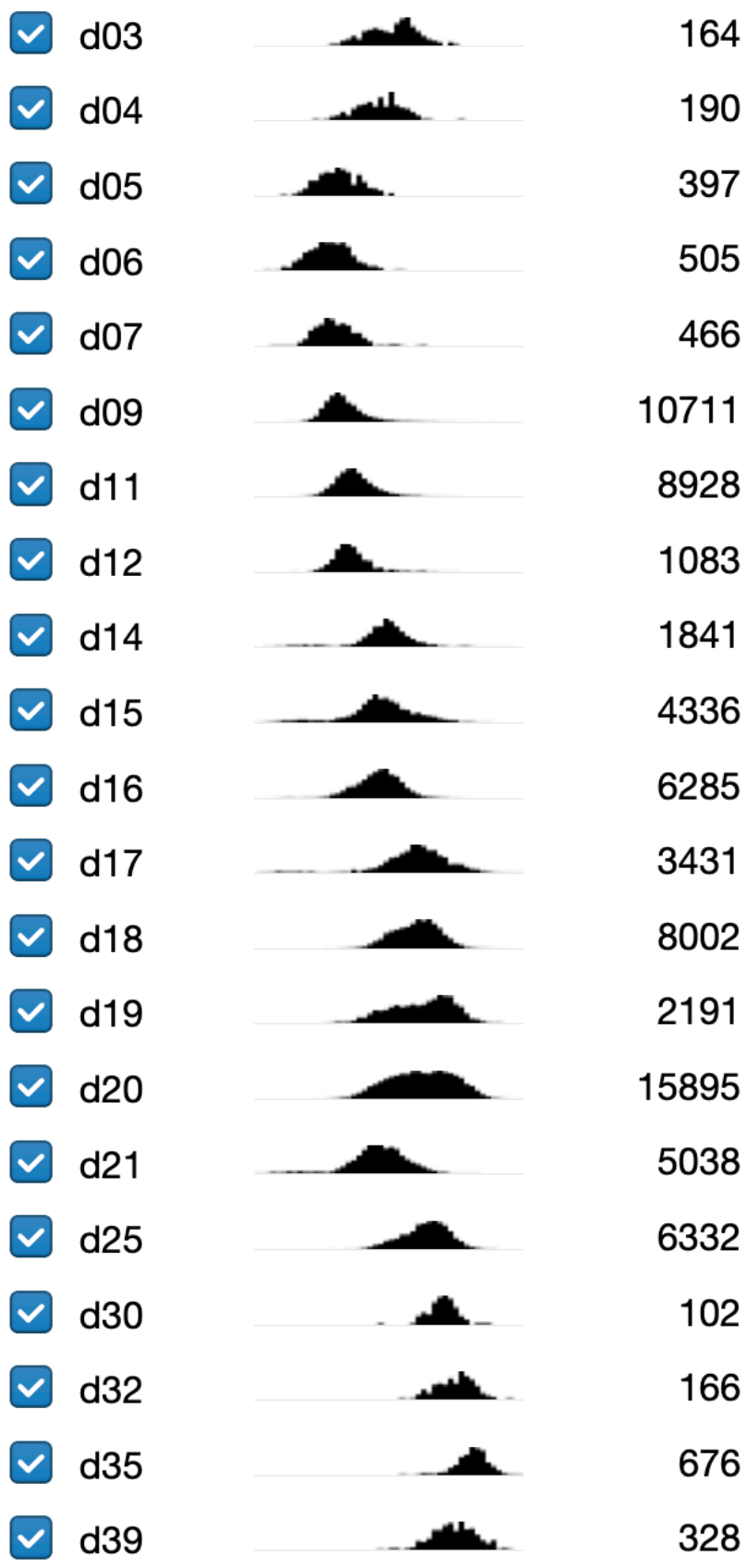

Example 9: CellWhisperer-based identification of organ-specific marker genes

- Search for cells with query term *"heart"*

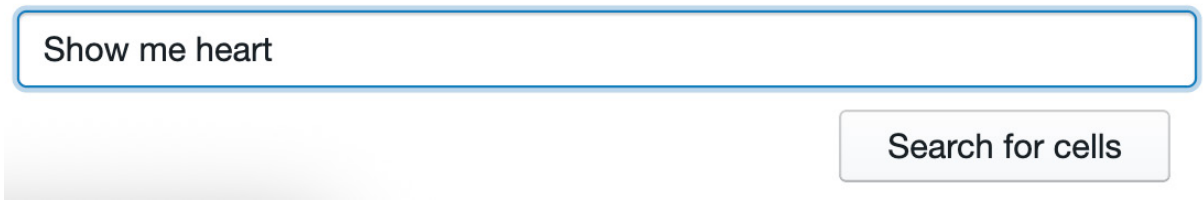

- Select highly scoring cells

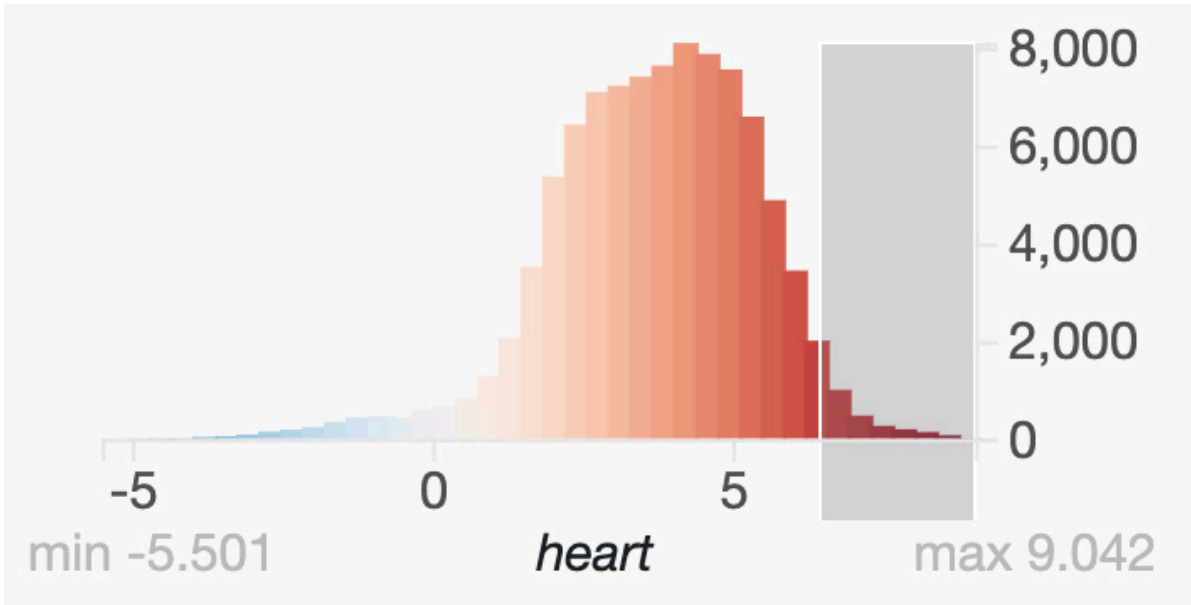

- Retrieve their top-expressed genes

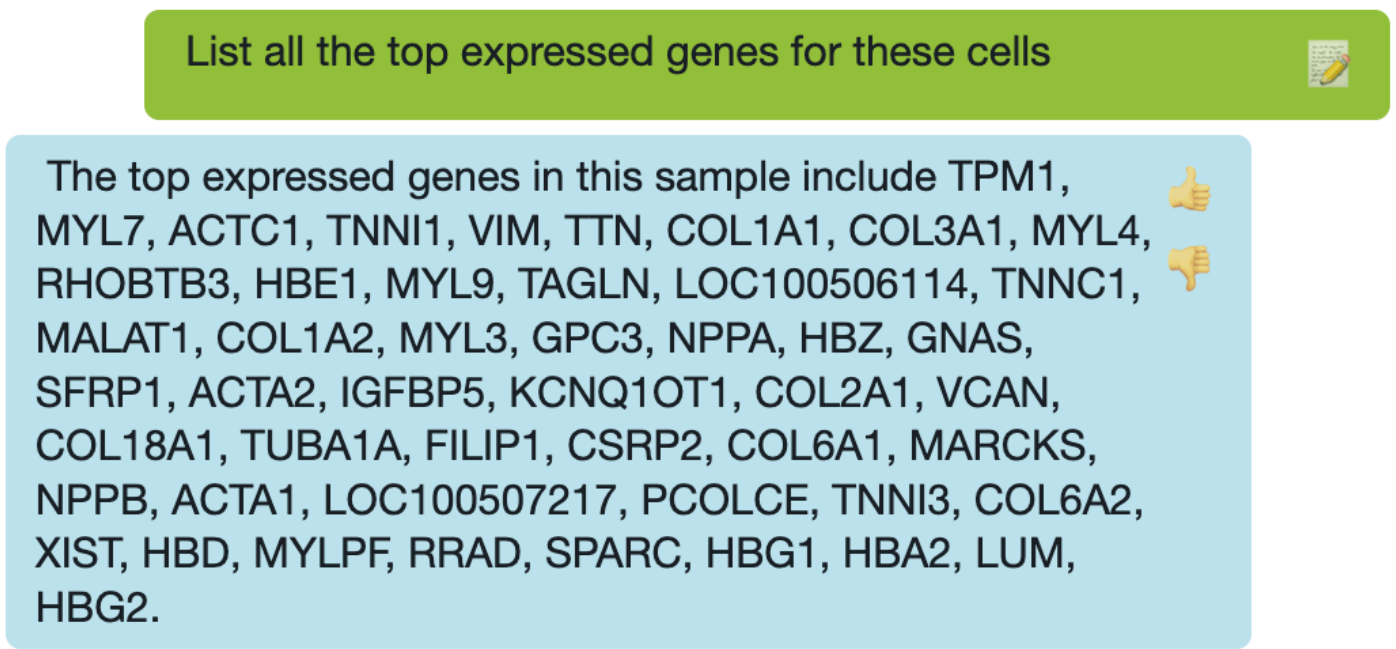

- Cross-check plausibility of CellWhisperer results against external sources

  Many of these genes have well-established roles in heart development and function, and they were confirmed by conventional bioinformatics analysis (**Supplementary Table 1, Fig. 3**).

Supplementary Note 2: Prompts and examples for LLM-assisted curation of the CellWhisperer training data

Training of the CellWhisperer models relies on consistently processed transcriptome metadata for the generation of concise natural-language annotations and chat conversations. In this Supplementary Note, we provide the large language model (LLM) prompts that were used for training data curation, together with examples of input data and the corresponding LLM-curated outputs. For each of the LLM data curation tasks, we provide three types of information: (i) the prompt; (ii) example input data (for consistency, we rely on the same input sample across the different processing steps, which is listed below only once for all prompts); (iii) the LLM-generated output. Further methodological details are provided in the Methods section of the paper.

Table of contents

1. Principles of prompt engineering for CellWhisperer training data curation 16
2. Generation of textual annotations from sample metadata 16
3. Generation of question-answer conversations for the CellWhisperer chat model.............................. 18
4. Inference prompts of the CellWhisperer chat model applied to user-provided data 22

1. Principles of prompt engineering for CellWhisperer training data curation

LLMs are known to depend on well-optimized prompts to achieve best results. We followed an iterative prompt engineering approach, the results of which are shown below, relying on several recently established techniques. First, LLM responses often benefit if the LLM first reiterates the question in an ordered form and recapitulates established facts about the topic; this has been referred to as pre-action reasoning (Yao et al. 2022). We thus instruct the LLM to first gather all relevant information and then aggregate it into a concise response. Second, it is often helpful to assign a hypothetical role to the LLM, encouraging it to respond in the voice of an expert for the task at hand (e.g., "You are a biomedical researcher"); this has been shown to improve the response quality (Kong et al. 2024). Third, to illustrate and further contextualize the instructions in our prompt, we provide the LLM with hand-curated examples of how we expect it to transform inputs into outputs, by appending multiple such examples to the prompt, a technique referred to as few-shot learning (Brown et al. 2020).

2. Generation of textual annotations from sample metadata

Prompt for deriving concise textual annotations from sample metadata (LLM: Mixtral 8x7B, with nine few-shot learning examples available from the CellWhisperer source code repository)

```
You are a biomedical researcher. Your task is to extract the biologically relevant information
from a sample's metadata indicated by <<<>>> and representing them in natural language.

# Instructions

## Represent Biological Information
Only consider information that are reflective of the sample's biology, i.e. the phenotype and
transcriptome of the contained cells, such as cell type or any pertinent conditions or
treatments.

## Study-Level Information
This is just additional context and should not be included in the response, beyond informing
about the the nature of the sample.

## Ignore Non-Biological Information
```

```
Ignore information that is not relevant to the cellular state of the sample. Specifically,
exclude all IDs and details about technical and methodological processes such as sequencing type,
read length, platform, or any other laboratory technique-related information.

## Response Template
Respond in two parts following the JSON template below.
{{
    "1. Relevant Information to be Included": <List[str]: List of information to be extracted,
according to the instructions above>,
    "2. Final Response": <str: brief natural language descripton of the sample focusing solely on
the biological aspects that contribute to understanding its cellular state>,
}}

# Examples
Here are some examples:

{few_shot_block}

# Provided Metadata

<<<
{query}
>>>
```

Input example of metadata for a bulk RNA-seq sample obtained from GEO/SRA with ID SRX7552910

```
geo_title: T2-4
geo_source_name: Renal Cell Carcinoma
raw_biosample_metadata: source_name: Renal Cell Carcinoma; tissue: Renal Cell Carcinoma; tissue
    preparation: nephrectomy specimens preserved in FFPE; metastatic: No; tumor stage: 2; sex: male
treatment:
treatment_protocol: None
series_summary: Presented is the differential expression of mRNA and miRNA in 44 Stage I and Stage
    II patients who developed metastasis within 5 years of nephrectomycompared to 21 patients who
    remained disease free. Extracted RNA from nephrectomy specimens preserved in FFPE blocks was
    sequenced using RNAseq. MiRNA expression was done using the TaqMan OpenArray qPCR protocol.
series_design: Examination of molecular differences between metatstatic versus non-metatstatic in
    stage I and stage II
growth_protocol: None
```

Textual annotation output generated based on the metadata for the bulk RNA-seq sample with ID SRX7552910

```
Renal cell carcinoma tissue sample taken from a male patient at stage 2, with no metastasis,
preserved in FFPE blocks
```

Input example of metadata for scRNA-seq dataset obtained from CELLxGENE Census with ID 1e6a6ef9-7ec9-4c90-bbfb-2ad3c3165fd1) and meta-cell number 3725

```
cell_type: club cell
development_stage: unknown
organism: Homo sapiens
self_reported_ethnicity: unknown
sex: male
suspension_type: cell
tissue: lung
study_description: Non-small cell lung cancer (NSCLC) is characterized by molecular heterogeneity
    with diverse immune cell infiltration patterns which has been linked to therapy sensitivity and
    resistance. However, full understanding of how immune cell phenotypes vary across different
    patient subgroups is lacking. Here, we dissect the NSCLC tumor microenvironment at high reso-
    lution by integrating 1,283,972 single cells from 556 samples and 318 patients across 29
    datasets, including our dataset capturing cells with low mRNA content. We stratify patients
    into immune-deserted, B cell, T-cell, and myeloid cell subtypes. Using bulk samples with genomic
```

```
    and clinical information, we identify cellular components associated with tumor histology and
    genotypes. We then focus on the analysis of tissue-resident neutrophils (TRNs) and uncover
    distinct subpopulations that acquire new functional properties in the tissue microenvironment,
    providing evidence for the plasticity of TRNs. Finally, we show that a TRN-derived gene signature
    is associated with anti-programmed cell death ligand 1 (PD-L1) treatment failure.,
study_title: The single-cell lung cancer atlas (LuCA) -- extended atlas
sample_source_name: Chen_Zhang_2020_NSCLC-5
ann_coarse: Epithelial cell
ann_fine: Club
cell_type_major: Club
cell_type_neutro: Club
cell_type_neutro_coarse: Club
cell_type_predicted: Epithelial cell malignant
cell_type_tumor: Club
dataset: Chen_Zhang_2020
doublet_status: singlet
origin: tumor_primary
origin_fine: tumor_primary
platform: 10x
study: Chen_Zhang_2020
tumor_stage: advanced
uicc_stage: III
```

Textual annotation output generated based on the metadata for the scRNA-seq sample with ID 1e6a6ef9-7ec9-4c90-bbfb-2ad3c3165fd1 and meta-cell number 3725

```
Club cells derived from a primary lung tumor of a male individual with advanced stage non-small
cell lung cancer (NSCLC), UICC stage III.
```

3. Generation of question-answer conversations for the CellWhisperer chat model

Hand-crafted questions for the "simple" and "detailed" conversations

- *What does the sample represent?*
- *What does the transcriptome represent?*
- *What do these cells represent?*
- *Give a brief description of the sample.*
- *Give a brief description of these cells.*
- *Present a compact description of the sample's key features.*
- *Summarize the state of these cells.*
- *Provide a brief description of these cells.*
- *Provide a brief description of the given sample.*
- *Describe the sample concisely.*
- *Describe these cells concisely.*

Prompt for "complex" conversations (LLM: GPT-4 with three few-shot examples; full version provided in the CellWhisperer source code repository)

```
You are an AI molecular analyst with a deep understanding of cells and their states. Your task is
to generate a question and a response that delve into the intricacies of a biological sample of
human cells.

# Instructions

## Provided information
You are equipped with a detailed description of a biological sample of cells, including
- a succinct natural language summary of the biological state of the sample
```

```
- a prioritized list of the most prominently expressed genes (with the highest expression levels
listed first)
- a prioritized list of the most active pathways, processes and other ontological terms (with the
most significant ones listed first)

## First contemplate
First contemplate about the provided information to obtain a biological intuition of the nature o
the provided sample. Use this intuition (rather than the provided raw annotation and gene lists)
to identify an interesting biological question with a matching insightful, but concise, response.
Finish the contemplation with a suggestion for the topic of the question.

## The researcher's question
The researcher possesses knowledge in the biomedical domain (such as molecular biology,
immunology, and cancer biology), but is unfamiliar with the specifics of the provided cellular
sample. They are interested in the sample's phenotype, cell type/state, origin,
treatment/perturbation, active processes/pathways and pose a specific and interesting question
that will unveil any of the contemplated information. Examples for interesting questions:
- How have these cells been treated?
- What are the biological processes and pathways that stand out in this sample?
- Does this sample exhibit pathogenic characteristics?

## The AI system's response
The AI system provides a comprehensive, concise and insightful response to the researcher's
question, drawing from summary in the 'contemplation' step, rather than from the raw provided
information. As the researcher does not have any information about the sample, the response needs
to be phrased as such. Specifically, the AI response should sound like it is directly
investigating the biological state of the cellular sample, without having any additional
information of its origin or processing steps. In line with this, all provided information should
explicitly acknowledge the uncertainty of the AI system (e.g. use phrases like \"it seems\",
\"could be\", \"likely\", \"potentially\", etc.).

## Format
Generate the dialogue as a JSON array, following this schema:

[{\"contemplation\": \"<str: contemplation of the sample's state>\"}, {\"from\": \"researcher\",
\"value\": <str: the researcher's question>}, {\"from\": \"ai\", \"value\": <str: the AI system's
response>}]
```

Input example for "complex", "detailed", and "conversation" conversations (based on data for SRX7552910)

```
Sample annotation: Renal cell carcinoma tissue sample taken from a male patient at stage 2, with
no metastasis, preserved in FFPE blocks.


Gene sets (e.g. pathways, GO terms): Bone Marrow-hematopoietic Stem Cell, Blood-hematopoietic Stem
Cell, Blood-cd141-positive Myeloid Dendritic Cell, Bone Marrow-myeloid Progenitor, Blood-basophil,
Mammary-pericyte Cell, Mammary-vein Endothelial Cell, Thymus-innate Lymphoid Cell, Skin-nkt Cell,
Skin-cd4-positive, Alpha-Beta Memory T Cell, Skin-naive Thymus-Derived Cd4-Positive, Alpha-Beta T
Cell, Skin-naive B Cell, Blood-myeloid Progenitor, Spleen-cd1c-positive Myeloid Dendritic Cell,
Blood-plasmacytoid Dendritic Cell, Spleen-naive Thymus-Derived Cd4-Positive, Alpha-Beta T Cell,
Skin-t Cell, Large Intestine-cd4-positive, Alpha-Beta T Cell, Skin-cd8-positive, Alpha-Beta Memory
T Cell, Skin-cd4-positive Helper T Cell, Skin-nk Cell, Lung-smooth Muscle Cell, Mammary-endothelial
Cell Of Artery, Large Intestine-b Cell, Blood-naive Thymus-Derived Cd4-Positive, Alpha-Beta T Cell,
Lung-cd4-positive, Alpha-Beta T Cell, Blood-plasma Cell, Mammary-b Cell, Bone Marrow-cd4-positive,
Alpha-Beta T Cell, Small Intestine-cd4-positive, Alpha-Beta T Cell, Pancreas-b Cell, Salivary
Gland-cd4-positive Helper T Cell, Thymus-dn3 Thymocyte, Eye-t Cell, Mammary-t Cell, Bone Marrow-
memory B Cell, Thymus-memory B Cell, Lymph Node-regulatory T Cell, Lung-bronchial Vessel Endothelial
Cell, Fat-leucocyte, Small Intestine-cd8-positive, Alpha-Beta T Cell, Vasculature-b Cell, Skin-
cd8-positive, Alpha-Beta Cytotoxic T Cell, Lymph Node-innate Lymphoid Cell, Trachea-cd4-positive,
Alpha-Beta T Cell, Fat-t Cell, Thymus-vein Endothelial Cell, Spleen-cd4-positive, Alpha-Beta Memory
T Cell, Thymus-dn1 Thymic Pro-T Cell, Large Intestine-mast Cell


Top expressed normalized genes: NEB, MUC12, C4A, IGHG1, IGHG1, SCARNA10, SNORD3D, IGLC1, IGFN1,
CTAGE9, IGHG2, UGT2B7, SCARNA2, IGLC2, VWF, IGHGP, ENSG00000243302, USP17L15, NAIPP4, FLG, COL3A1,
```

```
IGLC3, SCARNA7, PKHD1, ENSG00000284454, HRNR, LRRC37A, ARHGEF34P, COL1A2, ENSG00000281380, NAIPP2, ARHGEF35, IGKC, MGAM, MGAM, RGPD1, AHNAK2, NPIPB12, PREX2, PPIAP22, LRRC37A3, ENSG00000215156, SST, SCARNA5, SCARNA5, DNAH11, RGPD3, ENSG00000283738, USP17L6P, C3
```

Generated output for the "complex" conversation prompt and the question-answer input for SRX7552910

- Contemplation: This sample is from a stage 2 renal cell carcinoma (RCC) tissue of a male patient, preserved in formalin-fixed paraffin-embedded (FFPE) blocks. The absence of metastasis suggests localized disease. The gene expression and pathway enrichment reflect a complex interaction between tumor cells and the tumor microenvironment, including immune infiltration and extracellular matrix remodeling. High expression of genes such as VWF (involved in blood clotting and endothelial cell function), COL3A1, COL1A2, and COL1A1 (collagen genes involved in extracellular matrix structure), alongside MUC12 (a mucin gene potentially indicative of a protective barrier or altered cell signaling) suggests active angiogenesis and tissue remodeling. The presence of immune-related pathways and cells indicates an active immune response or immune evasion mechanisms. The gene expression profile points towards a tumor microenvironment that is actively engaged in maintaining the tumor structure and potentially facilitating its growth, despite the localized nature of the disease. The specific characteristics of this RCC sample, such as its stage, cell interactions, and potential vulnerabilities, could be the focus of an interesting question.
- Question (from researcher): What does the gene expression profile indicate about the tumor microenvironment in this RCC sample?
- Response (from AI assistant): The gene expression profile of this stage 2 renal cell carcinoma sample suggests a dynamic and complex tumor microenvironment. There's evidence of significant extracellular matrix remodeling, indicated by the high expression of collagen genes like COL3A1, COL1A2, and COL1A1, which are essential for tissue structure and integrity. The presence of VWF hints at active angiogenesis, which is crucial for tumor nourishment and growth. Additionally, the expression of MUC12 may suggest altered cell signaling or a protective barrier function in the tumor cells. Immune-related pathways and gene sets suggest either an active immune response within the tumor microenvironment or mechanisms of immune evasion by the tumor. Together, these factors underscore the interactions between tumor cells, the extracellular matrix, and the immune system, contributing to the tumor's growth and potential resistance to therapy.

Prompt for "detailed" conversations (LLM: GPT-4, zero-shot)

```
You are an AI molecular analyst with a deep understanding of cells and their states. Your task is
to generate a question and a response that delve into the intricacies of a biological sample of
human cells.

# Instructions

## Provided information
You are equipped with a detailed description of a biological sample of cells, including
- a succinct natural language summary of the biological state of the sample
- a prioritized list of the most prominently expressed genes (with the highest expression levels
listed first)
- a prioritized list of the most active pathways, processes and other ontological terms (with the
most significant ones listed first)

## First contemplate
First contemplate about the provided information to obtain a biological intuition of the nature o
the provided sample. Use this intuition (rather than the provided raw annotation and gene lists)
to identify an interesting biological question with a matching insightful, but concise, response.
Finish the contemplation with a suggestion for the topic of the question.

## The researcher's question
The researcher possesses knowledge in the biomedical domain (such as molecular biology,
immunology, and cancer biology), but is unfamiliar with the specifics of the provided cellular
sample. They are interested in the sample's phenotype, cell type/state, origin,
treatment/perturbation, active processes/pathways and poses the following question: {question}

## The AI system's response
```

```
The AI system provides a comprehensive, concise and insightful response to the researcher's
question, drawing from summary in the 'contemplation' step, rather than from the raw provided
information. As the researcher does not have any information about the sample, the response needs
to be phrased as such. Specifically, the AI response should sound like it is directly
investigating the biological state of the cellular sample, without having any additional
information of its origin or processing steps. In line with this, all provided information should
explicitly acknowledge the uncertainty of the AI system (e.g. use phrases like \"it seems\",
\"could be\", \"likely\", \"potentially\", etc.).

## Format
Generate the dialogue as a JSON array, following this schema:

[{{\"contemplation\": \"<str: contemplation of the sample's state>\"}}, {{\"from\":
\"researcher\", \"value\": <str: the researcher's question>}}, {{\"from\": \"ai\", \"value\":
<str: the AI system's response>}}]
```

Generated output for the "detailed" conversation prompt and the question-answer input for SRX7552910

- Contemplation: The provided sample is from renal cell carcinoma tissue, indicating a cancerous state. The gene expression profile and active pathways suggest a complex interplay of various cell types, including hematopoietic stem cells, myeloid and dendritic cells, T cells, B cells, and endothelial cells, among others. This diversity implies a rich microenvironment within the tumor, potentially reflecting immune infiltration, angiogenesis, and possibly the presence of cancer stem cells. The high expression of genes like NEB, MUC12, and C4A, among others, could be indicative of specific cancer-related processes such as cell adhesion, immune response modulation, and extracellular matrix remodeling. The presence of genes associated with hematopoiesis and immune cells suggests an active immune response or infiltration within the tumor microenvironment. The challenge lies in deciphering the roles these diverse cell types and pathways play in tumor progression, immune evasion, and their potential as therapeutic targets or biomarkers.
- Question (from researcher): Describe these cells concisely.
- Response (from AI assistant): The sample appears to be from a renal cell carcinoma at stage 2, characterized by a complex tumor microenvironment. This environment includes a variety of cell types such as hematopoietic stem cells, myeloid and dendritic cells, T cells, B cells, and endothelial cells, suggesting immune infiltration and angiogenesis. The gene expression profile, with genes like NEB, MUC12, and C4A highly expressed, points towards processes such as cell adhesion, immune response modulation, and extracellular matrix remodeling. These features could reflect the tumor's interaction with its microenvironment, including immune evasion mechanisms and potential angiogenesis. The diversity of cell types and active pathways indicates a rich, dynamic interplay within the tumor, which could have implications for therapy and prognosis.

Prompt for "conversation" conversations (Mixtral 8x7B with five few-shot examples; full version provided in the CellWhisperer source code repository)

```
You are a molecular analyst and play writer with a deep understanding of cellular states and
phenotypes. Your task is to generate a realistic conversation between a biomedical researcher and
an AI assistant, chatting about a biological sample of human cells.

# Instructions

## Provided information
You are given a description of a biological sample of cells, that represents its state/phenotype.
This description includes
- a concise natural language annotation of the biological state of the sample
- a ranked list of the most strongly expressed genes (most upregulated genes come first)
- a ranked list of the most active pathways and enriched gene ontological terms (most signifcant
ones come first)

## The researcher's questions
The researcher has an interest in molecular biology, immunology, cancer biology, etc. and
initially doesn't know anything about the provided biological sample. He asks questions to learn
about the biological nature of the sample. The questions should be relevant, diverse, addressable
(given the provided information) and in the natural tone of a scientist curious about the
```

```
nature/biology of the sample. Specifically the researcher uses simple and conversational
language, asking questions on the biology of the sample (e.g. active pathways, processes,
mechanisms, regulation patterns etc.). The researcher is not interested in the donor of the
cells.

## The AI assistant's responses
The AI assistant's task is to help the researcher learn about the sample's biological nature. The
AI assistant has access only to a snapshot of the biological state of cellular sample
(represented via the provided information) and thereby can only respond with information that can
logically be derived from such a logical snapshot (e.g. no specific dosage numbers of treatments,
unimportant processing steps are likely to be reflected in such a snapshot, nor any donor- or
patient-specific information such as age or sex). Likewise, the AI assistant does not respond by
enumerating the provided genes and gene sets, but rather provides a more conceptual and
summarized form of the provided data, that describes the overall nature of the sample.
Furthermore, the AI assistant uses a tone of reflection and reasoning and without absolute
certainty (e.g. "It seems that ..."), and, where applicable, the AI assistant should include
reasoning steps to make the content more convincing and well-organized.
The responses must be fully in accordance with the provided information about the sample. If the
researcher asks a question that cannot be answered with the provided information, the AI
assistant should indicate so.

## Additional instructions
- refer to the data as "this sample" or "this cell type" or "these cells"
- keep it simple. no need to cover all information
- do not include any identifiers such as sample names, cell line names, ontology identifiers,
etc.
- Avoid the term 'gene sets'

## Format
Generate the conversation in the JSON format, following this schema:

[{"from": "researcher", "value": <str: the researcher's question>}, {"from": "ai", "value": <str:
the AI assistant's response>}, ...]
```

Generated output for the "conversation" conversation prompt and the question-answer input for SRX7552910

Researcher: What type of tissue does this sample represent?

AI assistant: This sample originates from renal tissue, specifically from a renal cell carcinoma tumor.

Researcher: What can you tell me about the state of this renal tissue?

AI assistant: Based on the provided information, the renal tissue appears to be cancerous, specifically classified as renal cell carcinoma at stage 2 without metastasis.

Researcher: What genes are highly expressed in this renal tissue?

AI assistant: The top expressed genes in this renal tissue include NEB, MUC12, C4A, IGHG1, IGHG1, SCARNA10, SNORD3D, IGLC1, IGFN1, CTAGE9, IGHG2, UGT2B7, SCARNA2, IGLC2, VWF, IGHGP, ENSG00000243302, USP17L15, NAIPP4, FLG, COL3A1, IGLC3, SCARNA7, PKHD1, ENSG00000284454, HRNR, LRRC37A, ARHGEF34P, COL1A2, ENSG00000281380, NAIPP2, ARHGEF35, IGKC, MGAM, RGPD1, AHNAK2, NPIPB12, PREX2, PPIAP22, LRRC37A3, ENSG00000215156, SST, SCARNA5, DNAH11, RGPD3, ENSG00000283738, USP17L6P, C3.

4. Inference prompts of the CellWhisperer chat model applied to user-provided data

Prompt for the generation of cell cluster descriptions using the CellWhisperer chat model

User:

```
Help me analyzing this sample of cells. Respond in proper English sentences in a tone of
uncertainty and focus on the biology of the sample rather than any potential donor or patient
information (e.g. do not mention age and sex)
```

Assistant:

```
Sure. I'll respond as you requested, focusing on the sample of cells and avoiding patient or
donor information.
```

User:

```
Describe the biological state of these cells
```

Prompt for condensing the generated cluster descriptions with GPT-4

```
Condense this description of a cell sample into a short title (using normal sentence case) of
maximum 8 words. Focus on the biological state of the sample, rather than its source or any
specific perturbations.
```

Pre-prompt for the CellWhisperer chat model (employed in web app)

User:

```
Help me analyzing this sample of cells. Respond in proper English in a tone of uncertainty and
focus on the biology of the sample rather than any potential donor or patient information (e.g.
do not mention age and sex). Start by listing the top 50 genes.
```

Assistant:

```
Sure. I'll respond as you requested, focusing on the sample of cells and avoiding any personal
information. It looks like the 20 top normalized genes are <top20 genes>.
Still remarkably strong expressed seem the genes <top20-50 genes>. Note that there are even more
strongly expressed genes beyond the ones I just listed.
```

User:

```
<user chat input>
```

Supplementary Note 3: Ablation study for the CellWhisperer embedding model

The CellWhisperer embedding model is based on various machine learning and data processing techniques. Here we analyze the importance of the individual techniques and model components, by training and testing adapted versions of our models that lacked one component at a time, in a process referred to as ablation (**Ext. Data Fig. 1c**). We assessed the performance of these models on five representative metrics introduced in **Fig. 2**. For comparability and computational efficiency, all models were trained in four epochs with three different seeds. Below we describe each tested ablation and summarize the observed effects on performance.

First, the CellWhisperer embedding model follows the LiT method (Zhai et al. 2021), by initializing the network architecture and weights of the modality-specific embedding models based on pretrained transcriptome (Geneformer) and text (BioBERT) models, while freezing the former model during training. We tested the impact of different freezing configurations on the model performance. We observed that freezing both models or unfreezing only the transcriptome model led to a significant loss of performance, whereas freezing only the transcriptome model resulted in the best performance among the tested configurations.

Second, we tested the use of an alternative transcriptome model as part of the CellWhisperer embedding model. The scGPT model has achieved slightly better performance compared to Geneformer on various single-cell interpretation tasks (Cui et al. 2024; Kedzierska et al. 2023). We built an scGPT-based version of CellWhisperer and indeed observed increased performance on cell type prediction tasks. In contrast, the Geneformer-based version performed better in zero-shot disease prediction, indicating better ability to retain diverse biological information (beyond cell types) in the transcriptome embedding for diverse prediction tasks.

Third, we explored the effect of replacing the pretrained transcriptome model with a feed-forward neural network operating on the fixed-order gene expression vector and trained from scratch. As expected, results were substantially worse for the simpler model, but even that model retained better-than-random performance.

Fourth, we investigated the effect of training batch size on model performance, as it has previously been shown that contrastive training relies on particularly large batch sizes (Chen et al. 2020). We observed a significant decrease in performance when reducing the batch sizes by a factor of 16 (from 512 to 32), even when the gradients were accumulated across bags of 16 consecutive batches. We did not observe substantially improved results with larger batch sizes, nor with an alternative contrastive loss formulation (Jensen-Shannon divergence loss), which was described to perform well in low data and low batch size regimes (Shrivastava et al. 2023).

Fifth, we assessed the performance of multimodal models that were trained solely on data from one of the two data repositories (GEO or CELLxGENE Census). These models retained strong performance for most metrics, indicating that both repositories contain sufficiently broad information to support tasks such as zero-shot cell type prediction. Nevertheless, excluding the GEO dataset led to substantially reduced performance for disease prediction, which is likely due to the low representation of disease samples in CELLxGENE Census.

Sixth, we tested whether training data augmentation improves model performance. To that end, we generated slight variations of the textual annotations (based on different prompts in the LLM-assisted curation of the training data) and added individual single-cell profiles on top of the pseudo-bulk transcriptome profiles. Previous work on image-based CLIP models supports the value of data augmentation (Fan et al. 2023), but for CellWhisperer we did not observe a clear performance increase, hence we did not further pursue this strategy.

Finally, given the inherent biases of our training data (largely due to the unequal attention that different areas of biology have received and the resulting biases in the RNA-seq data submitted to GEO and other databases), we systematically increased the training weights of samples in areas with low dataset coverage, similarly to recent work on counter-balancing dataset biases in contrastive learning (Alabdulmohsin et al. 2024). However, we observed negligible differences compared to unweighted training, indicating that different sample densities in our training data did not significantly affect model performance as assessable through our metrics.

In summary, these analyses highlight the performance contributions of different aspects of our model architecture and training strategies, providing insights into the design of multimodal transcriptome-text models.

References

Alabdulmohsin, Ibrahim, Xiao Wang, Andreas Steiner, Priya Goyal, Alexander D'Amour, and Xiaohua Zhai. 2024. "CLIP the Bias: How Useful Is Balancing Data in Multimodal Learning?" *ArXiv [Cs.LG]*. https://doi.org/10.48550/arXiv.2403.04547.

Bernstein, Matthew N., Anhai Doan, and Colin N. Dewey. 2017. "MetaSRA: Normalized Human Sample-Specific Metadata for the Sequence Read Archive." *Bioinformatics* 33 (18): 2914–23.

Brown, Tom B., Benjamin Mann, Nick Ryder, Melanie Subbiah, Jared Kaplan, Prafulla Dhariwal, et al. 2020. "Language Models Are Few-Shot Learners." *ArXiv [Cs.CL]*. http://arxiv.org/abs/2005.14165.

Chen, Ting, Simon Kornblith, Mohammad Norouzi, and Geoffrey Hinton. 2020. "A Simple Framework for Contrastive Learning of Visual Representations." ArXiv [Cs.LG]. arXiv. http://arxiv.org/abs/2002.05709.

Cui, Haotian, Chloe Wang, Hassaan Maan, Kuan Pang, Fengning Luo, Nan Duan, and Bo Wang. 2024. "ScGPT: Toward Building a Foundation Model for Single-Cell Multi-Omics Using Generative AI." Nature Methods, 1–11.

Fan, Lijie, Dilip Krishnan, Phillip Isola, Dina Katabi, and Yonglong Tian. 2023. "Improving CLIP Training with Language Rewrites." ArXiv [Cs.CV]. https://doi.org/10.48550/arXiv.2305.20088.

Kedzierska, Kasia Z., Lorin Crawford, Ava P. Amini, and Alex X. Lu. 2023. "Assessing the Limits of Zero-Shot Foundation Models in Single-Cell Biology." BioRxiv. https://doi.org/10.1101/2023.10.16.561085.

Kong, Aobo, Shiwan Zhao, Hao Chen, Qicheng Li, Yong Qin, Ruiqi Sun, Xin Zhou, Enzhi Wang, and Xiaohang Dong. 2024. "Better Zero-Shot Reasoning with Role-Play Prompting." In *Proceedings of the 2024 Conference of the North American Chapter of the Association for Computational Linguistics: Human Language Technologies (Volume 1: Long Papers)*, 4099–4113. Mexico City, Mexico.

Shrivastava, Aman, Ramprasaath R. Selvaraju, Nikhil Naik, and Vicente Ordonez. 2023. "CLIP-Lite: Information Efficient Visual Representation Learning with Language Supervision." In *Proceedings of The 26th International Conference on Artificial Intelligence and Statistics*, 206:8433–47. Proceedings of Machine Learning Research.

Yao, Shunyu, Jeffrey Zhao, Dian Yu, Nan Du, Izhak Shafran, Karthik Narasimhan, and Yuan Cao. 2022. "ReAct: Synergizing Reasoning and Acting in Language Models." *ArXiv [Cs.CL]*. https://doi.org/10.48550/arXiv.2210.03629.

Zhai, Xiaohua, Xiao Wang, Basil Mustafa, A. Steiner, Daniel Keysers, Alexander Kolesnikov, and Lucas Beyer. 2021. "LiT: Zero-Shot Transfer with Locked-Image Text Tuning." *Proceedings / CVPR, IEEE Computer Society Conference on Computer Vision and Pattern Recognition. IEEE Computer Society Conference on Computer Vision and Pattern Recognition*, November, 18102–12.
