# Supplementary material for: Multimodal learning enables chat-based exploration of single-cell data
Source: Nat Biotechnol. 2025 Nov 11;44(10):1722–32. doi: 10.1038/s41587-025-02857-9 (PMC13654808; doi:10.1038/s41587-025-02857-9)
Supplement: Supplementary file 2 [file 41587_2025_2857_MOESM2_ESM.pdf]

nature portfolio

Corresponding author(s): Christoph Bock

Last updated by author(s): Sep 5, 2025

Reporting Summary

Nature Portfolio wishes to improve the reproducibility of the work that we publish. This form provides structure for consistency and transparency in reporting. For further information on Nature Portfolio policies, see our Editorial Policies and the Editorial Policy Checklist.

Statistics

For all statistical analyses, confirm that the following items are present in the figure legend, table legend, main text, or Methods section.

n/a	Confirmed	
☐	☒	The exact sample size (*n*) for each experimental group/condition, given as a discrete number and unit of measurement
☒	☐	A statement on whether measurements were taken from distinct samples or whether the same sample was measured repeatedly
☐	☒	The statistical test(s) used AND whether they are one- or two-sided<br>*Only common tests should be described solely by name; describe more complex techniques in the Methods section.*
☒	☐	A description of all covariates tested
☒	☐	A description of any assumptions or corrections, such as tests of normality and adjustment for multiple comparisons
☒	☐	A full description of the statistical parameters including central tendency (e.g. means) or other basic estimates (e.g. regression coefficient) AND variation (e.g. standard deviation) or associated estimates of uncertainty (e.g. confidence intervals)
☐	☒	For null hypothesis testing, the test statistic (e.g. *F*, *t*, *r*) with confidence intervals, effect sizes, degrees of freedom and *P* value noted<br>*Give P values as exact values whenever suitable.*
☒	☐	For Bayesian analysis, information on the choice of priors and Markov chain Monte Carlo settings
☒	☐	For hierarchical and complex designs, identification of the appropriate level for tests and full reporting of outcomes
☐	☒	Estimates of effect sizes (e.g. Cohen's *d*, Pearson's *r*), indicating how they were calculated

Our web collection on statistics for biologists contains articles on many of the points above.

Software and code

Policy information about availability of computer code

Data collection	The following open-source software package was used for data collection: fetchngs (1.12.0)
Data analysis	The following open-source software packages were used for data analysis (obtained via pypi and conda-forge): anndata (0.10.2), llama-cpp-python (0.2.77), transformers (4.37.2), numpy (1.26.4), pytorch (2.1.0), lightning (2.1.0), scanpy (1.9.5), scipy (1.12.0), snakemake (7.32.4), torchmetrics (1.2.0), scikit-misc (0.1.4), umap-learn (0.5.3), datasets (2.12.0), scib (1.1.4), louvain (0.8.1), biopython (1.83), igraph (0.11.4), leidenalg (0.10.2), gseapy (1.0.6), scvi-tools (0.20.3), umap-learn (0.5.5), matplotlib_venn (0.11.10), rnaseq (3.7)<br><br>The source code underlying the data analysis is provided at: https://github.com/epigen/cellwhisperer

For manuscripts utilizing custom algorithms or software that are central to the research but not yet described in published literature, software must be made available to editors and reviewers. We strongly encourage code deposition in a community repository (e.g. GitHub). See the Nature Portfolio guidelines for submitting code & software for further information.

Data

Policy information about availability of data

All manuscripts must include a data availability statement. This statement should provide the following information, where applicable:

- Accession codes, unique identifiers, or web links for publicly available datasets
- A description of any restrictions on data availability
- For clinical datasets or third party data, please ensure that the statement adheres to our policy

CellWhisperer was trained on publicly available datasets obtained from GEO and CELLxGENE Census. All training data are available from their original sources. Full details are provided in the Methods section and in the CellWhisperer source code, which also supports automatic data download.

The model weights and LLM-curated datasets for training and model evaluation are available from the project website: https://cellwhisperer.bocklab.org

Validation datasets were obtained from the following sources (further details are provided in the Methods section and in the CellWhisperer source code):
- Pancreas dataset: https://figshare.com/ndownloader/files/43480497)
- Colonic epithelium dataset: GEO (GSE116222)
- Immgen dataset: https://sharehost.hms.harvard.edu/immgen/GSE227743/GSE227743_Gene_count_table.csv
- Tabula Sapiens dataset: https://figshare.com/ndownloader/files/40067134
- Human diseases dataset: 14112 samples derived from GEO through querying MetaSRA for disease samples
- Human development dataset: EBI-ENA (PRJEB30442, PRJEB40781); EBI-ArrayExpress (E-MTAB-3929, E-MTAB-8060), GEO (GSE232861, GSE155121)
- AIDA: https://datasets.cellxgene.cziscience.com/ff5a921e-6e6c-49f6-9412-ad9682d23307.h5ad

Research involving human participants, their data, or biological material

Policy information about studies with human participants or human data. See also policy information about sex, gender (identity/presentation), and sexual orientation and race, ethnicity and racism.

Reporting on sex and gender	N/A
Reporting on race, ethnicity, or other socially relevant groupings	N/A
Population characteristics	N/A
Recruitment	N/A
Ethics oversight	N/A

Note that full information on the approval of the study protocol must also be provided in the manuscript.

Field-specific reporting

Please select the one below that is the best fit for your research. If you are not sure, read the appropriate sections before making your selection.

☒ Life sciences ☐ Behavioural & social sciences ☐ Ecological, evolutionary & environmental sciences

For a reference copy of the document with all sections, see nature.com/documents/nr-reporting-summary-flat.pdf

Life sciences study design

All studies must disclose on these points even when the disclosure is negative.

Sample size	We trained the CellWhisperer embedding model on 1,082,413 pairs of transcriptomes and their textual annotations, which were prepared with AI-assisted curation from two large community-wide repositories: Gene Expression Omnibus (GEO) and CELLxGENE Census. LLM fine-tuning was performed on a subset of 106,610 data points, which is an adequate sample size according to a previous report (H. Liu et al. 2023).
Data exclusions	A small fraction of the training data were filtered out because of low read count numbers, as measured by the number of expressed genes.
Replication	We trained the AI model with multiple seeds to assess the stability of our training procedure and model architecture (Supplementary Note 2). Moreover, we regenerated all results shown in the paper once it was accepted in principle and confirmed that the reported scores and figures were reproducible with retrained models (with the expected small deviations due to stochasticity where random seeds were used).
Randomization	We analyzed entire datasets - except for a few very large datasets, which were randomly subsampled once for computational efficiency.
Blinding	We trained AI models on labeled datasets from public repositories and tested their zero-shot prediction performance in held-out datasets with ground truth annotations. All reported prediction performance metrics were computed on training-excluded test sets.

Reporting for specific materials, systems and methods

We require information from authors about some types of materials, experimental systems and methods used in many studies. Here, indicate whether each material, system or method listed is relevant to your study. If you are not sure if a list item applies to your research, read the appropriate section before selecting a response.

Materials & experimental systems

n/a	Involved in the study
☒	☐ Antibodies
☒	☐ Eukaryotic cell lines
☒	☐ Palaeontology and archaeology
☒	☐ Animals and other organisms
☒	☐ Clinical data
☒	☐ Dual use research of concern
☒	☐ Plants

Methods

n/a	Involved in the study
☒	☐ ChIP-seq
☒	☐ Flow cytometry
☒	☐ MRI-based neuroimaging

Plants

Seed stocks	N/A
Novel plant genotypes	N/A
Authentication	N/A
